# Comparative Analysis of the Intestinal Microbiota in Wild and Aquaculture Populations of *Sparus aurata*

**DOI:** 10.3390/microorganisms14030708

**Published:** 2026-03-21

**Authors:** Maria Lanara, Elias Asimakis, Naima Bel Mokhtar, Pinelopi Koutsodima, Costas Batargias, Kosmas Toskas, Panagiota Stathopoulou, George Tsiamis

**Affiliations:** 1 Laboratory of Systems Microbiology and Applied Genomics, Department of Sustainable Agriculture, School of Agricultural Sciences, University of Patras, 2 George Seferi St., 30131 Agrinio, Greece; mlanara@hotmail.com (M.L.); eliasasim@upatras.gr (E.A.); naima.belmokhtar@upatras.gr (N.B.M.); pinelopi.ku@gmail.com (P.K.); 2 Laboratory of Applied Genetics and Fish Breeding, Department of Biology, School of Natural Sciences, University of Patras, 26504 Rio, Greece; cbatargias@upatras.gr; 3 Department of Research and Development, Avramar Aquaculture SA, 19.3 km Markopoulou-Paiania Avenue, 19002 Paiania, Greece; k.toskas@avramar.eu; 4 BioDetect P.C., Stadiou St., Platani, 26504 Rio, Greece

**Keywords:** gut microbiota, seabream, 16S rRNA, aquaculture, wild fisheries, core microbiome, Mediterranean aquaculture, probiotic

## Abstract

Fish host complex intestinal bacterial communities that contribute to a wide range of functions, from nutrient assimilation to modulation of the immune system. Understanding how environmental and host-related factors shape the fish gut microbiota is essential for advancing sustainable aquaculture practices. This study compared the intestinal microbiota of gilthead sea bream (*Sparus aurata*) between wild and aquaculture populations in western Greece using 16S rRNA gene amplicon sequencing targeting the V3–V4 region, combined with culture-based methods. The analysis was based on a 97% similarity threshold and included 141 gastrointestinal samples of fish collected at two aquaculture facilities and two wild fisheries, representing two different growth phases (150 g and 300 g body weight). High-throughput sequencing data revealed a clear separation of gut microbial communities according to origin (wild vs. aquaculture), geographic location, and body growth phase, with most wild fish groups exhibiting higher microbial diversity than their farmed counterparts, except for group MES_150 which showed similar or lower values. The gut microbiota was dominated by Pseudomonadota (53%), Bacillota (29%), Actinomycetota (7%), Deinococcota (5%), and Bacteroidota (4%). A shared core microbiome, comprising *Psychrobacter*, *Staphylococcus*, *Geobacillus*, *Aeromonas*, *Enterobacter*, *Pantoea*, *Bacillus*, and *Acinetobacter*, was detected across all populations. Wild fish were enriched in *Psychrobacter*, *Aeromonas*, and *Photobacterium*, while aquaculture fish displayed higher abundances of *Vibrio*, *Allomeiothermus*, and *Staphylococcus*. Network analysis revealed mostly mutually exclusive interactions in both groups but distinct patterns of co-occurrence, driven mainly by *Paenibacillus*, *Enterobacter*, and *Staphylococcus* in wild samples, and by *Vibrio*, *Aeromonas*, and *Pseudomonas* in farmed fish. Culture-based assays demonstrated greater diversity in wild fish, dominated by *Pseudomonas*, *Staphylococcus*, and *Vibrio* strains, in contrast to the frequent occurrence of *Staphylococcus* and *Psychrobacter* in aquaculture samples. The findings suggest that aquaculture practices significantly alter gut microbial community structure and reduce diversity, with potential implications for fish health and disease resistance. The identified core and differentially abundant taxa provide candidates for probiotic development to improve aquaculture sustainability.

## 1. Introduction

Gilthead sea bream (*Sparus aurata* L.) is the most widely farmed fish species in the Mediterranean and Black Sea, with production reaching 281,914 tons in 2020–2021, representing approximately 34% of the region’s total aquaculture output [1]. Greece is the second largest producer in the area, contributing 58,000 tons in 2024, which is equivalent to 18% of the total volume. Despite a 12% decline in gilthead sea bream production compared to the previous year, its sales value increased by 3%, reaching a total of €721 million in combination with European sea bass sales (*Dicentrarchus labrax*). This growth underscores the economic resilience of the domestic aquaculture sector and its contribution to rural employment and coastal communities [2]. With the continuous rise in demand for aquatic animal food, reflected in an estimated 20.7 kg of aquatic animal protein consumed per person annually, the role of aquaculture is becoming increasingly vital for global nutrition and food security [3,4,5]. In 2022, global aquaculture production of animal species surpassed that of capture fisheries for the first time, with 94.4 million tons compared to 91 million tons, showing increasing trends in contrast to capture fisheries production that has remained stable for the past 35 years [3,6]. Similar trends have been observed in Greece, where aquaculture production surpassed capture fisheries in 2003 and currently accounts for 69% of domestic fish production [2]. However, capture fisheries still supply the majority of marine animals that are intended for human consumption, while concerns about the state of their stocks remain high [3]. As aquaculture intensifies to reduce pressure on wild fish stocks and meet the growing global demand for seafood, it must address key challenges related to environmental sustainability, infectious disease outbreaks, and the impacts of climate change. Understanding the gut microbiota of farmed fish species has emerged as a promising path for addressing these challenges, as microbiome-based tools may prove useful for safeguarding and improving the health of farmed fish, enhancing growth and production, and restoring ecosystem health [7,8,9,10].

Fish host complex and dynamic communities of microorganisms on their skin and internal organs, with which they form mutualistic, commensal, or pathogenic relationships [11,12]. This microbiota is in a constant bidirectional interaction with the surrounding environment, particularly within the densely populated, and intensively managed conditions of aquaculture systems [8,13,14]. To support sustainable aquaculture practices, research has focused on the gut microbiota, given that the intestinal tract is continuously exposed to water and food particles, which are rich reservoirs of microbial diversity that may contain opportunistic pathogens or commensals [9,10,15]. The fish intestinal microbiota comprises two distinct components: the resident (autochthonous) microbiota, which is attached to the surface of the gut epithelium, and the transient microbiota (allochthonous), which is present in the ingested food and water within the gut lumen [15]. Intensive research efforts have revealed the multifaceted functional roles of the intestinal microbiota in fish, with emphasis on bacteria, that appear to dominate these assemblages [14]. In teleosts such as *S. aurata*, resident gut bacteria have been implicated in nutritional and energy metabolism, immune regulation and resistance against pathogens, host development, regulation of the gut–brain axis, as well as endocrine and neuronal signaling [11,16,17,18,19]. The important role of symbiotic microorganisms in fish digestion has been well documented, as intestinal microbiota may contribute to nutrient processing and digestive enzyme activity [19]. These communities are typically characterized by high species diversity, and population densities ranging from 10^4^ to 10^9^ colony forming units per gram (CFU × g^−1^) of intestinal content. They are mainly composed of bacterial species belonging to the phyla Pseudomonadota (Proteobacteria), Bacillota (Firmicutes), Bacteroidota (Bacteroidetes), Fusobacteriota (Fusobacteria), and Actinomycetota (Actinobacteria). This diverse community structure and composition may be shaped by a combination of intrinsic factors, such as host species, genetics, and developmental stage, and extrinsic factors, including diet, environmental conditions, and management practices [11,14,16,17]. In gilthead sea bream, the intestinal bacterial communities follow the general trend observed in teleost fish and are characterized by the predominance of Pseudomonadota, Bacillota, Actinomycetota, and Bacteroidota [20,21,22,23].

The main concerns regarding the productivity and sustainability of gilthead sea bream aquaculture are associated with the cost and limited availability of dietary ingredients such as fish meal and fish oil, morphological deformities, hatchery conditions, and pathogen outbreaks [24,25]. Alternative diets based on plant ingredients [20,26,27], low trophic marine species such as microalgae [23,28,29,30], animal by-products [27,31], insects [25,32] or their combinations [33,34] have been employed to partially or fully substitute fish meal and fish oil. These manipulations typically result in variations in the gut microbial communities and favor the prevalence of bacteria related to the metabolism of nutritional ingredients such as carbohydrates, cellulose, hemicellulose, lipids, proteins, and chitin [23,25,30,32,35]. Bacteria with such metabolic capacity could be used as probiotics, alone or in combination with other supplements, to improve digestion of novel diets or provide additional nutrients to farmed fish. Probiotics may also stimulate immune responses and enhance resistance against pathogens [36]. Importantly, probiotics may serve as alternatives to antibiotics which are widely applied in aquaculture to contain disease outbreaks but negatively affect non-target gut microbiota and are limited by the development of resistance [10,36]. Diseases, such as vibriosis or aeromonosis, constitute major threats for Mediterranean aquaculture, inducing septicemia, hemorrhaging, ulcerative lesions and high mortality, especially in early life stages [37,38,39,40]. Understanding how the microbial community and its interactions suppress or facilitate the establishment of opportunistic *Vibrio* and *Aeromonas* species may prove useful for developing microbiome or probiotic-based mitigation strategies for these pathogens. Previous application of probiotic *Bacillus*, *Lactobacillus*, *Lactococcus*, and *Shewanella* strains in gilthead sea bream aquaculture improved growth performance and digestive activity, modulated immune responses and facilitated damaged skin regeneration [21,28,41,42,43,44,45,46].

Comparative analyses of the gut microbiota of farmed fish with their wild counterparts may provide meaningful insight into microbial signatures that may enhance feed efficiency, disease resistance, or tolerance to environmental stressors [11,47]. Such comparisons may be particularly informative, as wild populations typically harbor microbial communities that differ significantly from those of farmed fish and remain unaffected by intensive aquaculture practices [8,11]. Wild fish are exposed to diverse natural diets and environmental conditions that may promote the establishment of beneficial microbes absent in aquaculture settings. Additional parameters, including habitat and environmental conditions, geographic isolation, host developmental stage and spatial distribution along the intestinal tract, may also contribute to the development of unique bacterial characteristics [8,14,15,17,48]. Understanding how these factors shape the gut microbiota is essential for developing targeted interventions to improve the health and productivity of farmed fish.

In this context, this study aimed to characterize and compare the gut microbiota of wild and aquaculture *S. aurata* populations from western Greece. The inclusion of four populations provides a broader ecological context, which may reveal both location-specific microbial signatures shaped by local conditions, as well as core bacterial taxa shared across environments. Similarly, the comparison of two body weight groups offers insight into whether growth-related physiological differences lead to the development of similar or divergent microbial profiles. The analysis of two distinct parts of the gastrointestinal tract, the digestion and absorption regions, allows the characterization of microbial taxa linked to specific functional roles within the *S*. *aurata* gut ecosystem. Co-occurrence network analysis attempts to move beyond taxonomic profiling. This approach enables the identification of keystone taxa and bacterial consortia that may contribute to host nutrition and the overall stability of the community or interact with opportunistic pathogenic species. The specific objectives were to: (i) assess differences in gut microbial diversity between wild and aquaculture populations; (ii) evaluate the effects of geographic location, body-growth stage, and tissue localization on microbial community structure; (iii) identify core microbiome members shared across populations and conditions; (iv) characterize microbial interaction networks within and between experimental groups; and (v) isolate culturable bacteria as candidates for future probiotic evaluation. To address these objectives, we employed high-throughput 16S rRNA gene sequencing to assess the microbial diversity, composition, and interactions of the bacterial communities from four distinct *S. aurata* populations across two body-weight groups and two regions (digestion and absorption sections) of the gastrointestinal tract. Additionally, culture-based methods were used to further explore community diversity and recover candidate strains for future probiotic evaluation.

## 2. Materials and Methods

### 2.1. Sample Collection and Dissection

Four different sampling areas in western Greece—two aquaculture facilities and two wild fisheries—were analyzed. For each site, two body-weight categories were included in the analysis, corresponding to 150 ± 5 g and 300 ± 5 g. The aquaculture individuals were collected from commercial fish farms located in the areas of Astakos (AST) and Vonitsa (VNT) on 31 October 2017 (Supplementary Figure S1). Both populations shared similar genetic backgrounds as they originated from the same broodstock. They were reared in open sea cages with a diameter of 12 m and a depth of 9 m (~1000 m^3^) and fed twice per day with a commercial synthetic diet. Wild individuals were collected by professional fishermen from two sites, in Tholi (THL) and Messolonghi (MES) on 25–29 November 2017. Wild fish were obtained from local fishermen shortly after capture (approximately 1 h), in the early morning, and were placed in plastic buckets filled with ice upon collection. Aquaculture fish were immediately placed in foam containers with ice upon collection. In both cases, fish were retrieved already dead once boats arrived at the docks, placed in insulated polystyrene foam containers filled with ice, and transferred to the laboratory. Although the fish were already dead at the time of collection, the time between death and sample processing was minimized. In the laboratory, all samples were handled under sterile conditions and processed following the same protocol to minimize potential post-mortem microbial changes and ensure consistency across samples. Dissections were performed immediately after the specimens arrived at the laboratory. The intact digestive tract of each sample was aseptically separated from the abdominal cavity, and its external surface rinsed several times with sterile phosphate-buffered saline (PBS) (137 mM NaCl, 2.7 mM KCl, 4.3 mM Na_2_HPO_4_, and 1.47 mM KH_2_PO_4_, pH 7.4). The tissue was divided into two distinct parts: the first part extended from the esophagus to the stomach and the pyloric caeca (upper digestive tract, UpD), and the second part included the anterior and posterior intestine, and the hindgut (lower digestive tract, LowD) (Supplementary Figure S2). Both the digesta-associated (transient) and the mucosal (adherent) microbiota were included in the analysis. Tissues were collected in sterile 1.5 mL microcentrifuge tubes containing RNA stabilizing reagent (fix RNA, EURX, Gdańsk, Poland) and stored in −20 °C until further processing. DNA extractions for 16S rRNA sequencing were completed within the following four days. Twenty individuals were dissected from each sampling area (Supplementary Table S1). Some samples were excluded from the NGS analysis due to a low number of reads. For the culture-dependent approach, tissues were placed in sterile 50 mL Falcon tubes and used to prepare homogenates. In this case, the gastrointestinal tract of three fish (150 ± 5 g) from each sampling area was used (Supplementary Table S1).

#### Study Site Characteristics

The aquaculture facilities represented contrasting environmental conditions. The Vonitsa site is located in the Ambracian Gulf, a landlocked embayment characterized by limited exchange with the Ionian Sea, river inflows, and seasonal stratification with a brackish surface layer (28–32 ppt in autumn) overlying a hypoxic saline bottom layer [49]. The Astakos site is situated in the oligotrophic Ionian Sea with stable salinity (~38.7 ppt) and well-oxygenated conditions (7–8 mg L^−1^). Wild fish were collected from two sites: Tholi in the open Ionian Sea, and the Klisova lagoon near Messolonghi, a shallow hypersaline coastal lagoon (40.6–55 ppt) with distinct benthic communities dominated by polychaetes and crustaceans [50].

### 2.2. Gastrointestinal Tissue DNA Extraction, PCR Amplification and Purification

DNA was extracted using the Stool DNA isolation kit (Norgen Biotek, Thorold, ON, Canada) following the manufacturer’s instructions. The total concentration and quality were determined by a Q5000 micro-volume spectrophotometer (Quawell, San Jose, CA, USA). The extracted DNA was stored at −20 °C until it was used for PCR amplification. A 460 bp fragment spanning the V3–V4 region of the 16S rRNA gene was amplified using the universal primer set 341F (5′-CCTACGGGNGGCWGCAG-3′) and 805R (5′-GACTACHVGGGTATCTAATCC-3′) [51]. Amplification was performed using the KAPA Taq PCR kit (Roche, Basel, Switzerland). Each reaction (25 µL) contained 2.5 μL of KAPA Taq Buffer (10×), 0.7 μL of dNTPs mix (10 mM each), 1 µL of each primer solution (10 µM), 0.3 µL of KAPA Taq DNA Polymerase (5 U/µL), 1 µL from the template DNA solution (≤250 ng) and 18.5 µL of sterile deionized water (SDW). The PCR amplification protocol included an initial denaturation step at 95 °C for 3 min, followed by 30 cycles of denaturation at 98 °C for 20 s, annealing at 60 °C for 15 s, and extension at 72 °C for 45 s. The reaction was terminated with a final extension step at 72 °C for 1 min. For each set of PCR reactions negative and positive controls were also included. The final elongation step was at 72 °C for 1 min. The amplification products were electrophorized on 1.5% *w*/*v* agarose gels and visualized in Bio-Rad’s Gel Doc™ XR+ system (Bio-Rad, Hercules, CA, USA). PCR products were purified with polyethylene glycol (PEG) precipitation [52]. Briefly, the reactions were mixed with an equal volume of a 20% PEG, 2.5 M NaCl solution, centrifuged at 14,000× *g* for 20 min and the precipitate was washed twice with 125 µL of 70% ethanol and centrifuged at 14,000× *g* for 10 min. The dried precipitates were suspended in 15 µL of sterile deionized water (SDW) and the concentration was measured with a Quawell Q5000 micro-volume spectrophotometer (Quawell, San Jose, CA, USA).

### 2.3. Illumina Library Preparation and Sequencing

Library preparation began with the incorporation of the Illumina barcodes and indexes with a second PCR step. Each sample was tagged with a unique combination of index primers to ensure successful demultiplexing. PCR amplification was performed using the KAPA Taq PCR Kit (Roche, Basel, Switzerland) in 50 μL reactions. Each reaction contained 10 µL of KAPA Taq Buffer (10×), 1.5 µL of dNTPs solution (10 mM each), 5 µL of the forward index primer (10 µM), 5 µL of the reverse index primer (10 µM), 1 µL of KAPA Taq DNA Polymerase (5 U/µL), 2 µL from the purified PCR product (10 ng/µL), and 25.5 µL of sterile deionized water (SDW). The cycling conditions included an initial denaturation step at 95 °C for 3 min, followed by 8 cycles of denaturation at 95 °C for 30 s, annealing at 55 °C for 30 s, and extension at 72 °C for 45 s. The termination was performed with a final extension step at 72 °C for 5 min. The amplicons were purified using the NucleoMag^®^ NGS Clean-up and Size Selection kit (Macherey-Nagel, Düren, Germany) following the manufacturer’s recommendations. Purified samples were suspended in 30 µL of sterile deionized water (SDW) and their concentration was measured with a Quawell Q5000 microvolume spectrophotometer (Quawell, San Jose, CA, USA). Samples were diluted to a final concentration of 8 nM and mixed equimolarly to create the sequencing library. The library was sequenced on an Illumina MiSeq sequencing platform by Macrogen (Seoul, Republic of Korea).

### 2.4. Data Analysis and Statistics

The demultiplexing of sequencing reads, adapter removal and conversion to FASTQ files were performed using bcl2fastq2 v2.20 (Illumina, San Diego, CA, USA). Analysis of reads was conducted using USEARCH v11 [53]. Paired-end reads were assembled, trimmed, and corrected using the fastq_mergepairs and the fastq_filter commands. Reads with less than 400 bp were excluded in this step. The fastx_uniques option was used to reduce redundancy by merging identical sequences into single, unique representatives. Sequences were clustered into operational taxonomic units (OTUs) using the cluster_otus command. Clustering was performed using the UPARSE-OTU algorithm with 97% identity threshold [54]. Chimeric sequences and singletons were also removed during this step. “Cross-talk” errors were filtered using the UNCROSS2 algorithm [55]. OTUs with less than 0.1% relative abundance across all the samples were discarded with the otutab_trim command. Taxonomy was assigned to OTUs using QIIME 2 release 2024.10 [56] and the built-in version of BLAST+ algorithm v2.16.0 [57]. The SILVA 16S rRNA gene database (release 138.2) was used as a reference database [58]. All the sequences assigned to eukaryotes (chloroplasts and mitochondria) were discarded from the analysis.

The OTUs count table was used to estimate Good’s coverage [59]. Alpha and beta diversity metrics were calculated using the R package vegan v2.7-1 [60]. The Simpson and Shannon diversity indices and the number of observed OTUs were included in the analysis. Significant differences in alpha diversity indices between groups were assessed using pairwise ANOVA. Beta diversity analysis to evaluate the structure of bacterial communities was based on Bray–Curtis dissimilarity. Beta diversity was visualized using Principal Coordinates Analysis (PCoA). Statistically significant differences between sample groups were identified with permutational multivariate analysis of variance (PERMANOVA) based on 999 permutations. *p*-values ≤ 0.05 were considered indicative of statistical significance in comparisons between groups. The Bonferroni-Hochberg method was used to adjust *p*-values for multiple PERMANOVA testing. Taxa were considered members of the core microbiome if they were present in at least 75% of the samples within a group and had at least 0.01% relative abundance in that group. To identify differentially abundant taxa between groups, the nonparametric Kruskal–Wallis rank sum test was used for multiple-group comparisons, followed by pairwise Wilcoxon rank-sum tests, as implemented in the R package microeco [61]. MetaXplore v1.0 (http://metaxplore.eu/, accessed on 19 May 2025) was used to perform statistical tests and visualize results with PCoA plots, heat maps and Venn diagrams [62]. Networks describing interactions within bacterial communities were constructed using the CoNet app v1.1.1 [63] in Cytoscape v3.10.3 [64]. All samples included in the NGS analysis were also used for the development of networks (67 wild and 74 aquaculture samples). Networks were built using Pearson and Spearman correlations, Kullback–Leibler and Bray–Curtis dissimilarities, and mutual information to calculate similarities. A combination of permutation and bootstrap distributions was used to calculate *p*-values for edges. Brown’s method was used to merge *p*-values, and Benjamini–Hochberg was selected as the correction method. Networks were visualized with Gephi v0.10 [65] using the ForceAtlas2 layout [66]. The default layout parameters were used except for gravity that was set at 10 to reduce the dispersion of disconnected nodes.

### 2.5. Isolation and Enumeration of Culturable Intestinal Microbiota

Each upper or lower digestive tract tissue sample was homogenized separately in sterile 50 mL Falcon tubes containing 5 mL sterile phosphate-buffered saline (PBS) using tissue grinders. Three homogenates were prepared for each tissue part and each area (Supplementary Table S1). The homogenates were incubated for 3–4 h at 37 °C with gentle shaking (~50 rpm). Tenfold serial dilutions (10^−1^ to 10^−6^) were prepared in sterile 1× PBS for each homogenate and 0.1 mL were spread on two solid nutrient media: TSA supplemented with 1% NaCl (1.5% *w*/*v* tryptone, 0.5% *w*/*v* soytone, 1.5% *w*/*v* NaCl and 1.5% *w*/*v* agar) and LB agar (1% *w*/*v* peptone, 1% *w*/*v* NaCl, 0.5% *w*/*v* yeast extract and 1.5% *w*/*v* agar). Plates were incubated for up to 48 h at 25 °C and 37 °C. Approximately 70 morphologically distinct colonies were selected per sample group and purified by re-streaking at least three times on the appropriate medium.

### 2.6. Strain Identification and Phylogenetic Analysis

The characterization of bacterial strains was based on the amplification of the 16S rRNA gene by colony PCR [67] using primers 27F 5′-AGAGTTTGATCCTGGCTCAG-3′ and 1492R 5′-GGTTACCTTGTTACGACTT-3′ [68]. PCR reactions with a total volume of 25 μL were prepared with the KAPA Taq PCR kit (Roche, Basel, Switzerland). They contained 1× KAPA Taq Buffer (10×), 0.2 mM of each dNTP, 0.4 μM of each primer, 0.5 U/µL of KAPA Taq, the appropriate volume of PCR-grade water, and a colony from a plate with a pure culture as the template. The cycling protocol included an initial step at 95 °C for 5 min, followed by 35 cycles of denaturation at 95 °C for 30 s, primer annealing at 54 °C for 30 s, and chain extension at 72 °C for 10 min. The reactions were completed with a final extension step at 72 °C for 10 min. PCR products were precipitated using polyethylene glycol (PEG), as described previously [52]. Sequencing reactions were prepared using purified PCR products and the ABI Big Dye Terminator v3.1 Cycle Sequencing Kit following the manufacturer’s instructions (Applied Biosystems, Thermo Fisher Scientific, Waltham, MA, USA). Two sequencing reactions were prepared for each amplicon using primers 518F 5′-CCAGCAGCCGCGGTAATACG-3′ and 800R 5′-TACCAGGGTATCTAATCC-3′, respectively [69,70]. Sequencing reactions were purified according to the manufacturer’s recommendations and sequenced on an ABI 3730xl DNA Analyzer (Applied Biosystems, Thermo Fisher Scientific, Waltham, MA, USA). Chromatograms were assembled and trimmed using Geneious v7.0.6 [71]. Closely related reference sequences were identified by MegaBLAST searches against the core nucleotide dataset (core_nt), and the 16S Ribosomal RNA (Bacteria and Archaea) RefSeq dataset using the BLAST+ v2.16.0 suite [57,72]. Phylogenetic analyses, including multiple alignments and tree construction, were carried out using Geneious v7.0.6 [71]. Multiple alignments were performed using the implemented in Geneious version of MUSCLE v3.8 [73]. The phylogenetic tree was constructed using the neighbor-joining method and resampled 1000 times with the bootstrap method. The Tamura-Nei genetic distance model was used to calculate distances between sequences. The tree was visualized with the iTOL v5 online tool [74]. The reported percentages correspond to the proportion of colonies recovered for each taxon, rather than depicting the in situ community structure or the relative abundance of this taxon in the community.

## 3. Results

### 3.1. Amplicon Sequencing Dataset and General Community Structure

A total of 141 gastrointestinal samples of *S. aurata* were sequenced via 16S rRNA gene amplicon analysis, producing 1,756,320 high-quality filtered reads. On average, 12,456 reads were divided into each sample. The Good’s coverage was calculated between 0.93 and 1.00 for each sample, indicating that the communities were well described by the identified bacterial taxa. Filtered reads were clustered into 117 operational taxonomic units (OTUs) which displayed at least 0.1% relative abundance. The OTUs were grouped into nine unique phyla, eleven classes, 25 orders, 48 families and 77 genera (Supplementary Table S2).

Approximately 98% (RA%: Percentage relative abundance) of the sequences were assigned to five dominant bacterial phyla: Pseudomonadota (53%), Bacillota (29%), Actinomycetota (7%), Deinococcota (5%), and Bacteroidota (4%) (Supplementary Table S3). Representatives of four additional phyla with equal or less than 1% relative abundance were also identified. At the class level, Gammaproteobacteria dominated the dataset, representing approximately 50% of all classified sequences, followed by Bacilli (28%), Actinobacteria (7%), Deinococci (5%), Bacteroidia (4%), and Alphaproteobacteria (3%).

**Figure 1:**
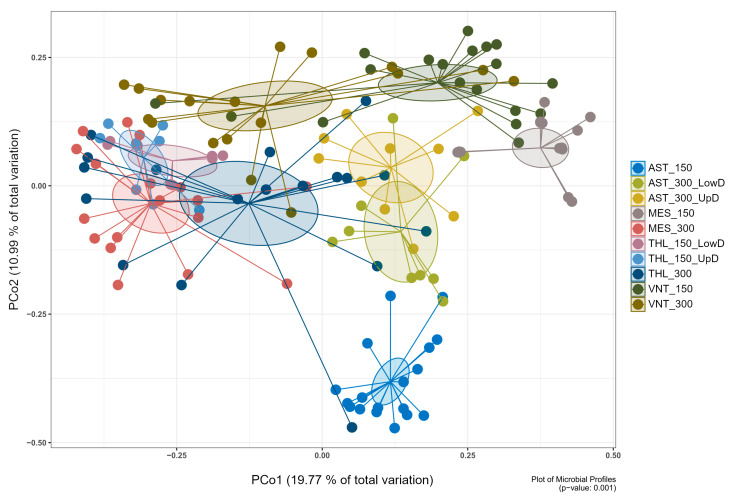
Principal coordinate analysis (PCoA) ordination plot of bacterial communities associated with the gastrointestinal tissue of *S. aurata* based on Bray–Curtis dissimilarity. The first two principal coordinates explained 19.77% and 10.99% of the variation, respectively. The plot contains ten sample groups originating from two aquaculture facilities in Astakos (AST) and Vonitsa (VNT), and two wild fisheries in Messolonghi (MES) and Tholi (THL). Fish were sampled at two growth phases corresponding to body weights of 150 ± 5 g (150) and 300 ± 5 g (300). The gut tissue was divided into upper digestive (UpD; esophagus, stomach, pyloric caeca) and lower digestive tract (LowD; midgut, hindgut). PERMANOVA confirmed significant differences between all groups (*p* ≤ 0.05; see Supplementary Table S4 for pairwise comparisons).

Principal coordinate analysis (PCoA) demonstrated clear separation of microbial communities based on origin (wild vs. aquaculture), population, and body weight (150 g vs. 300 g) (*p* ≤ 0.05) (Supplementary Table S4). At the lowest level, samples were divided into sixteen groups based on their attributes, specifically the collection area, body weight, and tissue part. Pairwise comparisons of different tissue parts within the same area and body weight group revealed several similarities in bacterial community structure (*p* > 0.05) (Supplementary Table S4). As a result, twelve groups were consolidated into six, while four remained separate. Pairwise comparisons of the ten resulting groups revealed significant differences in the structure of the microbiota (*p* ≤ 0.05) (Figure 1; Supplementary Figure S3), therefore the groups remained separate, and subsequent analysis was based on them. Regarding alpha diversity metrics, wild fish from Messolonghi and Tholi were characterized overall by higher species diversity and evenness compared to aquaculture fish (Supplementary Figure S4; Supplementary Table S5). However, certain variability was observed even in that case, with 150 g fish from Messolonghi (MES_150) displaying relatively low diversity values.

### 3.2. Microbial Composition Across Samples and Core Taxa

At the phylum level, the gastrointestinal tract of wild and aquaculture fish was dominated by Pseudomonadota, comprising 59% and 48% of the communities, respectively, with this difference being statistically significant (*p* ≤ 0.05) (Supplementary Figure S5). Actinomycetota, Deinococcota, Spirochaetota, and Fusobacteriota had a stronger presence in the gut tissue of aquaculture fish (*p* ≤ 0.05) while Bacillota, the second most abundant phylum, was present with similar relative abundance in both categories. Similar trends were observed in the relative abundance of phyla across the sample groups, with Pseudomonadota prevailing in eight groups (38–71%) and Bacillota in two (41–54%). Variation was mainly observed in the intestinal tract of aquaculture fish from Astakos with body weights of 300 g, where the bacterial community was more evenly distributed among three additional phyla: Actinomycetota, Deinococcota, and Bacteroidota.

**Figure 2:**
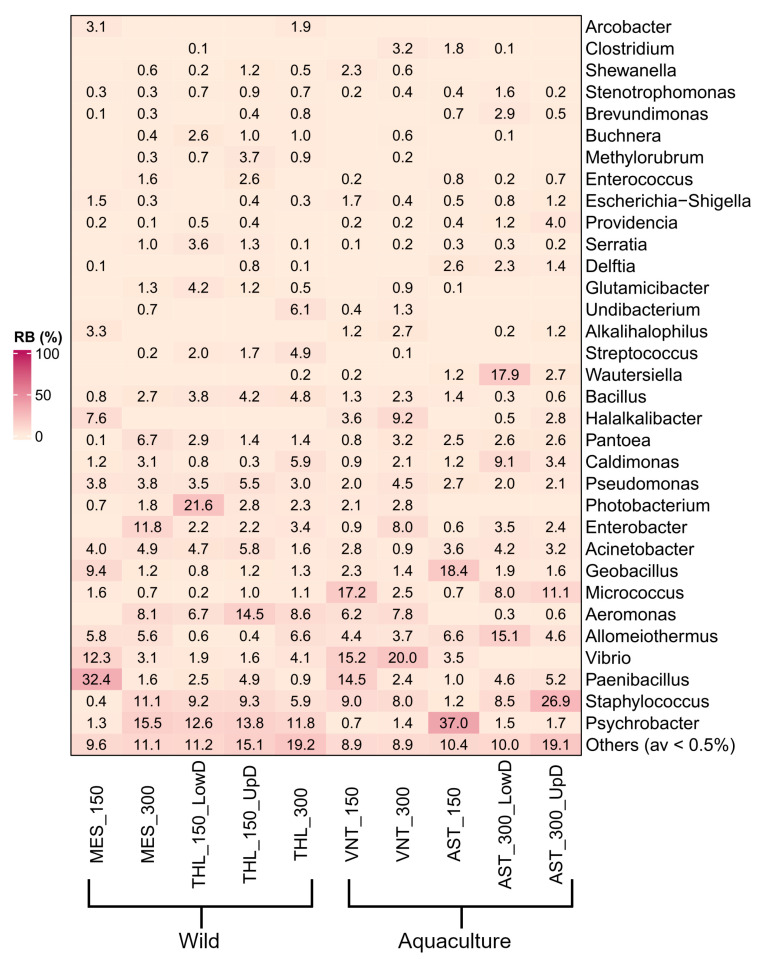
Heat map representing relative abundance (%) of dominant bacterial phyla and classes across wild and aquaculture *Sparus aurata* populations based on Illumina 16S rRNA gene sequencing.

*Psychrobacter* (9.7%), *Staphylococcus* (8.9%), *Paenibacillus* (7%), *Vibrio* (6.2%), *Allomeiothermus* (5.3%), and *Aeromonas* (5.2%) were overall the most abundant genera in the dataset (Figure 2). However, high variation was observed in the prevalent genus within individual samples. Most samples contained unique prevalent genera, including *Paenibacillus* (32.4% in MES_150), *Photobacterium* (21.6% in THL_150_LowD), *Aeromonas* (14.5% in THL_150_UpD), *Micrococcus* (17.2% in VNT_150), *Vibrio* (20% in VNT_300) *Wautersiella* (17.9% in AST_300_LowD), and *Staphylococcus* (26.9% in AST_300_UpD). Only *Psychrobacter* was prevalent in three samples, with varying relative abundances of 37%, 15.5%, and 11.8% in samples from Astakos (AST_150), Messolonghi (MES_300), and Tholi (THL_300), respectively. Multiple significant differences in relative abundance were observed between samples using a 0.5% abundance threshold (489 in total, *p* ≤ 0.05). Pairwise comparisons between the eight most abundant genera revealed 165 statistically significant differences across the samples (Supplementary Figure S6). Among these, *Paenibacillus* in MES_150 (32.4%) and *Psychrobacter* in AST_150 (37%) were the only genera with higher relative abundance compared to all other samples. AST_150 also contained the highest proportion of *Geobacillus* sequences (18.4%), which was comparable only to MES_150 (9.4%, *p* > 0.05). *Micrococcus* was enriched in VNT_150 (17.2%) and AST_300_UpD (11.1%) showing significant differences relative to seven samples, except AST_300_LowD (8%, *p* > 0.05). Other abundant genera, such as *Allomeiothermus* in AST_300_LowD (15.1%), *Aeromonas* in THL_150_UpD (14.5%), *Vibrio* in both samples from Vonitsa (20 and 15.2%, respectively), and *Staphylococcus* in AST_300_UpD (26.9%), displayed fewer significant differences among samples.

Interesting insights into the bacterial communities of *S. aurata* gastrointestinal tissue were acquired by examining sample groups. Comparison between wild and aquaculture-reared fish revealed clear differences in genus-level composition (Supplementary Figure S7). Wild fish harbored mainly *Psychrobacter* (10.8%), *Aeromonas* (7.1%), *Acinetobacter* (3.9%), *Photobacterium* (3.9%), and *Pseudomonas* (3.8%) (*p* ≤ 0.05). In contrast, aquaculture individuals showed a stronger presence of *Vibrio* (9.6%), *Allomeiothermus* (6.1%), *Micrococcus* (7.7%), *Geobacillus* (5.9%), and *Halalkalibacter* (3.6%) (*p* ≤ 0.05). Both groups contained similar quantities of *Staphylococcus*, *Paenibacillus*, and *Enterobacter* (*p* > 0.05), despite considerable variation among individual samples (Figure 2).

Based on the geographic origin of the studied populations, wild fish from Messolonghi harbored higher titers of *Paenibacillus* (15.6%), *Vibrio* (7.3%) and *Geobacillus* (4.9%), while fish from Tholi showed differential abundance in *Aeromonas* (9.8%) and *Photobacterium* (6.4%) (*p* ≤ 0.05) (Supplementary Figure S7). More differentially abundant genera were identified in aquaculture populations. The bacterial communities of fish from Astakos were distinguished from Vonitsa by the stronger presence of *Psychrobacter* (18.8%), *Staphylococcus* (9.9%), *Allomeiothermus* (8.1%), and *Geobacillus* (9.8%), and by the lower frequency of *Vibrio* (17.5%), *Aeromonas* (7%), *Halalkalibacter* (6.3%), and *Photobacterium* (2.5%).

The highest impact of body weight on the microbiota was recorded in wild fish from Messolonghi, with this population displaying the largest number of differentially abundant genera (21) between the two body weight categories (Supplementary Figure S7). In this case, 150 g fish were associated with high occurrence of *Paenibacillus* (32.4%), *Vibrio* (12.3%), and *Geobacillus* (9.4%), while *Psychrobacter* (15.5%), *Staphylococcus* (11.1%), and *Aeromonas* (8.1%) were detected in mature fish (*p* ≤ 0.05). Generally, weight related differences were more evident within collection sites than across them. However, few bacteria exhibited uniform trends across sample groups. For example, *Paenibacillus* seemed to be associated mostly with 150 g fish, since it was present with higher frequencies compared to 300 g fish not only in Messolonghi, but also in wild fish from Tholi (3.9%) and farmed fish from Vonitsa (14.5%). However, the opposite trend was observed for farmed fish from Astakos, with larger fish containing higher titers of the bacterium than smaller-sized fish (4.9% vs. 1%). *Enterobacter* was associated with 300 g fish in all populations. However, Tholi was the only population where the difference between younger and mature fish was not statistically significant.

Few bacterial genera exhibited tissue tropism. Among them, *Photobacterium* in 150 g fish from Tholi was associated with the lower part of the digestive tract (21.6% vs. 2.8%), and *Aeromonas* with the upper digestive tract (14.5% vs. 6.7%) (Figure 2). In 300 g fish from Astakos, *Allomeiothermus* (15.1% vs. 4.6%), *Caldimonas* (9.1% vs. 3.4%) and *Wautersiella* (17.9% vs. 2.7%) were more frequent in the lower digestive tract, whereas *Staphylococcus* (26.9% vs. 8.5%) in the upper digestive tract. Despite the observed differences in relative abundance for these genera, none of the above comparisons were statistically significant (*p* > 0.05).

**Figure 3:**
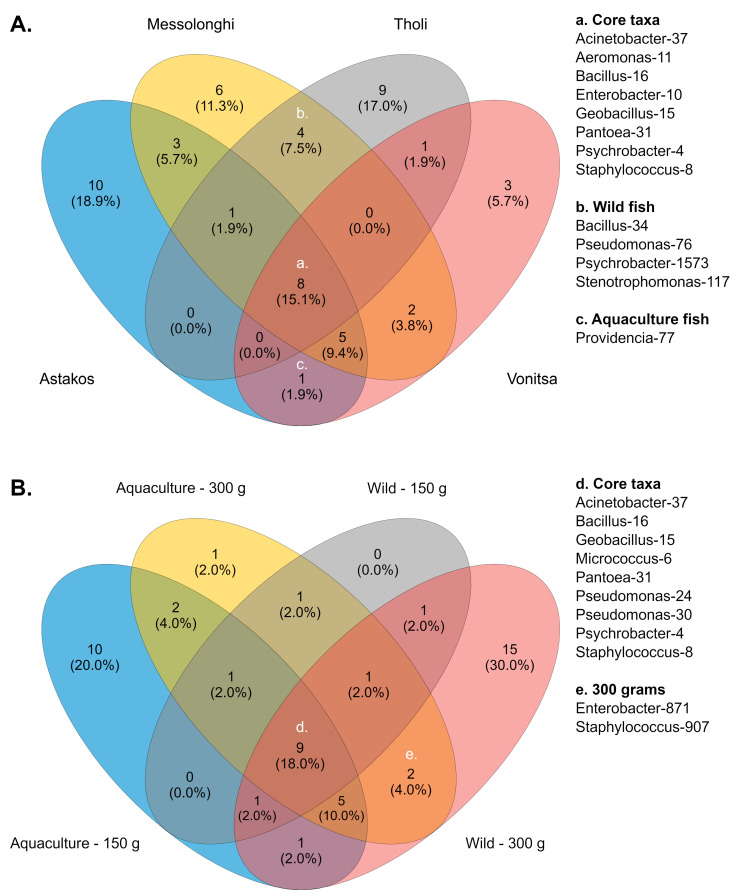
Core bacteria in (**A**) the studied populations and (**B**) different growth stages.

Across all studied populations, several bacterial genera were consistently detected, suggesting the presence of a putative core microbiome. These comprised prevalent taxa, such as *Psychrobacter* (otu4, 8.1%), *Staphylococcus* (otu8, 6.9%), *Geobacillus* (otu15, 4.6%), *Aeromonas* (otu11, 3.6%), *Enterobacter* (otu10, 2.8%), *Pantoea* (otu31, 2%), *Bacillus* (otu16, 1.7%), and *Acinetobacter* (otu37, 0.9%) (Figure 3A, Supplementary Table S6). Six groups (*Acinetobacter*, *Allomeiothermus*, *Caldimonas*, *Micrococcus*, and *Pseudomonas*) were detected in three populations. Different *Psychrobacter* (otu1573) and *Bacillus* (otu34) taxa, along with *Pseudomonas* (otu76) and *Stenotrophomonas* (otu117), were identified as core members of wild populations. In contrast, only *Providencia*-77 was common between the two aquaculture populations. Furthermore, each population exhibited a distinct number of unique bacterial groups—ten in Astakos, nine in Tholi, six in Messolonghi, and three in Vonitsa—reflecting variations in community composition across collection sites.

A similar set of core bacteria was detected between different sample groups when communities were examined according to fish growth stage. In this case, nine taxa were identified as members of the core microbiota (Figure 3B, Supplementary Table S7). Six taxa, *Psychrobacter* (otu4, 8.1%), *Staphylococcus* (otu8, 6.9%), *Geobacillus* (otu15, 4.6%), *Pantoea* (otu31, 2%), *Bacillus* (otu16, 1.7%), and *Acinetobacter* (otu37, 0.9%), had been previously identified as members of the core microbiome based on geographic origin. The remaining three members included *Micrococcus*-6, a frequent taxon with 4.5% relative abundance, and two *Pseudomonas* taxa (otu24 and 30), which were identified with low relative abundances (0.9% and 0.5%, respectively). Eight different taxa, including *Acinetobacter*, *Pseudomonas*, *Providencia*, *Enterobacter*, and *Aeromonas*, were common across three groups. *Delftia*-26 and *Paenibacillus*-20 were consistently present in both growth phases of aquaculture fish. Similarly, 150 g and 300 g wild fish shared *Psychrobacter*-1573. *Enterobacter*-871 and *Staphylococcus*-907 were the only core bacteria associated with a specific growth stage, as they were identified in both wild and aquaculture fish weighing 300 g. Most unique core bacteria were identified in two body weight groups: wild fish weighing 300 g and aquaculture fish weighing 150 g, which contained 15 and 10 taxa, respectively. Conversely, no unique core members were identified in wild fish weighing 150 g, and only *Ralstonia*-62 was associated with mature aquaculture fish (300 g).

### 3.3. Interactions Between Bacterial Taxa

**Figure 4:**
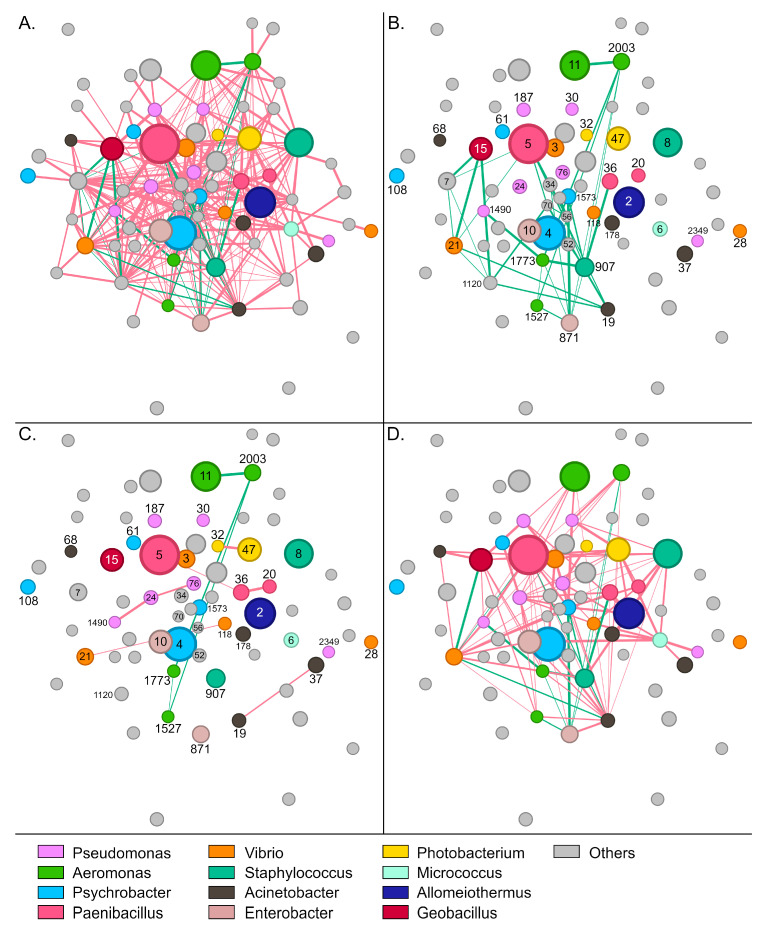
Network of interactions between bacterial groups in wild *S. aurata* gut samples. Nodes (circles) represent bacterial OTUs and edges (lines) interactions. Red colored edges depict mutual exclusions and green edges cases of copresence. (**A**) The complete network with 73 OTUs and 337 interactions. (**B**) Positive interactions between bacterial groups. (**C**) Interactions within abundant genera. (**D**) Interactions among different abundant genera. Numbers indicate the name of the specific OTU (node). For example, 5 refers to otu5 (Supplementary Table S2).

The gut bacterial community of wild *S. aurata* samples displayed 337 statistically significant interactions (edges) between 73 OTUs (nodes) (*p* < 0.05). Conversely, no significant interactions were recorded for the remaining 37 OTUs that were present in the community of wild samples. Most interactions were identified as mutual exclusions (302, 89.6%), and only 35 (10.4%) as copresence (Figure 4A,B).

*Paenibacillus*-5, which was the most abundant OTU in wild samples, developed 19 negative and six positive interactions with other members of the community. The bacterium was associated positively with *Acinetobacter*-19, *Halalkalibacter*-7, *Methylibium*-70, *Methylobacterium*-56, and *Vibrio*-21 (Supplementary Figure S8B). *Enterobacter*-871 and *Staphylococcus*-907 developed the highest number of positive interactions with other members of the community (Figure 4B). These bacteria were positively correlated together and each with six additional OTUs, including *Psychrobacter*-1573, *Aeromonas* (otus1527, 1773, and 2003), *Niallia*-52, *Bacillus*-34, and *Paenibacillus*-36. Similarly to *Paenibacillus*-5, *Alkalihalophilus*-1120 developed six positive interactions and was also part of this bacterial consortium that seems to stabilize the community.

Regarding the most abundant genera, a sparse network of mainly negative interactions was observed between OTUs of the same genus (Figure 4C). On several occasions, such as *Psychrobacter*, *Enterobacter*, and *Staphylococcus*, no interactions were recorded among OTUs. Interestingly, positive associations were only recorded between the four *Aeromonas* OTUs, with otu2003 forming positive interactions with all the other *Aeromonas* nodes. A much denser network was observed for interactions across different genera (Figure 4D). Although mutual exclusions were prevalent, *Vibrio*-21 associated positively with *Geobacillus*-15 and *Acinetobacter*-19, whereas *Aeromonas*-1773 correlated with *Pseudomonas*-1490, *Enterobacter*-871 and *Staphylococcus*-907. Certain groups, like *Staphylococcus*-8 and *Photobacterium*-47, developed exclusively negative interactions—19 and 21, respectively—with the other abundant members of the community.

**Figure 5:**
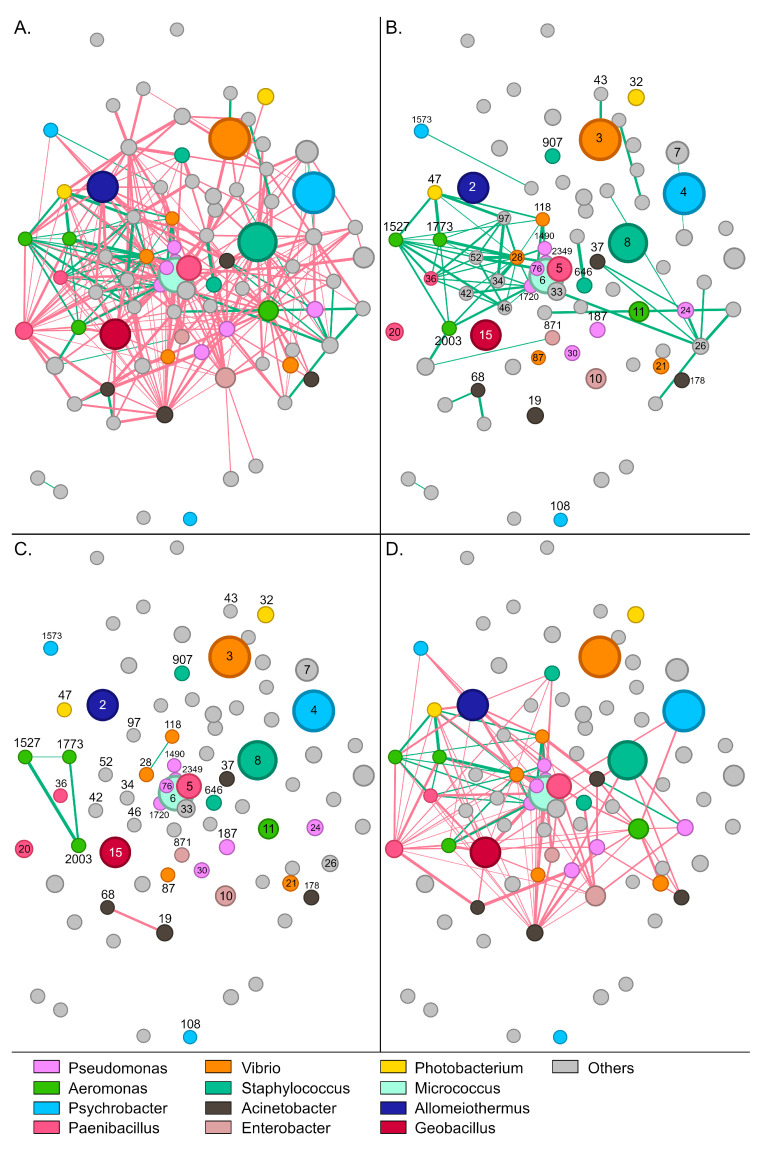
Association network between bacterial groups in farmed *S. aurata* gut samples. Nodes (circles) represent bacterial OTUs and edges (lines) interactions. Red colored edges depict mutual exclusions and green edges cases of copresence. (**A**) The complete network with 83 OTUs and 312 interactions. (**B**) Positive interactions between bacterial groups. (**C**) Interactions within abundant genera. (**D**) Interactions among different abundant genera. Numbers indicate the name of the specific OTU (node). For example, 5 refers to otu5 (Supplementary Table S2).

Eighty-three members of the bacterial community in aquaculture samples formed a network of 312 significant interactions (*p* < 0.05). The remaining 26 OTUs identified in the gut tissue of aquaculture fish did not form any significant interactions with the rest of the community. Similarly to the wild populations, most of the interactions were identified as mutual exclusions (250, 80.1%), whereas cases of copresence accounted for the remaining 19.9% (62 interactions) (Figure 5A,B).

In the microbiota of aquaculture fish, the most abundant bacteria, *Psychrobacter*-4 and *Vibrio*-3 developed a rather thin network with only one positive and eight negative associations (Supplementary Figure S8D). The positive interaction was developed between *Vibrio*-3 and *Cloacibacterium*-43. On the contrary, *Psychrobacter*-4 disassociated with prevalent members of the community, such as *Paenibacillus*-5, *Aeromonas*-11, and *Halalkalibacter*-7. Compared to wild fish, the community of aquaculture fish was characterized by a denser network of positive interactions (Figure 5B). The network was separated into two smaller independent subnetworks. At the center of the first subnetwork, *Vibrio*-28 associated positively with fourteen members of the community, including *Aeromonas* (otu1527, 1773, 2003), *Bacillus*-34, *Buchnera*-46, *Glutamicibacter*-42, *Niallia*-52, *Paenibacillus*-36, *Photobacterium*-47, *Pseudomonas* (otu1490, 1720, 2349), *Rothia*-97, and *Vibrio*-118. On the other side of the network, *Delftia*-26 developed a smaller subnetwork of positive interactions with seven members of the community, including *Acinetobacter*-37, and *Pseudomonas*-24. The subnetworks remain independent mostly due to the presence of *Wautersiella*-33 that associates with *Delftia*-26 but interacts negatively with *Vibrio*-28, and *Aeromonas*-1773. A high number of positive interactions was also recorded for *Aeromonas*-1527 (nine interactions), and *Aeromonas*-1773, *Niallia*-52, and *Paenibacillus*-36 which displayed seven cases of copresence with other bacteria.

Only five significant interactions were recorded between OTUs of the same genus for the most abundant genera of the gut microbiota (Figure 5C). They were observed among members of *Acinetobacter*, *Aeromonas*, and *Vibrio*. Similarly to what was observed in wild samples, three *Aeromonas* OTUs associated together by forming a closed network (1527, 1773, and 2003). However, in this case, *Aeromonas*-otu11 was excluded from this positive correlation. As expected, interactions between OTUs that belong to different genera were much more abundant (Figure 5D). Most of the interactions were identified as mutual exclusions (92 interactions) and only twenty as copresence. For example, *Micrococcus*-6 and *Acinetobacter*-19 formed 10 and 18 negative interactions, respectively. Moreover, bacteria that formed several positive interactions with other abundant genera in wild samples, like *Enterobacter*-871 and *Paenibacillus*-5, were mostly involved in negative interactions in aquaculture samples.

### 3.4. Analysis of the Culturable Bacterial Diversity

**Figure 6:**
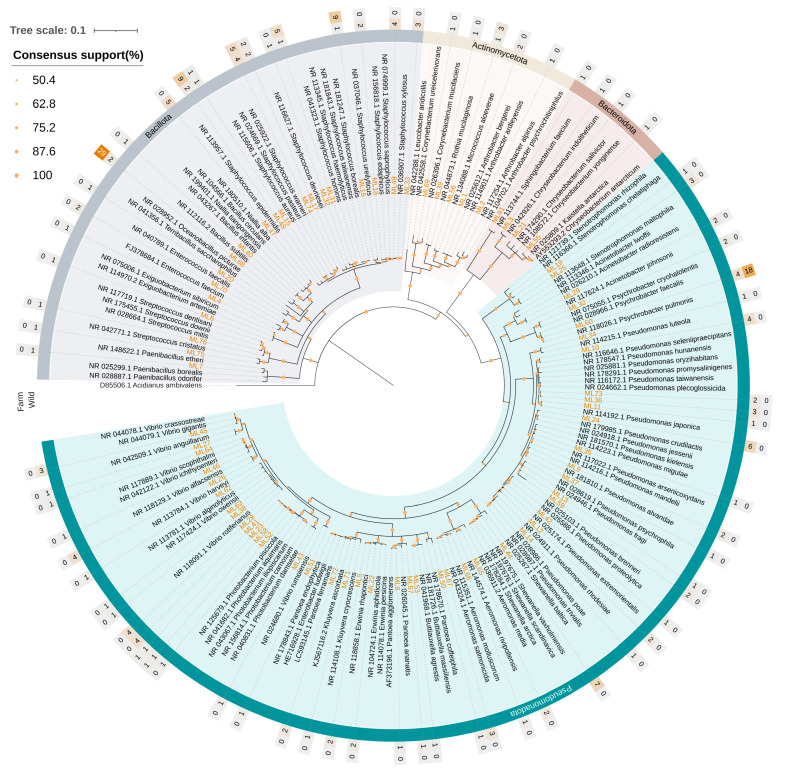
Neighbor-joining phylogenetic tree based on 16S rRNA gene sequences (~1000 bp) of 220 bacterial strains isolated from the gut tissue of *S*. *aurata*, showing evolutionary relationships among the identified taxa. Bootstrap support values (1000 replicates) are shown as circles at nodes; branches with <50% support were collapsed. The Tamura-Nei genetic distance model was used for tree construction. The 16S rRNA sequence of *Acidianus ambivalens* (Crenarchaeota) served as the outgroup. The heat map rings indicate the number of isolates identified for each bacterial species in wild (inner ring) and aquaculture fish (outer ring). Branch colors indicate the phylum: green = Pseudomonadota, grey = Bacillota, light sand yellow = Actinomycetota, brown = Bacteroidota.

The phylogenetic analysis was based on 220 isolated strains, most of which originated from wild samples (101 from Messolonghi and 36 from Tholi). The remaining 83 strains were isolated from aquaculture fish, with 46 originating from Vonitsa and 37 from Astakos. The taxonomic placement of the 16S rRNA sequences (≥1000 bp) revealed mostly similarities with the NGS approach, with a certain variation in frequencies (Figure 6). The most abundant phylum was Pseudomonadota (50.0%), followed by Bacillota (44.1%) Actinomycetota (4.1%), and Bacteroidota (1.8%). Contrary to the high-throughput sequencing approach, no representatives of the Deinococcota were identified.

The composition of the communities at lower taxonomic levels revealed striking differences between wild and aquaculture fish. The culturable gut community of wild samples was far richer compared to farmed fish, containing representatives from all the phyla (Supplementary Figure S9). In total, strains belonging to 28 genera were identified in wild samples compared to only five in farmed fish (Supplementary Table S8). *Pseudomonas* was the most frequently observed genus in wild fish and *Staphylococcus* in aquaculture samples, with 20.4% and 37.3% occurrence, respectively. Additionally, *Staphylococcus* (15.3%) and *Vibrio* (13.1%) were recorded with high relative abundance values in wild fish, while *Bacillus* (34.9%) and *Psychrobacter* (21.7%) were the other two prevalent genera in the aquaculture microbiota.

At the species level, aquaculture fish were mainly characterized by the presence of *Bacillus subtilis*, which was identified with high relative abundance in both areas and both parts of the digestive tract (Supplementary Figure S9). In fish from Astakos, the bacterium was dominant with 45.8% and 69.2% in the upper and lower digestive tract, respectively. High occurrence was also observed for other Bacillota species, specifically for members of the genus *Staphylococcus*. In this regard, *Staphylococcus ureilyticus* was the dominant species isolated from the upper digestive tract of fish from Vonitsa (33.3%). On the other hand, most of the strains isolated from the lower digestive tract of fish from Vonitsa were identified as *Psychrobacter pulmonis* (47.4%). The remaining two bacterial species found in aquaculture fish, *Micrococcus aloeverae* and *Photobacterium carnosum*, were isolated only from Vonitsa and Astakos, respectively.

In the case of wild populations, the higher microbial diversity was reflected in the presence of twelve *Pseudomonas*, nine *Staphylococcus*, and nine *Vibrio* species (Supplementary Figure S9). Most *Pseudomonas* strains were associated with the upper digestive tract of fish from Messolonghi, although occurrences were also recorded in other samples. Among them, *P. crudilactis* displayed the highest frequency (9.1%) in the upper digestive tract of fish from Messolonghi. The lower digestive tract of fish from the same area contained *P. promysalinigenes*, and *Shewanella vaxholmensis*, both with 6.5%. Conversely, in Tholi, *Niallia alba* was the dominant species in the upper digestive tract (9.5%), whereas the lower digestive tract was strongly associated with *Staphylococcus xylosus* and *Vibrio alfacsensis*, which were the most frequently isolated species with 20% occurrence. Different *Vibrio* species were recorded between the two areas. *Vibrio alfacsensis*, *V*. *anguillarum*, and *V*. *rotiferianus* were isolated from Tholi, whereas *V*. *ichthyoenteri*, *V*. *owensii*, *V*. *rumoiensis*, and *V*. *scophthalmi* were found only in fish caught in Messolonghi. *Vibrio gigantis* was mostly associated with fish from Tholi (5.8% average in both tissues), although one strain was also isolated from the lower digestive tract of fish from Messolonghi. Notably, *Vibrio harveyi* was present in the upper digestive tract of samples from both areas with similar percentage, 5.5% in Messolonghi and 4.8% in Tholi. Additionally, in both areas, *Staphylococcus* species were mostly associated with the upper part of the digestive tract, despite the strong presence of *Staphylococcus xylosus* in the lower digestive tract of fish from Tholi (20%). Regarding other bacterial taxa, three *Aeromonas* species were found exclusively in the lower digestive tract of fish in both collection sites, and four *Pantoea* species were isolated only from fish in Messolonghi.

## 4. Discussion

The study provides a detailed comparison of the gut microbiota in gilthead seabream (*Sparus aurata*) between wild and aquaculture populations, using 16S rRNA gene sequencing to characterize bacterial community structure across different habitats, growth phases, and parts of the gastrointestinal tract. The high-throughput sequencing approach was coupled with cultivation techniques to explore the culturable diversity of the intestinal tract and isolate members that could be tested for putative probiotic activity. The main findings suggest that rearing practices, geographic location, and host body weight are key determinants of gut microbial diversity and composition in the studied *S. aurata* populations. Conversely, a weaker effect was exerted by the different microhabitats of the intestinal tract.

### 4.1. The General Community Structure in Wild and Aquaculture Samples Shows Differences Driven by Multiple Factors

Analysis of alpha diversity metrics revealed overall greater microbial diversity in wild populations. A similar trend has been previously recorded in gilthead sea bream [75,76]. This suggests that in the case of *S. aurata*, natural habitats, with more complex and variable environmental inputs, promote richer and more evenly distributed bacterial communities. Higher diversity is often associated with ecosystem stability and greater resilience to environmental stress and disease [11,77,78,79]. In contrast, aquaculture fish showed lower diversity, consistent with previous studies indicating that intensive farming practices, including vaccination, high stocking densities, disinfection, and antibiotics, as well as uniform diets lead to more simplified gut microbiota [11,77]. This difference in diversity was even more evident in the culture-based approach, with only five bacterial genera identified in farmed fish compared to 28 genera in wild samples.

Beta diversity analysis confirmed structural differences, indicating significant dissimilarity between groups based on origin (wild or aquaculture fish), location, and growth stage. With respect to origin, previous studies identified considerable differentiation in the gut microbiota of wild and farmed gilthead seabream [75,76], as well as other fish species [23]. In this context, several factors may be responsible for shaping the composition of the gut microbial community, including diet, management, environmental conditions, or host genetic background [11,14,80]. For example, diet is generally considered a strong determinant of the fish microbiome [14,15,81]. Specifically in farmed *S. aurata*, altered synthetic diets with probiotics or additives significantly modified the resident gut microbiota [21,27,32,82,83], or both the resident and transient microbiotas [29,30,34,84,85]. Diet also had a stronger effect than the aquatic medium on the microbiota of *S. aurata* larvae reared under controlled hatchery conditions [86]. These dietary shifts are also known to influence digestive processes, as both host-derived and microbial enzymes respond dynamically to changes in nutrient availability. Such interactions highlight how nutritional factors can modulate the structure of the gut microbiota as well as the functional potential in the gastrointestinal tissue of fish [87]. On the contrary, a reduced effect of diet on the resident gut microbiome of farmed *S. aurata* was also observed [23,31]. In Ruiz et al. [23], a shift to a wild-type diet resulted in temporary increase in microbial diversity which diminished over time and eventually led to the formation of similar indigenous communities to the commercial diet. Additionally, the genetic profile was a major driver of the differences observed in the microbiota of farmed gilthead sea bream, between a reference and a genetically modified population throughout development [35]. In our study, the two experimental groups were reared under substantially different feeding regimes (synthetic vs. natural diet) and management practices, including handling, vaccination, disinfection, putative use of antibiotics, and population density. The aquaculture fish originated from selectively bred broodstock optimized for growth performance and disease resistance, resulting in genetic profiles distinct from wild populations. Previous research has demonstrated that host genetics can significantly influence gut microbiota composition in gilthead sea bream [35], suggesting that genetic differences between our experimental groups may have contributed to the observed microbiota variation. Furthermore, the collected fish lived in different environmental conditions, in terms of water chemistry and quality. For these reasons, it is difficult to evaluate the exact contribution of each parameter to the variation observed between the studied wild and aquaculture fish.

### 4.2. Bacterial Communities of Different Populations Are Affected by Geographic and Environmental Factors

Grouping samples based on geographic location offered deeper insight into the factors that influenced community structure. As shown by the analysis, the four studied populations formed diverse bacterial profiles, suggesting a strong impact of geography. The effect of geography on the microbiota of fish seems to be associated with environmental parameters (e.g., habitat, diet, temperature, salinity, etc.) or host phylogeny and genetics, and even though it has not been entirely elucidated, there are several studies that have identified changes based on it [8,81,88,89,90]. In gilthead seabream, previous work on different Greek populations, observed no significant effect of geography on the structure of the microbiota, with high inter-individual variability in specific bacterial taxa between collection sites [22]. Notably, the gut bacterial communities of farmed populations in Astakos and Vonitsa exhibited distinct bacterial profiles despite similar host genetic backgrounds, and the use of identical management and feeding practices at both facilities. This suggests that the local environment may be responsible for the observed variation, even under standardized aquaculture conditions. Indeed, the sea cages in Vonitsa are located in the Ambracian (or Amvrakikos) gulf, a landlocked area with limited exchange with the open sea (the Ionian Sea), river inflows, and mesotrophic or seasonally eutrophic water [49,91]. The water is stratified into a top brackish layer with variable salinity (11–22 ppt in winter and 31–33 in summer), where aquaculture cages are located, and a hypoxic/anoxic saline bottom layer (36–39 ppt). In October-November, salinity in the top layer may range from 28 to 32 ppt, temperature from 19 to 22 °C, and dissolved oxygen from 6 to 8 mgL^−1^ [49,92,93]. On the contrary, the sea cages in Astakos are situated in the oligotrophic Ionian Sea which is characterized by average salinity of 38.7 ppt, water temperature ranging from 19 to 23 °C (https://marine.copernicus.eu/, accessed on October 2025), and dissolved oxygen from 7 to 8 mgL^−1^ during the same period [94,95,96]. Therefore, nutrient levels in water, salinity, and dissolved oxygen could be regarded as putative factors contributing to the observed variation in bacterial community structure between the two aquaculture sites. However, the lack of direct measurements during sample collection prevents a more detailed evaluation of their effect. Oxygen availability can have a profound effect on the structure and composition of the intestinal microbiota as previously observed in *S. aurata* [97] and several other marine organisms [98,99,100]. The same has been reported for salinity, especially since the fish intestine is involved in osmoregulation [8,14,17,18].

Local environmental conditions could also be regarded as putative reasons for the differences observed in the microbiota of wild populations in Messolonghi and Tholi, despite the proximity of both fishing sites. Similarly to the aquaculture populations, the fishery in Tholi is situated in the Ionian Sea and the site in Messolonghi in the isolated shallow coastal lagoon of Klisova. Apart from the small depth (1 m mean depth), the lagoon is also characterized by hypersaline water (40.6 ppt in autumn and 55 ppt in summer), with temperature ranging from 14.4 to 15.9 °C, and dissolved oxygen from 7.6 to 10.2 mgL^−1^ in autumn [50,101]. As in the case of the aquaculture populations, the absence of direct measurements does not allow for a more comprehensive analysis of the influence of these environmental parameters. Additionally, in the case of wild populations, the influence of diet cannot be entirely excluded due to the variable and uncertain nature of natural feed. In this context, the lagoon exhibited less diverse benthic macrofaunal communities than the Ionian Sea, consisting primarily of polychaetes and crustaceans, such as *Microdeutopus* sp. [50,102,103].

### 4.3. The Effect of Body Growth Phase and Tissue Localization

Within each habitat, fish of different body weight categories (150 g and 300 g) harbored distinct intestinal bacterial communities. Changes in the gut microbiota during development have been recorded in multiple fish species, and have been associated with the microbiota of the surrounding water, host genotype and innate immune responses, vertical transmission, dietary habits, or changes in physiological functions, such as osmoregulation [11,14,15,104]. However, the effect of development on the gut microbiome can be exerted even independently of other factors, such as diet or environmental conditions, as shown in the southern catfish *Silurus meridionalis* [105]. Conversely, the general structure of the intestinal microbiota may remain stable during development as it has been observed in several fish species [90]. In farmed gilthead sea bream, changes were observed as the fish grew from an average body weight of 10 g to 200 g, and finally 350 g within a year [35]. Body weight was also a differentiating factor for the microbiota of wild freshwater *Gymnocypris chilianensis* [106], where heavier individuals were linked to richer and more diverse communities. This increase in diversity is generally observed during development [11], with opposing trends recorded in certain fish, such as salmon [104,107]. In our case, such effect was only observed in the wild population from Messolonghi, with community diversity remaining stable in all other populations.

Tissue localization exerted the weakest effect among the tested parameters. Differentially structured microbiomes between the upper and lower regions of the digestive tract were observed in only two sample groups: 150 g fish from Tholi (wild), and 300 g fish from Astakos (aquaculture). Generally, microbiota composition varies in different regions of the intestinal tract, affected by parameters such as anatomy, secretions, pH, redox potential, dissolved particles, as well as passage rate and residence time of digested food [15,48]. In *S. aurata*, the pH gradually increases from 3.5 to 6.1 in the stomach to 6.8–8.2 in the midgut and hindgut. Moreover, stomach evacuation is fast, with 30–40% of the digesta reaching the midgut within two hours after feeding [108]. Differences in local conditions, and the functional role of individual bacterial genera may have contributed to the observed variation between the two regions of the digestive tract in the two samples groups. For example, the predominance of *Staphylococcus* in the upper digestive tract of fish from Astakos may be linked to its capacity to develop in low pH [109], whereas the dominance of *Photobacterium* in the lower part of the digestive tract of fish from Tholi may reflect its role in the degradation of chitinous compounds [11,110,111]. Conversely, the observed similarity in bacterial communities between the two regions of the digestive tract in most experimental groups may be due to the inclusion of the transient microbiota in the analysis, which likely contributed to the homogenization of microbial profiles between the upper and lower sections.

### 4.4. Taxonomic Composition at the Phylum Level

At the phylum level, our experimental groups displayed a similar pattern with most of the available works describing the intestinal microbiota of gilthead sea bream, which were characterized by the prevalence of Pseudomonadota, followed by Bacillota, Actinomycetota, and Bacteroidota [20,21,22,23]. However, in our case, Bacteroidota were identified with slightly lower relative abundance than Deinococcota (3.8% against 5.3%). Conversely, several studies have reported dominance of Bacillota instead of Pseudomonadota [22,25,26]. Despite this observed variation in microbiota composition, the first two phyla dominated the intestinal bacterial community in both wild and aquaculture groups, following trends observed in several other fish species [8,11,14,17,48,80]. Additionally, a higher Pseudomonadota to Bacillota ratio was observed in wild populations compared to fish of aquaculture origin. The balance between these phyla has been associated with different trophic levels or dietary regimes. In this context, herbivores or fish fed with plant-based diets generally contain more Bacillota, while carnivorous species and fish reared on animal-based diets are predominated by Pseudomonadota [11,17,112,113]. However, the decrease in Pseudomonadota in our farmed samples was not accompanied by an increase in the relative abundance of Bacillota, but instead by a rise in other phyla, such as Actinomycetota and Deinococcota. The most prevalent representative genus of Actinomycetota was *Micrococcus*¸which may play fundamentally different roles in fish physiology. A *Micrococcus* strain exhibited beneficial probiotic effects in Nile tilapia [114], while others acted as opportunistic pathogens in several fish species, including Nile tilapia [115,116]. Dominance of *Micrococcus* was previously observed in the resident microbiota of juvenile *S. aurata* after an 86 h fasting period [76]. Deinococcota were represented by the genus *Allomeiothermus*, which emerged from the reclassification of the genus *Meiothermus* and currently comprises a single species, *Allomeiothermus silvanus* [117]. Several occurrences of *Meiothermus* have been recorded in fish microbiota studies [118,119,120]. The genus was identified in Atlantic salmon reared with commercial diets, and three experimental diets with high plant-protein content (80%) during a diet replacement experiment [121]. Therefore, its increased presence in aquaculture fish could be associated with the use of commercial feed or the presence of ingredients of plant origin in it. These factors, along with the different environmental variables and management procedures that were discussed previously, could also explain the observed decrease in Pseudomonadota.

### 4.5. Community Composition and Interactions at the Genus Level

Genera such as *Acinetobacter*, *Aeromonas*, *Bacillus*, *Enterobacter*, *Geobacillus*, *Micrococcus*, *Pantoea*, *Pseudomonas*, *Psychrobacter*, and *Staphylococcus* were common to all populations and body growth stages, forming a probable core microbiome. Most of these genera are typical residents in the intestine of *S*. *aurata* [20,23,25,32,35,122,123,124] and are often implicated in nutrient metabolism, modulation of immune responses, biofilm formation, and pathogenicity [11,14,16,17,125]. More specifically, *Acinetobacter*, *Bacillus*, *Pseudomonas*, and *Staphylococcus* were identified as core members in a recent meta-analysis in gilthead sea bream [122] along with other genera, such as *Clostridium*, *Corynebacterium*, *Enterococcus Paracoccus*, *Sphingomonas*, *Streptococcus* and *Vibrio*. These genera were also identified in our NGS or culture dependent analysis but not as members of the core microbiota, either due to low occurrence or abundance. *Geobacillus* is the only core member not frequently detected in gilthead sea bream and may be considered a member of the transient gut microbiota acquired through feeding, although it formed positive interactions with several genera, including *Vibrio*, in wild populations. It has been previously identified at low relative abundance in gilthead sea bream reared on insect-based diets [25]. However, the genus is known to be prevalent in the intestinal microbial communities associated with other fish species [126,127,128].

Differentially abundant core genera and unique microbial signatures differentiated the experimental groups. In wild fish, the strong presence of *Psychrobacter*, *Aeromonas*, *Acinetobacter*, *Photobacterium*, and *Pseudomonas* may reflect influence of diet and environmental conditions. These bacteria may aid with the digestion of nutrients by producing amylases, proteases, lipases, or, in the case of *Photobacterium*, chitinases [11,17,129]. These genera also host species and strains that are implicated in serious disease in *S. aurata*, such as *Aeromonas salmonicida* [130], *Photobacterium damselae* [131,132], and *Pseudomonas anguilliseptica* [133,134], and representatives that may be linked to opportunistic disease due to dysbiosis [14,135]. The generally less diverse communities of aquaculture fish showed a stronger presence of *Vibrio*, *Allomeiothermus*, *Micrococcus*, *Geobacillus*, and *Halalkalibacter*.

*Psychrobacter*, for instance, has been associated with the surrounding water but also with multiple fish tissues, such as the skin, gills, gut, and kidney of healthy fish [14,136,137,138]. It has been identified in the intestinal tract of carnivore and zooplanktivore fish [11], and it may have a dual role in physiology, displaying either probiotic action [139,140,141], or cause opportunistic infections [14,142,143]. Interestingly, *Psychrobacter*-1573 interacted positively with *Enterobacter* and *Staphylococcus* OTUs and was identified as a core member in both growth stages of wild fish. These results strongly suggest that this taxon, identified as *Psychrobacter psychrophilus*, might be a putative member of the resident intestinal microbiota of the studied wild populations. On the contrary, the other three *Psychrobacter* OTUs formed only negative interactions with the remaining community of wild gilthead sea bream. However, *Psychrobacter*-4 was a member of the core microbiota of both wild and aquaculture samples, and in aquaculture fish it formed positive interactions with two more taxa. Therefore, there are indications that *Psychrobacter*-4 (highest similarity with *P. pulmonis* and *P*. *faecalis*) is also a resident member, at least in aquaculture fish. This notion was further supported by the culturable diversity, as 22 highly similar strains to *Psychrobacter pulmonis* were isolated from both groups. The last two *Psychrobacter* OTUs seem to be transient bacteria acquired through feed or the environment, since they interacted negatively with the remaining members of the community.

Several *Aeromonas* taxa seem to be putative members of the resident microbiota of the studied *S. aurata* populations, involved in mutualistic relationships with the host. Three of them, otu1527, otu1773, and otu2003, were involved in a series of positive interactions with the rest of the bacterial community in both wild and aquaculture populations. They constituted members of the core microbiota in Tholi and 300 g wild fish, and although they were not identified as core microbiota in aquaculture populations, they were present with high relative abundance in Vonitsa. In this context, several *Aeromonas* species displayed beneficial effects to their hosts, by conferring resistance against pathogenic *Vibrio* or *Aeromonas* strains, exhibiting proteolytic and cellulolytic activities, and promoting the development of the digestive tract of *Artemia franciscana* nauplii [144,145,146,147]. On the contrary, otu11 was identified as a core member in all populations, but without forming any positive interactions with the community, except for *Aeromonas*-2003 in wild samples. Especially in aquaculture samples, otu11 formed only negative (28 in total) interactions with the rest of the community. The size of the NGS amplicon (~465 bp) did not permit precise species identification for otu11. However, the sequence exhibited the same percentage identity with several species, including *A*. *salmonicida*, *A. enterica*, *A. encheleia*, and *A*. *piscicola*. When combined with results from the culture-dependent approach, in which three isolates were identified as *A*. *salmonicida*, it seems plausible that this taxon corresponds to this species, which is a well-known fish pathogen [148]. Another interesting aspect of the *Aeromonas* taxa was the presence of a subnetwork of positive intragenus interactions. In this context, otu2003 (highest similarity with *A*. *popoffii*), developed positive associations with all other *Aeromonas* taxa, except for otu11 in aquaculture samples. These observations may suggest a high degree of synergy between *Aeromonas* species within the community, which could favor co-infections and potentially assist pathogenic strains in establishing viable populations within a host organism. In our case, it seems that an *Aeromonas* species (otu2003) that is a putative member of the resident microbiota could assist a potentially pathogenic species (otu11) associate with the host, at least in wild samples. The occurrence of multiple *Aeromonas* species has been previously reported in farmed *S. aurata* individuals, with strains isolated from the skin, gut tissue, and gills [149]. Moreover, co-infections of *Aeromonas* species were previously identified in outbreaks in European seabass (*Dicentrarchus labrax*) populations [39]. However, in that case, an antagonistic relationship was observed between *A. veronii* bv. *sobria* and *A*. *rivipollensis* during co-infection.

Despite their presence in the community of wild samples, especially as core bacteria in the population from Tholi, *Photobacterium* taxa did not develop any positive associations with the other bacterial genera. However, *Photobacterium*-47 co-associated with five different taxa of *Aeromonas*, *Vibrio* and *Rothia* in aquaculture samples. This OTU showed high similarity with *Photobacterium toruni*, a species that was previously identified as the causative agent of disease in farmed redbanded sea bream (*Pagrus auriga*) in Spain [150]. This may further suggest that other genera harboring opportunistic pathogens, such as *Aeromonas* and *Vibrio*, could facilitate the recruitment of additional pathogenic strains into the intestinal community. Conversely, *Photobacterium* taxa are often associated with the intestinal tract of carnivorous fish, where they assist with the digestion of chitin [11,110,111]. Therefore, similar symbiotic benefits could also be provided by *Photobacterium*-47, which could be disrupted in the event of dysbiosis, and eventually lead to disease.

The communities of both experimental groups harbored several core *Pseudomonas* and *Acinetobacter* taxa. In wild samples, based on the number of positive interactions with other genera, only *Acinetobacter*-19 (*A*. *guillouiae*) could be considered an autochthonous member of the community, since it was associated with *Paenibacillus*, *Vibrio*, *Alkalihalophilus*, and *Methylibium* bacteria. In a previous work by Huang et al. [151], *Acinetobacter* was among the dominant genera in the gut microbiota of 20 marine fish species, and specifically *A*. *guillouiae* was associated with digestion. The species was found to be positively correlated with protein, amino acid, and ketone metabolism, and negatively with photosynthesis-related pathways [151]. In this context, an *Acinetobacter pittii* strain has been discovered in bighead catfish (*Clarias macrocephalus*) which showed a probiotic effect by conferring resistance against pathogens [152]. *Acinetobacter guillouiae* was not identified with our culture-based approach. However, four *Acinetobacter* strains, classified as *A*. *lwoffii* and *A*. *johnsonii*, were isolated from wild samples. Most of the information about the role of these two species is linked to disease in fish [153,154], or spoilage of seafood during cold storage [155]. In this regard, *Acinetobacter johnsonii* was also identified with the NGS approach (otu178) and was characterized by only negative interactions with other bacteria in both experimental groups. However, a symbiotic role cannot be entirely excluded, at least for this species as it has been identified as a discriminant indicator of fish feeding habits, particularly for piscivores [151]. In aquaculture samples, only *Acinetobacter* otu37 and otu68 formed positive interactions (four in total) and could be considered putative members of the resident intestinal microbiota. On the contrary, five *Pseudomonas* taxa were main components of the two subnetworks of positive interactions that were observed in aquaculture samples, suggesting the important role of these taxa for binding the bacterial community. Probiotic roles of *Pseudomonas* strains have been previously recorded in multiple fish species, mainly associated with regulation of immune responses and resistance against pathogens [156]. Despite the wide range of positive interactions in aquaculture samples, *Pseudomonas* strains were isolated exclusively from wild samples, where they co-associated with only one *Aeromonas* taxon, suggesting a putative functional role also in the physiology of wild samples.

*Vibrio* species were a defining characteristic of aquaculture samples, especially in the population from Vonitsa, which was located within a landlocked gulf. Similarly, the wild population from the shallow, landlocked coastal lagoon of Messolonghi also exhibited a high abundance of *Vibrio* species. *Vibrio*-87, classified as *V*. *natriegens*—an ultrafast-growing bacterium [157]—was also identified as a core group in both populations. These findings suggest that environmental conditions are the primary factors influencing the prevalence and abundance of *Vibrio* species in local *S*. *aurata* populations, regardless of management practices. In this context, Wan et al. [158] observed higher *Vibrio* abundance and diversity in estuarine environments compared to coastal waters. In general, *Vibrio* variance and abundance is strongly driven by temperature and salinity [159]. Furthermore, in a recent study, the occurrence of *V*. *parahaemolyticus* and *V*. *vulnificus* in water, oyster, and sediment samples displayed seasonality and was affected by the aforementioned environmental parameters, and chlorophyll *a* concentration [160]. The variation in these environmental parameters between open sea and landlocked populations in our study may explain the observed differences in *Vibrio* species abundance between the two types of ecosystems. Furthermore, different *Vibrio* taxa were participating in positive interactions with other bacteria of the community in wild and aquaculture groups. In wild samples, *Vibrio*-21 (*V*. *gallaecicus*) co-associated with *Acinetobacter*, *Alkalihalophilus*, *Geobacillus*, *Halalkalibacter*, and *Paenibacillus* taxa. Conversely, in aquaculture samples, *Vibrio*-28 (*V*. *rumoiensis*) and *Vibrio*-118 (*V*. *kanaloae*) were central components of the bacterial community, collectively involved in 23 positive interactions. *Vibrio gallaecicus* and *V*. *kanaloae* are phylogenetically related and classified in the *V*. *splendidus* clade [161]. They have been isolated from sea water, and from healthy or diseased marine animals, such as fish, shrimp, and bivalves [161,162,163]. The metabolic potential of *Vibrio gallaecicus* strains was also highlighted by their high efficiency in degrading methylphosphonate (MPn) [164]. Similarly, *V. rumoiensis* is a psychrophilic bacterium with strong antioxidant potential linked to its high catalase activity [165]. The exact role of these species in the fish intestinal microbiota is not yet fully understood, but their metabolic capacity suggests that they may perform functional roles within the intestinal microbial community. As with many bacteria inhabiting the digestive tract of fish, *Vibrio* species may exert symbiotic, probiotic, or pathogenic effects on the host [11,37,159]. Common bacterial pathogens of gilthead sea bream, such as *V. anguillarum*, and *V*. *harveyii*, were also detected within our culturable diversity, but found exclusively in wild samples.

*Paenibacillus* and *Staphylococcus* comprised highly abundant and prevalent genera across the studied populations. *Staphylococcus* was also a member of the core microbiota, while *Paenibacillus* was a core member only in the population from Tholi. Their ubiquity across the samples, and strong co-occurrence with other bacterial genera suggest a key role in maintaining community stability. These positive interactions were observed in both experimental groups and involved genera such as *Aeromonas*, *Bacillus*, *Enterobacter*, *Enterococcus*, *Psychrobacter*, and *Vibrio*, among others. Two *Paenibacillus* taxa, otu36 (*P*. *mobilis*) and otu5, were mostly involved in these interactions. Notable probiotic effects have been previously reported for *Paenibacillus* strains in various fish species. These effects have been linked to enhanced nutrition by promoting growth and intestinal health, protection against pathogens by stimulating immune responses against *Aeromonas hydrophila* and *Vibrio parahaemolyticus*, and increased antioxidant activity [166,167,168,169,170]. Therefore, the presence of the two *Paenibacillus* taxa in both groups may suggest a beneficial symbiotic relationship with the host. Additionally, the lower abundance observed in aquaculture individuals compared to wild samples may contribute to decreased resistance to pathogens in farmed fish. Notably, the genus was highly abundant in 150 g fish from the landlocked sites of Messolonghi and Vonitsa, suggesting a potential influence of environmental conditions and the body weight of fish on the presence of *Paenibacillus*. In the case of *Staphylococcus*, two taxa were involved in positive interactions: otu907 (*S. xylosus*) in wild fish and otu646 (*S*. *equorum*) in aquaculture samples. *Staphylococcus xylosus* is generally considered a commensal nonpathogenic bacterium associated with the skin and mucous membranes of animals [171]. However, it has been linked with opportunistic infections. Specifically, in fish, it has been reported as a causative agent of exopthalmia and increased mortality in rainbow trout (*Oncorhynchus mykiss*) [172]. *Staphylococcus xylosus* and *S*. *equorum* have been identified in various fermented products, including meat, milk, and cheese, and are known to exhibit antioxidant properties as well as proteolytic and lipolytic activities [171,173,174,175]. Therefore, it is highly likely that these species exert beneficial symbiotic effects within the digestive tract of fish.

### 4.6. Implications for Aquaculture Fish Health and Management

The reduction in the intestinal bacterial diversity observed in aquaculture populations may have serious implications for host health. A diverse microbiota is generally crucial for effective growth, development of the immune system, and resistance against pathogens [14,77]. The observed changes in composition, particularly the reduction in putative beneficial taxa such as *Paenibacillus* and the increase in opportunistic pathogens, such as *Vibrio* and *Micrococcus*, may also contribute to adverse effects in host health. However, the precise function of each bacterial genus within the studied communities remains unclear, as multiple genera with potentially beneficial or pathogenic roles were identified. In this context, taxa belonging to *Vibrio*, *Photobacterium*, and *Aeromonas* were key components of the microbiota, co-occurring with several other members, even though these genera are known to include pathogens for gilthead sea bream [176]. These findings highlight the need for microbiome-based interventions in aquaculture, as the intensive use of antibiotics or disinfectants may inadvertently impact beneficial bacteria that support the microbiota of farmed fish due to shared genomic traits with the targeted pathogens. Tailored probiotic, prebiotic, or synbiotic formulations could help enrich beneficial microbial taxa, while the use of diverse diets, and reduced antibiotic use, may help preserve or restore gut microbiota diversity [10,14,15,77,168].

Several practical interventions emerge from our findings: (1) Probiotic supplementation with *Paenibacillus* species, which were reduced in farmed fish but associated with pathogen resistance in wild populations; (2) Evaluation of isolated *Psychrobacter pulmonis* and *Bacillus subtilis* strains for antagonistic activity against *Vibrio* pathogens; (3) Environmental management strategies including optimized stocking density, incorporation of natural diet components, and water quality monitoring to maintain beneficial microbial diversity. The 220 bacterial strains isolated in this study represent a valuable resource for systematic screening of probiotic candidates with potential applications in *S. aurata* aquaculture. Intestinal microbiota are integral to fish health and understanding the major drivers of microbial composition helps in designing better feeds, improving aquaculture sustainability and reducing disease outbreaks.

### 4.7. Limitations and Future Directions

High-throughput sequencing and culture-based studies have advanced our understanding of fish microbial communities. However, the complex methodologies they rely on may be prone to several limitations and biases. In this study, sampling took place at the end of autumn and thus the observed diversity reflects the status of the bacterial communities at this time. Since the intestinal microbiota of fish exhibits seasonal variation [15,177], it is highly likely that the structure and composition of the communities change throughout the year or across years, influenced by changes in environmental conditions or available diet. In this context, wild and aquaculture samples were collected approximately one month apart. Therefore, although both sampling events were conducted within the same autumn period, the possibility that some of the observed differences between the two groups were influenced by seasonal variation cannot be entirely excluded. The same applies for interannual fluctuations of the gut microbiota, as the results represent the microbiota of samples captured in a specific year (2017). Moreover, a short period (~1–2 h) intervened between the collection and subsequent processing of the samples in the laboratory that may have influenced the downstream analysis of bacterial profiles. Εach gut sample that was used for DNA extraction comprised both the mucosa-associated and the digesta-associated microbiota. Analyzing these two components separately would allow for a more precise characterization of the resident and transient gut microbiota of the studied populations. The observed culturable diversity may have been affected by the number of samples, as tissues from three individuals were used for the culture-dependent analysis. Additionally, the culture-based approach did not provide quantitative information regarding the total bacterial load of the studied tissues, as its primary focus was the identification of candidate strains for probiotic evaluation. Bias can also be introduced by the selected PCR protocols, the sensitivity and specificity of the primers that are used, the presence of multiple 16S rRNA copies, the sequencing technology that is employed, as well as the data analysis pipelines and the reference databases that are used for taxonomic classification [81,178]. While 16S rRNA sequencing provides taxonomic insights, it lacks functional resolution. Although several bioinformatic tools can be used to predict functional properties based on taxonomic composition, their accuracy depends on the limited availability of bacterial genomic data from fish hosts in public databases [14]. Other more direct approaches such as metagenomic, metatranscriptomic or metabolomic studies would be valuable to link taxonomic profiles with gene content, enzymatic activity, and metabolite production. Experimental work could also evaluate the efficacy of isolated strains as probiotics or dietary supplements on growth, immunity, and disease resistance in farmed *S. aurata*.

## 5. Conclusions

This study provides a comprehensive comparative analysis of the intestinal microbiota in wild and aquaculture populations of gilthead sea bream (*Sparus aurata*) from western Greece, integrating high-throughput 16S rRNA gene sequencing with culture-based approaches. Our findings demonstrate that the gut microbial community structure is shaped by multiple interacting factors, with rearing conditions and environmental origin exerting the strongest influence, followed by the growth stage, while tissue localization within the gastrointestinal tract had a comparatively minor effect.

Wild populations consistently harbored more diverse and evenly distributed bacterial communities compared to aquaculture fish, a pattern that was particularly evident in the culture-dependent analysis where 28 genera were isolated from wild samples versus only five from farmed fish. This reduction in microbial diversity under intensive farming conditions may have significant implications for host health, immune function, and resilience to environmental stressors and disease.

Despite the observed differences between experimental groups, a conserved core microbiome comprising *Psychrobacter*, *Staphylococcus*, *Geobacillus*, *Aeromonas*, *Enterobacter*, *Pantoea*, *Bacillus*, and *Acinetobacter* was identified across all populations and growth stages, suggesting fundamental host-microbe associations that persist regardless of environmental conditions. Notably, network analysis revealed that certain bacterial taxa, particularly *Aeromonas* species, form complex co-occurrence relationships that could potentially facilitate establishment of pathogenic strains—a finding with direct implications for disease management strategies in intensive aquaculture systems.

The differential abundance of specific bacterial taxa between wild and aquaculture populations provides valuable insights into aquaculture management. The reduction in potentially beneficial genera such as *Paenibacillus* and the increased prevalence of opportunistic pathogens including *Vibrio* and *Micrococcus* in farmed fish highlight the need for microbiome-targeted interventions. The bacterial strains isolated in this study represent candidates for future evaluation as probiotics or dietary supplements to restore microbial diversity and enhance disease resistance in farmed *S. aurata*.

These findings support the integration of microbiome management strategies into sustainable aquaculture practices. Future research should employ genomic, metagenomic, metatranscriptomic, and metabolomic approaches to elucidate the functional roles of identified taxa, while experimental studies are needed to validate the probiotic potential of isolated strains for improving growth performance, immune responses, and disease resistance in commercially farmed gilthead sea bream.

## Data Availability

Raw sequencing data have been deposited in the NCBI Sequence Read Archive (SRA) under BioProject accession number PRJNA1393174. The 16S rRNA gene sequences of cultured isolates have been deposited in GenBank under accession numbers PX756504-PX756578. Processed OTU tables, sample metadata, and analysis scripts are available upon reasonable request to the corresponding author.

## Supplementary Materials

The following supporting information can be downloaded at: https://www.mdpi.com/article/10.3390/microorganisms14030708/s1, Supplementary_material_Final.docx. Supplementary Figure S1: Geographic distribution of wild and aquaculture *Sparus* *aurata* sampling sites across western Greece. Aquaculture samples are shown on the map as red dots and wild samples as yellow dots. Sites include Vonitsa (1), Astakos (2), Tholi (3), and Messolonghi (4). Map created with ArcGIS Online (https://www.arcgis.com). Supplementary Figure S2: Dissected *Sparus aurata* gut showing two defined sections used for microbial analysis: the upper part of the digestive tract (UpD) [esophagus (ES), stomach (ST), and pyloric caeca (PC)] and the lower part of the digestive tract (LowD) [midgut (MG), and hindgut (HG)]. Each region was sampled separately to evaluate spatial variation in gut microbiota composition. Supplementary Figure S3: Pairwise comparison of PERMANOVA *p*-values between sample groups after merging. Supplementary Figure S4. Alpha diversity of bacterial communities associated with the gastrointestinal tract of wild and farmed fish. Different letters denote statistically significant differences between groups. Supplementary Figure S5. Relative abundance of bacterial phyla identified in the gut samples of *S. aurata*. Statistical significance of observed differences in relative abundance was calculated using pairwise Wilcoxon rank-sum tests. Stars denote different *p*-value ranges. ***: *p* ≤ 0.001, **: 0.001 < *p* ≤ 0.01, *: 0.01 < *p* ≤ 0.05. Supplementary Figure S6. Differentially abundant genera in the studied samples. To reduce complexity, the eight most abundant genera, involving 165 pairwise comparisons, are shown in the bar plots. Statistical significance of observed differences in relative abundance was calculated using pairwise Wilcoxon rank-sum tests. Stars denote different *p*-value ranges. ***: *p* ≤ 0.001, **: 0.001 < *p* ≤ 0.01, *: 0.01 < *p* ≤ 0.05. Supplementary Figure S7. Differences in abundance of bacterial genera based on the origin, populations and growth phase of collected samples. Statistical significance (S) was calculated using pairwise Wilcoxon rank-sum tests. Stars denote different *p*-value ranges. ***: *p* ≤ 0.001, **: 0.001 < *p* ≤ 0.01, *: 0.01 < *p* ≤ 0.05. AqC: Aquaculture; MES: Messolonghi; THL: Tholi; VNT: Vonitsa; AST: Astakos. Supplementary Figure S8. Significant interactions between bacterial OTUs. Nodes (circles) represent bacterial OTUs and edges (lines) interactions. Red edges correspond to mutual exclusions and green edges copresence. (A) The complete network of interactions within the microbiota of wild samples. (B) Interactions formed by *Paenibacillus*-5, the prevalent bacterium in wild samples. (C) The complete network of interactions between community members in aquaculture samples. (D) The interactions of the two most abundant bacteria in aquaculture samples, *Psychrobacter*-4 and *Vibrio*-3. Supplementary Figure S9. The relative abundance of bacterial strains isolated from the gut tissue of *S. aurata* samples. UpD: Upper digestive tract; LowD: Lower digestive tract. Supplementary Table S1. Samples used in the next generation sequencing and culture dependent analysis. The digestive tract of individual fish was separated into two parts: the upper digestive (UpD) and the lower digestive tract (LowD). Supplementary Table S2. The detailed list of 117 OTUs that were identified with more than 0.1% relative abundance across samples in the amplicon sequencing analysis, including their taxonomy and relative abundance (RA%) in the dataset. The twenty OTUs with the highest relative abundances are highlighted in grey. The standard error (SE) is also included in the last column. Supplementary Table S3. The presence of bacterial phyla and classes in the studied gut samples. RA%: Percentage relative abundance; SE: Standard error. Supplementary Table S4. PERMANOVA *p*-values for different sample groups. Upper and lower digestive tissues exhibiting *p*-values > 0.05 were merged as they contained highly similar communities. Merged groups are highlighted in orange. Supplementary Table S5. Alpha diversity of bacterial communities associated with the gastrointestinal tract of wild and farmed fish. Supplementary Table S6. Core and unique bacteria in gut samples of *S. aurata* based on the collection area. MES: Messolonghi; THL; Tholi; VNT: Vonitsa; AST: Astakos. Supplementary Table S7. Core and unique bacteria in the gastrointestinal tract of *S. aurata* according to the body weight. Wild_150: 150-g wild fish; Wild_300: 300-g wild fish; AqC_150: 150-g aquaculture fish; AqC_300: 300-g aquaculture fish. Supplementary Table S8. The number and the relative abundance of isolated bacteria from wild and aquaculture samples based on their taxonomic assignment at the genus level.

## References

[1] FAO (2023). The State of Mediterranean and Black Sea Fisheries 2023.

[2] Hellenic Aquaculture Producers Organisation (HAPO) (2025). Aquaculture Annual Report 2025.

[3] FAO (2024). The State of World Fisheries and Aquaculture 2024. Blue Transformation in Action; The State of World Fisheries and Aquaculture (SOFIA).

[4] Gephart J.A., Golden C.D., Asche F., Belton B., Brugere C., Froehlich H.E., Fry J.P., Halpern B.S., Hicks C.C., Jones R.C. (2020). Scenarios for Global Aquaculture and Its Role in Human Nutrition. Rev. Fish. Sci. Aquac..

[5] Pradeepkiran J.A. (2019). Aquaculture Role in Global Food Security with Nutritional Value: A Review. Trans. Anim. Sci..

[6] Boyd C.E., McNevin A.A., Davis R.P. (2022). The Contribution of Fisheries and Aquaculture to the Global Protein Supply. Food Secur..

[7] Gupta S., Vera-Ponce de León A., Kodama M., Hoetzinger M., Clausen C.G., Pless L., Verissimo A.R.A., Stengel B., Calabuig V., Kvingedal R. (2024). The Need for High-Resolution Gut Microbiome Characterization to Design Efficient Strategies for Sustainable Aquaculture Production. Commun. Biol..

[8] Kanika N.H., Liaqat N., Chen H., Ke J., Lu G., Wang J., Wang C. (2025). Fish Gut Microbiome and Its Application in Aquaculture and Biological Conservation. Front. Microbiol..

[9] Lorgen-Ritchie M., Uren Webster T., McMurtrie J., Bass D., Tyler C.R., Rowley A., Martin S.A.M. (2023). Microbiomes in the Context of Developing Sustainable Intensified Aquaculture. Front. Microbiol..

[10] Perry W.B., Lindsay E., Payne C.J., Brodie C., Kazlauskaite R. (2020). The Role of the Gut Microbiome in Sustainable Teleost Aquaculture. Proc. R. Soc. B Biol. Sci..

[11] Egerton S., Culloty S., Whooley J., Stanton C., Ross R.P. (2018). The Gut Microbiota of Marine Fish. Front. Microbiol..

[12] Merrifield D.L., Rodiles A. (2015). The Fish Microbiome and Its Interactions with Mucosal Tissues. Mucosal Health in Aquaculture.

[13] Chen C.-Z., Li P., Liu L., Li Z.-H. (2022). Exploring the Interactions between the Gut Microbiome and the Shifting Surrounding Aquatic Environment in Fisheries and Aquaculture: A Review. Environ. Res..

[14] Legrand T.P.R.A., Wynne J.W., Weyrich L.S., Oxley A.P.A. (2020). A Microbial Sea of Possibilities: Current Knowledge and Prospects for an Improved Understanding of the Fish Microbiome. Rev. Aquac..

[15] Ringø E., Zhou Z., Vecino J.L.G., Wadsworth S., Romero J., Krogdahl Å., Olsen R.E., Dimitroglou A., Foey A., Davies S. (2016). Effect of Dietary Components on the Gut Microbiota of Aquatic Animals. A Never-Ending Story?. Aquac. Nutr..

[16] Luan Y., Li M., Zhou W., Yao Y., Yang Y., Zhang Z., Ringø E., Erik Olsen R., Liu Clarke J., Xie S. (2023). The Fish Microbiota: Research Progress and Potential Applications. Engineering.

[17] Ou W., Yu G., Zhang Y., Mai K. (2021). Recent Progress in the Understanding of the Gut Microbiota of Marine Fishes. Mar. Life Sci. Technol..

[18] Tolas I., Zhou Z., Zhang Z., Teame T., Olsen R.E., Ringø E., Rønnestad I. (2025). A Fishy Gut Feeling—Current Knowledge on Gut Microbiota in Teleosts. Front. Mar. Sci..

[19] Kuz’mina V.V. (2008). Classical and Modern Concepts in Fish Digestion. Feeding and Digestive Functions in Fishes.

[20] Domingo-Bretón R., Cools S., Moroni F., Belenguer A., Calduch-Giner J.A., Croes E., Holhorea P.G., Naya-Català F., Boon H., Pérez-Sánchez J. (2025). Intestinal Microbiota Shifts by Dietary Intervention during Extreme Heat Summer Episodes in Farmed Gilthead Sea Bream (*Sparus aurata*). Aquac. Rep..

[21] Moroni F., Naya-Català F., Piazzon M.C., Rimoldi S., Calduch-Giner J., Giardini A., Martínez I., Brambilla F., Pérez-Sánchez J., Terova G. (2021). The Effects of Nisin-Producing *Lactococcus lactis* Strain Used as Probiotic on Gilthead Sea Bream (*Sparus aurata*) Growth, Gut Microbiota, and Transcriptional Response. Front. Mar. Sci..

[22] Nikouli E., Meziti A., Antonopoulou E., Mente E., Kormas K.A. (2018). Gut Bacterial Communities in Geographically Distant Populations of Farmed Sea Bream (*Sparus aurata*) and Sea Bass (*Dicentrarchus labrax*). Microorganisms.

[23] Ruiz A., Alós J., Gisbert E., Furones D., Viver T. (2024). Long-Term Adaptation to Dietary Shifts of Gut Microbiota in Gilthead Seabream (*Sparus aurata*). Front. Mar. Sci..

[24] Mhalhel K., Levanti M., Abbate F., Laurà R., Guerrera M.C., Aragona M., Porcino C., Briglia M., Germanà A., Montalbano G. (2023). Review on Gilthead Seabream (*Sparus aurata*) Aquaculture: Life Cycle, Growth, Aquaculture Practices and Challenges. J. Mar. Sci. Eng..

[25] Rimoldi S., Di Rosa A.R., Oteri M., Chiofalo B., Hasan I., Saroglia M., Terova G. (2024). The Impact of Diets Containing *Hermetia*
*Illucens* Meal on the Growth, Intestinal Health, and Microbiota of Gilthead Seabream (*Sparus aurata*). Fish. Physiol. Biochem..

[26] Estruch G., Collado M.C., Peñaranda D.S., Vidal A.T., Cerdá M.J., Martínez G.P., Martinez-Llorens S. (2015). Impact of Fishmeal Replacement in Diets for Gilthead Sea Bream (*Sparus aurata*) on the Gastrointestinal Microbiota Determined by Pyrosequencing the 16S rRNA Gene. PLoS ONE.

[27] Naya-Català F., Wiggers G.A., Piazzon M.C., López-Martínez M.I., Estensoro I., Calduch-Giner J.A., Martínez-Cuesta M.C., Requena T., Sitjà-Bobadilla A., Miguel M. (2021). Modulation of Gilthead Sea Bream Gut Microbiota by a Bioactive Egg White Hydrolysate: Interactions Between Bacteria and Host Lipid Metabolism. Front. Mar. Sci..

[28] García-Márquez J., Domínguez-Maqueda M., Torres M., Cerezo I.M., Ramos E., Alarcón F.J., Mancera J.M., Martos-Sitcha J.A., Moriñigo M.Á., Balebona M.C. (2023). Potential Effects of Microalgae-Supplemented Diets on the Growth, Blood Parameters, and the Activity of the Intestinal Microbiota in *Sparus aurata* and *Mugil cephalus*. Fishes.

[29] García-Márquez J., Rico R.M., Acién F.G., Mancera J.M., Figueroa F.L., Vizcaíno A.J., Alarcón F.J., Moriñigo M.Á., Abdala-Díaz R.T. (2023). Dietary Effects of a Short-Term Administration of Microalgae Blend on Growth Performance, Tissue Fatty Acids, and Predominant Intestinal Microbiota in *Sparus aurata*. Microorganisms.

[30] Katsoulis-Dimitriou S., Nikouli E., Gkalogianni E.Z., Karapanagiotidis I.T., Kormas K.A. (2024). The effect of dietary fish oil replacement by microalgae on the gilthead sea bream midgut bacterial microbiota. Peer Community J..

[31] Fontinha F., Magalhães R., Moutinho S., Santos R., Campos P., Serra C.R., Aires T., Oliva-Teles A., Peres H. (2021). Effect of Dietary Poultry Meal and Oil on Growth, Digestive Capacity, and Gut Microbiota of Gilthead Seabream (*Sparus aurata*) Juveniles. Aquaculture.

[32] Panteli N., Mastoraki M., Lazarina M., Chatzifotis S., Mente E., Kormas K.A., Antonopoulou E. (2021). Configuration of Gut Microbiota Structure and Potential Functionality in Two Teleosts under the Influence of Dietary Insect Meals. Microorganisms.

[33] Randazzo B., Zarantoniello M., Cardinaletti G., Cerri R., Giorgini E., Belloni A., Contò M., Tibaldi E., Olivotto I. (2021). *Hermetia Illucens* and Poultry By-Product Meals as Alternatives to Plant Protein Sources in Gilthead Seabream (*Sparus aurata*) Diet: A Multidisciplinary Study on Fish Gut Status. Animals.

[34] Tampou A., Kousoulaki K., Vasilaki A., Vlahos N., Nikouli E., Panteli N., Feidantsis K., Kormas K., Andreopoulou S., Karapanagiotidis I.T. (2025). Growth Performance of Gilthead Sea Bream (*Sparus aurata*) Fed Low Fish Meal Diets with an Innovative Mixture of Low Trophic Ingredients. Aquac. Nutr..

[35] Naya-Català F., Piazzon M.C., Torrecillas S., Toxqui-Rodríguez S., Calduch-Giner J.À., Fontanillas R., Sitjà-Bobadilla A., Montero D., Pérez-Sánchez J. (2022). Genetics and Nutrition Drive the Gut Microbiota Succession and Host-Transcriptome Interactions through the Gilthead Sea Bream (*Sparus aurata*) Production Cycle. Biology.

[36] Rahayu S., Amoah K., Huang Y., Cai J., Wang B., Shija V.M., Jin X., Anokyewaa M.A., Jiang M. (2024). Probiotics Application in Aquaculture: Its Potential Effects, Current Status in China and Future Prospects. Front. Mar. Sci..

[37] Sanches-Fernandes G.M.M., Sá-Correia I., Costa R. (2022). Vibriosis Outbreaks in Aquaculture: Addressing Environmental and Public Health Concerns and Preventive Therapies Using Gilthead Seabream Farming as a Model System. Front. Microbiol..

[38] Ina-Salwany M.Y., Al-saari N., Mohamad A., Mursidi F.-A., Mohd-Aris A., Amal M.N.A., Kasai H., Mino S., Sawabe T., Zamri-Saad M. (2019). Vibriosis in Fish: A Review on Disease Development and Prevention. J. Aquat. Anim. Health.

[39] Cantillo Villa Y., Triga A., Katharios P. (2023). Polyinfection in Fish Aeromoniasis: A Study of Co-Isolated *Aeromonas* Species in *Aeromonas veronii* Outbreaks. Pathogens.

[40] Triga A., Smyrli M., Katharios P. (2023). Pathogenic and Opportunistic *Vibrio* Spp. Associated with Vibriosis Incidences in the Greek Aquaculture: The Role of *Vibrio*
*Harveyi* as the Principal Cause of Vibriosis. Microorganisms.

[41] Avella M.A., Gioacchini G., Decamp O., Makridis P., Bracciatelli C., Carnevali O. (2010). Application of Multi-Species of *Bacillus* in Sea Bream Larviculture. Aquaculture.

[42] Carnevali O., Maradonna F., Gioacchini G. (2017). Integrated Control of Fish Metabolism, Wellbeing and Reproduction: The Role of Probiotic. Aquaculture.

[43] Chen Z., Ceballos-Francisco D., Guardiola F.A., Esteban M.Á. (2020). Dietary Administration of the Probiotic *Shewanella putrefaciens* to Experimentally Wounded Gilthead Seabream (*Sparus aurata* L.) Facilitates the Skin Wound Healing. Sci. Rep..

[44] Guzmán-Villanueva L.T., Tovar-Ramírez D., Gisbert E., Cordero H., Guardiola F.A., Cuesta A., Meseguer J., Ascencio-Valle F., Esteban M.A. (2014). Dietary Administration of β-1,3/1,6-Glucan and Probiotic Strain *Shewanella putrefaciens*, Single or Combined, on Gilthead Seabream Growth, Immune Responses and Gene Expression. Fish Shellfish. Immunol..

[45] Suzer C., Çoban D., Kamaci H.O., Saka Ş., Firat K., Otgucuoğlu Ö., Küçüksari H. (2008). *Lactobacillus* Spp. Bacteria as Probiotics in Gilthead Sea Bream (*Sparus aurata*, L.) Larvae: Effects on Growth Performance and Digestive Enzyme Activities. Aquaculture.

[46] Yıldırım Ş., Suzer C., Fırat K., Saka Ş., Hekimoğlu M., Çoban D., Korkut A.Y., Köse İ., Antepli O., Gökvardar A. (2024). Impact of Probiotic *Bacillus* Sp. Dietary Supplementation on Pancreatic and Intestinal Activities in Seabream *Sparus aurata*. Lett. Appl. Microbiol..

[47] Ghanbari M., Kneifel W., Domig K.J. (2015). A New View of the Fish Gut Microbiome: Advances from next-Generation Sequencing. Aquaculture.

[48] Wang A.R., Ran C., Ringø E., Zhou Z.G. (2018). Progress in Fish Gastrointestinal Microbiota Research. Rev. Aquac..

[49] Georgiou N., Fakiris E., Koutsikopoulos C., Papatheodorou G., Christodoulou D., Dimas X., Geraga M., Kapellonis Z.G., Vaziourakis K.-M., Noti A. (2021). Spatio-Seasonal Hypoxia/Anoxia Dynamics and Sill Circulation Patterns Linked to Natural Ventilation Drivers, in a Mediterranean Landlocked Embayment: Amvrakikos Gulf, Greece. Geosciences.

[50] Ramfos A., Alysandratou A., Katsani O., Faulwetter S., Nikolakopoulos K., Avramidis P. (2024). Ecological and Environmental Characteristics of a Seagrass-Dominated Hypersaline Coastal Mediterranean Lagoon: A Multidisciplinary Approach. Aquat. Sci..

[51] Klindworth A., Pruesse E., Schweer T., Peplies J., Quast C., Horn M., Glöckner F.O. (2013). Evaluation of General 16S Ribosomal RNA Gene PCR Primers for Classical and Next-Generation Sequencing-Based Diversity Studies. Nucleic Acids Res..

[52] Sambrook J., Maniatis T., Fritsch E.F. (1989). Molecular Cloning: A Laboratory Manual.

[53] Edgar R.C. (2010). Search and Clustering Orders of Magnitude Faster than BLAST. Bioinformatics.

[54] Edgar R.C. (2013). UPARSE: Highly Accurate OTU Sequences from Microbial Amplicon Reads. Nat. Methods.

[55] Edgar R.C. (2018). UNCROSS2: Identification of Cross-Talk in 16S rRNA OTU Tables. bioRxiv.

[56] Bolyen E., Rideout J.R., Dillon M.R., Bokulich N.A., Abnet C.C., Al-Ghalith G.A., Alexander H., Alm E.J., Arumugam M., Asnicar F. (2019). Reproducible, Interactive, Scalable and Extensible Microbiome Data Science Using QIIME 2. Nat. Biotechnol..

[57] Camacho C., Coulouris G., Avagyan V., Ma N., Papadopoulos J., Bealer K., Madden T.L. (2009). BLAST+: Architecture and Applications. BMC Bioinform..

[58] Quast C., Pruesse E., Yilmaz P., Gerken J., Schweer T., Yarza P., Peplies J., Glöckner F.O. (2013). The SILVA Ribosomal RNA Gene Database Project: Improved Data Processing and Web-Based Tools. Nucleic Acids Res..

[59] Good I. (1953). The Population Frequencies of Species and the Estimation of Population Parameters. Biometrika.

[60] Oksanen J., Blanchet F.G., Friendly M., Kindt R., Legendre P., McGlinn D., Wagner H. (2017). Vegan: Community Ecology Package.

[61] Liu C., Cui Y., Li X., Yao M. (2021). Microeco: An R Package for Data Mining in Microbial Community Ecology. FEMS Microbiol. Ecol..

[62] Bel Mokhtar N., Asimakis E., Galiatsatos I., Maurady A., Stathopoulou P., Tsiamis G. (2024). Development of MetaXplore: An Interactive Tool for Targeted Metagenomic Analysis. Curr. Issues Mol. Biol..

[63] Faust K., Raes J. (2016). CoNet App: Inference of Biological Association Networks Using Cytoscape. F1000Res.

[64] Shannon P., Markiel A., Ozier O., Baliga N.S., Wang J.T., Ramage D., Amin N., Schwikowski B., Ideker T. (2003). Cytoscape: A Software Environment for Integrated Models of Biomolecular Interaction Networks. Genome Res..

[65] Bastian M., Heymann S., Jacomy M. (2009). Gephi: An Open Source Software for Exploring and Manipulating Networks. Proc. Int. AAAI Conf. Web Soc. Media.

[66] Jacomy M., Venturini T., Heymann S., Bastian M. (2014). ForceAtlas2, a Continuous Graph Layout Algorithm for Handy Network Visualization Designed for the Gephi Software. PLoS ONE.

[67] Woodman M.E. (2008). Direct PCR of Intact Bacteria (Colony PCR). Curr. Protoc. Microbiol..

[68] Lane D.J., Stackebrandt E., Goodfellow M. (1991). 16S/23S rRNA Sequencing. Nucleic Acids Techniques in Bacterial Systematics.

[69] Kim J., Hong J., Lim J.-A., Heu S., Roh E. (2018). Improved Multiplex PCR Primers for Rapid Identification of Coagulase-Negative Staphylococci. Arch. Microbiol..

[70] Weisburg W.G., Barns S.M., Pelletier D.A., Lane D.J. (1991). 16S Ribosomal DNA Amplification for Phylogenetic Study. J. Bacteriol..

[71] Kearse M., Moir R., Wilson A., Stones-Havas S., Cheung M., Sturrock S., Buxton S., Cooper A., Markowitz S., Duran C. (2012). Geneious Basic: An Integrated and Extendable Desktop Software Platform for the Organization and Analysis of Sequence Data. Bioinformatics.

[72] Altschul S.F., Gish W., Miller W., Myers E.W., Lipman D.J. (1990). Basic Local Alignment Search Tool. J. Mol. Biol..

[73] Edgar R.C. (2004). MUSCLE: Multiple Sequence Alignment with High Accuracy and High Throughput. Nucleic Acids Res..

[74] Letunic I., Bork P. (2021). Interactive Tree Of Life (iTOL) v5: An Online Tool for Phylogenetic Tree Display and Annotation. Nucleic Acids Res..

[75] Kormas K.A., Meziti A., Mente E., Frentzos A. (2014). Dietary Differences Are Reflected on the Gut Prokaryotic Community Structure of Wild and Commercially Reared Sea Bream (*Sparus aurata*). MicrobiologyOpen.

[76] Viver T., Ruiz A., Bertomeu E., Martorell-Barceló M., Urdiain M., Grau A., Signaroli M., Barcelo-Serra M., Aspillaga E., Pons A. (2023). Food Determines Ephemerous and Non-Stable Gut Microbiome Communities in Juvenile Wild and Farmed Mediterranean Fish. Sci. Total Environ..

[77] Infante-Villamil S., Huerlimann R., Jerry D.R. (2021). Microbiome Diversity and Dysbiosis in Aquaculture. Rev. Aquac..

[78] Nayak S.K. (2010). Role of Gastrointestinal Microbiota in Fish. Aquac. Res..

[79] See M.S., Ching X.L., Khoo S.C., Abidin S.Z., Sonne C., Ma N.L. (2025). Aquatic Microbiomes under Stress: The Role of Gut Microbiota in Detoxification and Adaptation to Environmental Exposures. J. Hazard. Mater. Adv..

[80] Singh B.K., Thakur K., Kumari H., Mahajan D., Sharma D., Sharma A.K., Kumar S., Singh B., Pankaj P.P., Kumar R. (2025). A Review on Comparative Analysis of Marine and Freshwater Fish Gut Microbiomes: Insights into Environmental Impact on Gut Microbiota. FEMS Microbiol. Ecol..

[81] Tarnecki A.M., Burgos F.A., Ray C.L., Arias C.R. (2017). Fish Intestinal Microbiome: Diversity and Symbiosis Unravelled by Metagenomics. J. Appl. Microbiol..

[82] Piazzon M.C., Calduch-Giner J.A., Fouz B., Estensoro I., Simó-Mirabet P., Puyalto M., Karalazos V., Palenzuela O., Sitjà-Bobadilla A., Pérez-Sánchez J. (2017). Under Control: How a Dietary Additive Can Restore the Gut Microbiome and Proteomic Profile, and Improve Disease Resilience in a Marine Teleostean Fish Fed Vegetable Diets. Microbiome.

[83] Solé-Jiménez P., Naya-Català F., Piazzon M.C., Estensoro I., Calduch-Giner J.À., Sitjà-Bobadilla A., Van Mullem D., Pérez-Sánchez J. (2021). Reshaping of Gut Microbiota in Gilthead Sea Bream Fed Microbial and Processed Animal Proteins as the Main Dietary Protein Source. Front. Mar. Sci..

[84] Rimoldi S., Gliozheni E., Ascione C., Gini E., Terova G. (2018). Effect of a Specific Composition of Short- and Medium-Chain Fatty Acid 1-Monoglycerides on Growth Performances and Gut Microbiota of Gilthead Sea Bream (*Sparus aurata*). PeerJ.

[85] Silva F.C.d.P., Nicoli J.R., Zambonino-Infante J.L., Kaushik S., Gatesoupe F.-J. (2011). Influence of the Diet on the Microbial Diversity of Faecal and Gastrointestinal Contents in Gilthead Sea Bream (*Sparus aurata*) and Intestinal Contents in Goldfish (*Carassius auratus*). FEMS Microbiol. Ecol..

[86] Nikouli E., Meziti A., Antonopoulou E., Mente E., Kormas K.A. (2019). Host-Associated Bacterial Succession during the Early Embryonic Stages and First Feeding in Farmed Gilthead Sea Bream (*Sparus aurata*). Genes.

[87] Kuz’mina V.V., Skvortsova E.G., Shalygin M.V., Kovalenko K.E. (2015). Role of Peptidases of the Intestinal Microflora and Prey in Temperature Adaptations of the Digestive System in Planktivorous and Benthivorous Fish. Fish Physiol. Biochem..

[88] Lilli G., Sirot C., Campbell H., Hermand F., Brophy D., Flot J.-F., Graham C.T., George I.F. (2024). Do Fish Gut Microbiotas Vary across Spatial Scales? A Case Study of *Diplodus vulgaris* in the Mediterranean Sea. Anim. Microbiome.

[89] Najafpour B., Pinto P.I.S., Sanz E.C., Martinez-Blanch J.F., Canario A.V.M., Moutou K.A., Power D.M. (2023). Core Microbiome Profiles and Their Modification by Environmental, Biological, and Rearing Factors in Aquaculture Hatcheries. Mar. Pollut. Bull..

[90] Pan B., Han X., Yu K., Sun H., Mu R., Lian C.-A. (2023). Geographical Distance, Host Evolutionary History and Diet Drive Gut Microbiome Diversity of Fish across the Yellow River. Mol. Ecol..

[91] Kountoura K., Zacharias I. (2013). Trophic State and Oceanographic Conditions of Amvrakikos Gulf: Evaluation and Monitoring. Desalination Water Treat..

[92] Kountoura K., Zacharias I. (2011). Temporal and Spatial Distribution of Hypoxic/Seasonal Anoxic Zone in Amvrakikos Gulf, Western Greece. Estuar. Coast. Shelf Sci..

[93] Kountoura K., Zacharias I. (2014). Annual Hypoxia Dynamics in a Semi-Enclosed Mediterranean Gulf. J. Mar. Syst..

[94] Mavropoulou A.-M., Vervatis V., Sofianos S. (2020). Dissolved Oxygen Variability in the Mediterranean Sea. J. Mar. Syst..

[95] Ramfos A., Isari S., Somarakis S., Georgopoulos D., Koutsikopoulos C., Fragopoulu N. (2006). Mesozooplankton Community Structure in Offshore and Coastal Waters of the Ionian Sea (Eastern Mediterranean) during Mixed and Stratified Conditions. Mar. Biol..

[96] Souvermezoglou E., Krasakopoulou E. (2021). Temporal Evolution of Oxygen and Nutrients in the NE Ionian Sea from 1987 to 2008 under the Influence of the Cretan and Adriatic Seas. Prog. Oceanogr..

[97] Toxqui-Rodríguez S., Holhorea P.G., Naya-Català F., Calduch-Giner J.À., Sitjà-Bobadilla A., Piazzon C., Pérez-Sánchez J. (2024). Differential Reshaping of Skin and Intestinal Microbiota by Stocking Density and Oxygen Availability in Farmed Gilthead Sea Bream (*Sparus aurata*): A Behavioral and Network-Based Integrative Approach. Microorganisms.

[98] Basak C., Chakraborty R. (2024). Effect of Hypoxia on the Gut Microflora of a Facultative Air-Breathing Loach *Lepidocephalichthys guntea*. Curr. Microbiol..

[99] Sun S., Yang M., Fu H., Ge X., Zou J. (2020). Altered Intestinal Microbiota Induced by Chronic Hypoxia Drives the Effects on Lipid Metabolism and the Immune Response of Oriental River Prawn *Macrobrachium nipponense*. Aquaculture.

[100] Wang W., Huang J., Zhang J., Wang Z., Li H., Amenyogbe E., Chen G. (2021). Effects of Hypoxia Stress on the Intestinal Microflora of Juvenile of Cobia (*Rachycentron canadum*). Aquaculture.

[101] Avramidis P., Nikolaou K., Poulos K., Bekiari V., Vantarakis A. (2017). Environmental Characterization of a Mediterranean Protected Shallow Brackish Coastal Aquatic System, Klisova Lagoon, Western Greece: A Case Study. J. Coast. Conserv..

[102] Zenetos A., Christianidis S., Pancucci M., Simboura N., Tziavos C., Pancucci M., Simboura N., Tziavos C. (1997). Oceanologic Study of an Open Coastal Area in the Ionian Sea with Emphasis on Its Benthic Fauna and Some Zoogeographical Remarks. Ocean. Acta.

[103] Faulwetter S., Simboura N., Katsiaras N., Chatzigeorgiou G., Arvanitidis C. (2017). Polychaetes of Greece: An Updated and Annotated Checklist. Biodivers. Data J..

[104] Tawfik M.M., Lorgen-Ritchie M., Król E., McMillan S., Norambuena F., Bolnick D.I., Douglas A., Tocher D.R., Betancor M.B., Martin S.A.M. (2024). Modulation of Gut Microbiota Composition and Predicted Metabolic Capacity after Nutritional Programming with a Plant-Rich Diet in Atlantic Salmon (*Salmo salar*): Insights across Developmental Stages. Anim. Microbiome.

[105] Zhang Z., Li D., Refaey M.M., Xu W., Tang R., Li L. (2018). Host Age Affects the Development of Southern Catfish Gut Bacterial Community Divergent From That in the Food and Rearing Water. Front. Microbiol..

[106] Zhao Z., Zhao H., Zhang L., Huang Z., Ke H., Liu Y., Duan Y., Li H., Wang X., Li Q. (2023). Integrated Analysis of How Gender and Body Weight Affect the Intestinal Microbial Diversity of Gymnocypris Chilianensis. Sci. Rep..

[107] Lokesh J., Kiron V., Sipkema D., Fernandes J.M.O., Moum T. (2019). Succession of Embryonic and the Intestinal Bacterial Communities of Atlantic Salmon (*Salmo salar*) Reveals Stage-Specific Microbial Signatures. MicrobiologyOpen.

[108] Nikolopoulou D., Moutou K.A., Fountoulaki E., Venou B., Adamidou S., Alexis M.N. (2011). Patterns of Gastric Evacuation, Digesta Characteristics and pH Changes along the Gastrointestinal Tract of Gilthead Sea Bream (*Sparus aurata* L.) and European Sea Bass (*Dicentrarchus labrax* L.). Comp. Biochem. Physiol. Part A Mol. Integr. Physiol..

[109] Zhou C., Fey P.D. (2020). The Acid Response Network of *Staphylococcus aureus*. Curr. Opin. Microbiol..

[110] Itoi S., Okamura T., Koyama Y., Sugita H. (2006). Chitinolytic Bacteria in the Intestinal Tract of Japanese Coastal Fishes. Can. J. Microbiol..

[111] Le Doujet T., De Santi C., Klemetsen T., Hjerde E., Willassen N.-P., Haugen P. (2019). Closely-Related *Photobacterium* Strains Comprise the Majority of Bacteria in the Gut of Migrating Atlantic Cod (*Gadus morhua*). Microbiome.

[112] Desai A.R., Links M.G., Collins S.A., Mansfield G.S., Drew M.D., Van Kessel A.G., Hill J.E. (2012). Effects of Plant-Based Diets on the Distal Gut Microbiome of Rainbow Trout (*Oncorhynchus mykiss*). Aquaculture.

[113] Rimoldi S., Terova G., Ascione C., Giannico R., Brambilla F. (2018). Next Generation Sequencing for Gut Microbiome Characterization in Rainbow Trout (*Oncorhynchus mykiss*) Fed Animal by-Product Meals as an Alternative to Fishmeal Protein Sources. PLoS ONE.

[114] Abd El-Rhman A.M., Khattab Y.A.E., Shalaby A.M.E. (2009). *Micrococcus luteus* and *Pseudomonas* Species as Probiotics for Promoting the Growth Performance and Health of Nile Tilapia, *Oreochromis niloticus*. Fish Shellfish. Immunol..

[115] Pękala A., Paździor E., Antychowicz J., Bernad A., Głowacka H., Więcek B., Niemczuk W. (2018). *Kocuria rhizophila* and *Micrococcus luteus* as Emerging Opportunist Pathogens in Brown Trout (*Salmo trutta* Linnaeus, 1758) and Rainbow Trout (*Oncorhynchus mykiss* Walbaum, 1792). Aquaculture.

[116] Suresh K., Pillai D., Soni M., Rathlavath S., Narshivudu D. (2024). *Micrococcus luteus*, an Emerging Opportunistic Pathogen in Farmed Nile Tilapia, *Oreochromis niloticus* in Andhra Pradesh, India. Aquacult Int..

[117] Jiao J.-Y., Lian Z.-H., Liu Z.-T., Liu L., Li M.-M., Lv A.-P., Xian W.-D., Zhou T., Lyu Z., Salam N. (2022). Genome-Based Reclassification of the Genus *Meiothermus* along with the Proposal of a New Genus *Allomeiothermus* Gen. Nov.. Antonie Leeuwenhoek.

[118] Anka I.Z., Uren Webster T.M., Berbel-Filho W.M., Hitchings M., Overland B., Weller S., Garcia de Leaniz C., Consuegra S. (2024). Microbiome and Epigenetic Variation in Wild Fish with Low Genetic Diversity. Nat. Commun..

[119] Le M.H., Wang D. (2020). Structure and Membership of Gut Microbial Communities in Multiple Fish Cryptic Species under Potential Migratory Effects. Sci. Rep..

[120] Sumithra T.G., Sharma K.S.R., Gangadharan S., Suresh G., Prasad V., Amala P.V., Sayooj P., Gop A.P., Anil M.K., Patil P.K. (2022). Dysbiosis and Restoration Dynamics of the Gut Microbiome Following Therapeutic Exposure to Florfenicol in Snubnose Pompano (*Trachinotus blochii*) to Aid in Sustainable Aquaculture Production Strategies. Front. Microbiol..

[121] Egerton S., Wan A., Murphy K., Collins F., Ahern G., Sugrue I., Busca K., Egan F., Muller N., Whooley J. (2020). Replacing Fishmeal with Plant Protein in Atlantic Salmon (*Salmo salar*) Diets by Supplementation with Fish Protein Hydrolysate. Sci. Rep..

[122] Moroni F., Naya-Català F., Hafez A.I., Domingo-Bretón R., Soriano B., Llorens C., Pérez-Sánchez J. (2025). Beyond Microbial Variability: Disclosing the Functional Redundancy of the Core Gut Microbiota of Farmed Gilthead Sea Bream from a Bayesian Network Perspective. Microorganisms.

[123] Nikouli E., Meziti A., Smeti E., Antonopoulou E., Mente E., Kormas K.A. (2021). Gut Microbiota of Five Sympatrically Farmed Marine Fish Species in the Aegean Sea. Microb. Ecol..

[124] Salgueiro V., Manageiro V., Bandarra N.M., Reis L., Ferreira E., Caniça M. (2020). Bacterial Diversity and Antibiotic Susceptibility of *Sparus aurata* from Aquaculture. Microorganisms.

[125] Zhang B., Yang H., Cai G., Nie Q., Sun Y. (2024). The Interactions between the Host Immunity and Intestinal Microorganisms in Fish. Appl. Microbiol. Biotechnol..

[126] Liu Q., Lai Z., Gao Y., Wang C., Zeng Y., Liu E., Mai Y., Yang W., Li H. (2021). Connection between the Gut Microbiota of Largemouth Bass (*Micropterus salmoides*) and Microbiota of the Pond Culture Environment. Microorganisms.

[127] Sun H., Chen F., Hao H., Wang K.-J. (2022). Multi-Dimensional Investigation and Distribution Characteristics Analysis of Gut Microbiota of Different Marine Fish in Fujian Province of China. Front. Microbiol..

[128] Zhao R., Symonds J.E., Walker S.P., Steiner K., Carter C.G., Bowman J.P., Nowak B.F. (2023). Relationship between Gut Microbiota and Chinook Salmon (*Oncorhynchus tshawytscha*) Health and Growth Performance in Freshwater Recirculating Aquaculture Systems. Front. Microbiol..

[129] Ray A.K., Ghosh K., Ringø E. (2012). Enzyme-Producing Bacteria Isolated from Fish Gut: A Review. Aquac. Nutr..

[130] Real Valcárcel F. (2021). Aeromonas Salmonicida Infection in *Sparus aurata* in the Canaries. Bull.—Eur. Assoc. Fish Pathol..

[131] Freitas I.L., Teixeira A., Loureiro I., Lisboa J., Saraiva A., dos Santos N.M.S., do Vale A. (2022). Susceptibility of Sea Bream (*Sparus aurata*) to AIP56, an AB-Type Toxin Secreted by *Photobacterium damselae* Subsp. *Piscicida*. Toxins.

[132] Santos P., Peixoto D., Ferreira I., Passos R., Pires P., Simões M., Pousão-Ferreira P., Baptista T., Costas B. (2022). Short-Term Immune Responses of Gilthead Seabream (*Sparus aurata*) Juveniles against *Photobacterium damselae* Subsp. *Piscicida*. Int. J. Mol. Sci..

[133] Doménech A., Fernández-Garayzábal J.F., García J.A., Cutuli M.T., Blanco M., Gibello A., Moreno M.A., Domínguez L. (1999). Association of *Pseudomonas anguilliseptica* Infection with ‘Winter Disease’ in Sea Bream, *Sparus aurata* L.. J. Fish. Dis..

[134] Fadel A., Mabrok M., Aly S. (2018). Epizootics of *Pseudomonas anguilliseptica* among Cultured Seabream (*Sparus aurata*) Populations: Control and Treatment Strategies. Microb. Pathog..

[135] Mougin J., Joyce A. (2023). Fish Disease Prevention via Microbial Dysbiosis-Associated Biomarkers in Aquaculture. Rev. Aquac..

[136] Bel Mokhtar N., Apostolopoulou G., Koumoundouros G., Tzokas K., Toskas K., Gourzioti E., Stathopoulou P., Tsiamis G. (2024). Bacterial Community Structures and Dynamics Associated with Rotated Positioning Syndrome in Gilthead Sea Bream (*Sparus aurata*) Larviculture. Front. Aquac..

[137] Quero G.M., Piredda R., Basili M., Maricchiolo G., Mirto S., Manini E., Seyfarth A.M., Candela M., Luna G.M. (2023). Host-Associated and Environmental Microbiomes in an Open-Sea Mediterranean Gilthead Sea Bream Fish Farm. Microb. Ecol..

[138] Rosati S., Maiuro L., Lombardi S.J., Iaffaldano N., Di Iorio M., Cariglia M., Lopez F., Cofelice M., Tremonte P., Sorrentino E. (2025). Integrated Biotechnological Strategies for the Sustainability and Quality of Mediterranean Sea Bass (*Dicentrarchus labrax*) and Sea Bream (*Sparus aurata*). Foods.

[139] Makled S.O., Hamdan A.M., El-Sayed A.-F.M., Hafez E.E. (2017). Evaluation of Marine Psychrophile, *Psychrobacter namhaensis* SO89, as a Probiotic in Nile Tilapia (*Oreochromis niloticus*) Diets. Fish Shellfish. Immunol..

[140] Sun Y.-Z., Yang H.-L., Ma R.-L., Zhang C.-X., Lin W.-Y. (2011). Effect of Dietary Administration of *Psychrobacter* Sp. on the Growth, Feed Utilization, Digestive Enzymes and Immune Responses of Grouper *Epinephelus coioides*. Aquac. Nutr..

[141] Wuertz S., Beça F., Kreuz E., Wanka K.M., Azeredo R., Machado M., Costas B. (2023). Two Probiotic Candidates of the Genus *Psychrobacter* Modulate the Immune Response and Disease Resistance after Experimental Infection in Turbot (*Scophthalmus maximus*, Linnaeus 1758). Fishes.

[142] El-Sayed M.R., Emam A.M., Osman A.E., Abd El-Galil M.A.E.-A.A., Sayed H.H. (2023). Detection and Description of a Novel *Psychrobacter glacincola* Infection in Some Red Sea Marine Fishes in Hurghada, Egypt. BMC Vet. Res..

[143] McCarthy U., Anderson H., Donald K., Garden A., Weir S.J. (2013). *Psychrobacter* Sp. Isolated from the Kidney of Salmonids at a Number of Aquaculture Sites in Scotland. Bull. Eur. Assoc. Fish Pathol..

[144] Chi C., Jiang B., Yu X.-B., Liu T.-Q., Xia L., Wang G.-X. (2014). Effects of Three Strains of Intestinal Autochthonous Bacteria and Their Extracellular Products on the Immune Response and Disease Resistance of Common Carp, *Cyprinus carpio*. Fish Shellfish. Immunol..

[145] Gibson L.F., Woodworth J., George A.M. (1998). Probiotic Activity of *Aeromonas media* on the Pacific Oyster, *Crassostrea gigas*, When Challenged with *Vibrio tubiashii*. Aquaculture.

[146] Gunasekara R.A.Y.S.A., Rekecki A., Baruah K., Bossier P., Van den Broeck W. (2010). Evaluation of Probiotic Effect of *Aeromonas hydrophila* on the Development of the Digestive Tract of Germ-Free *Artemia franciscana* Nauplii. J. Exp. Mar. Biol. Ecol..

[147] Rohman A.F., Atitus I.N., Heraswati D.D., Istiqomah I., Isnansetyo A. (2021). Isolation of *Aeromonas sobria* JC18 from Milkfish (*Chanos chanos*) Intestine with Proteolytic and Cellulolytic Activities for Fish Probiotic. IOP Conf. Ser. Earth Environ. Sci..

[148] Qin X., Li Z., Guo J., Bai F., Ling X. (2025). Beyond Fish Pathogens: A Comprehensive Overview of *Aeromonas salmonicida*. Microbiol. Res..

[149] Scarano C., Piras F., Virdis S., Ziino G., Nuvoloni R., Dalmasso A., De Santis E.P.L., Spanu C. (2018). Antibiotic Resistance of *Aeromonas* Ssp. Strains Isolated from *Sparus aurata* Reared in Italian Mariculture Farms. Int. J. Food Microbiol..

[150] Labella A.M., Arahal D.R., Lucena T., Manchado M., Castro D., Borrego J.J. (2017). *Photobacterium toruni* Sp. Nov., a Bacterium Isolated from Diseased Farmed Fish. Int. J. Syst. Evol. Microbiol..

[151] Huang Q., Sham R.C., Deng Y., Mao Y., Wang C., Zhang T., Leung K.M.Y. (2020). Diversity of Gut Microbiomes in Marine Fishes Is Shaped by Host-Related Factors. Mol. Ecol..

[152] Bunnoy A., Na-Nakorn U., Kayansamruaj P., Srisapoome P. (2019). Acinetobacter Strain KUO11TH, a Unique Organism Related to *Acinetobacter pittii* and Isolated from the Skin Mucus of Healthy Bighead Catfish and Its Efficacy Against Several Fish Pathogens. Microorganisms.

[153] Bi B., Yuan Y., Jia D., Jiang W., Yan H., Yuan G., Gao Y. (2023). Identification and Pathogenicity of Emerging Fish Pathogen *Acinetobacter johnsonii* from a Disease Outbreak in Rainbow Trout (*Oncorhynchus mykiss*). Aquac. Res..

[154] Cao S., Geng Y., Yu Z., Deng L., Gan W., Wang K., Ou Y., Chen D., Huang X., Zuo Z. (2018). *Acinetobacter lwoffii*, an Emerging Pathogen for Fish in *Schizothorax* Genus in China. Transbound. Emerg. Dis..

[155] Wang X.-Y., Xie J. (2021). Comparison of Physicochemical Changes and Water Migration of *Acinetobacter johnsonii*, *Shewanella putrefaciens*, and Cocultures From Spoiled Bigeye Tuna (*Thunnus obesus*) During Cold Storage. Front. Microbiol..

[156] Ringø E., Li X., van Doan H., Ghosh K. (2022). Interesting Probiotic Bacteria Other Than the More Widely Used Lactic Acid Bacteria and Bacilli in Finfish. Front. Mar. Sci..

[157] Hoff J., Daniel B., Stukenberg D., Thuronyi B.W., Waldminghaus T., Fritz G. (2020). *Vibrio natriegens*: An Ultrafast-Growing Marine Bacterium as Emerging Synthetic Biology Chassis. Environ. Microbiol..

[158] Wan S.H., Xu Y., Xu W., Leung S.K.K., Yu E.Y.N., Yung C.C.M. (2025). Environmental Heterogeneity Drives Ecological Differentiation in *Vibrio* Populations Across Subtropical Marine Habitats. Environ. Microbiol..

[159] Takemura A.F., Chien D.M., Polz M.F. (2014). Associations and Dynamics of Vibrionaceae in the Environment, from the Genus to the Population Level. Front. Microbiol..

[160] Brumfield K.D., Chen A.J., Gangwar M., Usmani M., Hasan N.A., Jutla A.S., Huq A., Colwell R.R. (2023). Environmental Factors Influencing Occurrence of *Vibrio parahaemolyticus* and *Vibrio vulnificus*. Appl. Environ. Microbiol..

[161] Beaz-Hidalgo R., Balboa S., Romalde J.L., Figueras M.J. (2010). Diversity and Pathogenecity of *Vibrio* Species in Cultured Bivalve Molluscs. Environ. Microbiol. Rep..

[162] Beaz-Hidalgo R., Doce A., Pascual J., Toranzo A.E., Romalde J.L. (2009). *Vibrio gallaecicus* Sp. Nov. Isolated from Cultured Clams in North-Western Spain. Syst. Appl. Microbiol..

[163] Thompson F.L., Thompson C.C., Li Y., Gomez-Gil B., Vandenberghe J., Hoste B., Swings J. (2003). *Vibrio kanaloae* Sp. Nov., *Vibrio pomeroyi* Sp. Nov. and *Vibrio chagasii* Sp. Nov., from Sea Water and Marine Animals. Int. J. Syst. Evol. Microbiol..

[164] Yu S.-X., Wang X., Wang Y., Wang H., Liu J., Hong W., Zhang Y., Yu M., Zhang G.-L., Thompson F. (2025). Diverse Marine *Vibrio* Species Convert Methylphosphonate to Methane. Mar. Life Sci. Technol..

[165] Yumoto I., Iwata H., Sawabe T., Ueno K., Ichise N., Matsuyama H., Okuyama H., Kawasaki K. (1999). Characterization of a Facultatively Psychrophilic Bacterium, *Vibrio rumoiensis* Sp. Nov., That Exhibits High Catalase Activity. Appl. Environ. Microbiol..

[166] Chen S.-W., Liu C.-H., Hu S.-Y. (2019). Dietary Administration of Probiotic *Paenibacillus ehimensis* NPUST1 with Bacteriocin-like Activity Improves Growth Performance and Immunity against *Aeromonas hydrophila* and *Streptococcus iniae* in Nile Tilapia (*Oreochromis niloticus*). Fish Shellfish. Immunol..

[167] Gupta A., Gupta P., Dhawan A. (2016). *Paenibacillus polymyxa* as a Water Additive Improved Immune Response of *Cyprinus carpio* and Disease Resistance against *Aeromonas hydrophila*. Aquac. Rep..

[168] Hoseinifar S.H., Faheem M., Liaqat I., Van Doan H., Ghosh K., Ringø E. (2024). Promising Probiotic Candidates for Sustainable Aquaculture: An Updated Review. Animals.

[169] Lin P.-H., Chen S.-W., Wen Z.-H., Hu S.-Y. (2022). Administration of the Potential Probiotic *Paenibacillus ehimensis* NPUST1 Enhances Expression of Indicator Genes Associated with Nutrient Metabolism, Growth and Innate Immunity against *Aeromonas hydrophila* and *Streptococcus indie* Infections in Zebrafish (*Danio rerio*). Fishes.

[170] Yang S., Jin D., Li H., Jiang L., Cui J., Huang W., Rang J., Li Y., Xia L. (2023). Screening of New *Paenibacillus polymyxa* S3 and Its Disease Resistance of Grass Carp (*Ctenopharyngodon idellus*). J. Fish Dis..

[171] Dordet-Frisoni E., Dorchies G., De Araujo C., Talon R., Leroy S. (2007). Genomic Diversity in *Staphylococcus xylosus*. Appl. Environ. Microbiol..

[172] Oh W.T., Jun J.W., Giri S.S., Yun S., Kim H.J., Kim S.G., Kim S.W., Han S.J., Kwon J., Park S.C. (2019). *Staphylococcus xylosus* Infection in Rainbow Trout (*Oncorhynchus mykiss*) As a Primary Pathogenic Cause of Eye Protrusion and Mortality. Microorganisms.

[173] Majumdar R.K., Gupta S. (2020). Isolation, Identification and Characterization of *Staphylococcus* Sp. from Indian Ethnic Fermented Fish Product. Lett. Appl. Microbiol..

[174] Rebah F.B., Frikha F., Kamoun W., Belbahri L., Gargouri Y., Miled N. (2008). Culture of *Staphylococcus xylosus* in Fish Processing By-Product-Based Media for Lipase Production. Lett. Appl. Microbiol..

[175] Stavropoulou D.A., De Maere H., Berardo A., Janssens B., Filippou P., De Vuyst L., De Smet S., Leroy F. (2018). Species Pervasiveness Within the Group of Coagulase-Negative Staphylococci Associated With Meat Fermentation Is Modulated by pH. Front. Microbiol..

[176] Balebona M.C., Zorrilla I., Moriñigo M.A., Borrego J.J. (1998). Survey of Bacterial Pathologies Affecting Farmed Gilt-Head Sea Bream (*Sparus aurata* L.) in Southwestern Spain from 1990 to 1996. Aquaculture.

[177] Liang Y., Wang Z., Gao N., Qi X., Zeng J., Cui K., Lu W., Bai S. (2024). Variations and Interseasonal Changes in the Gut Microbial Communities of Seven Wild Fish Species in a Natural Lake with Limited Water Exchange during the Closed Fishing Season. Microorganisms.

[178] Bharti R., Grimm D.G. (2021). Current Challenges and Best-Practice Protocols for Microbiome Analysis. Brief. Bioinform..

