# Supplementary material for: Comparative Analysis of the Intestinal Microbiota in Wild and Aquaculture Populations of Sparus aurata
Source: Microorganisms. 2026 Mar 21;14(3):708. doi: 10.3390/microorganisms14030708 (PMC13028688; doi:10.3390/microorganisms14030708)
Supplement: Supplementary file 1 [file microorganisms-14-00708-s001.zip › Supplementary_material_Final.pdf]

Supplementary Materials

Comparative Analysis of the intestinal microbiota in wild and aquaculture populations of *Sparus aurata*

Maria Lanara[1#], Elias Asimakis[1#], Naima Bel Mokhtar[1], Pinelopi Koutsodima[1], Costas Batargias[2], Kosmas Toskas[3], Panagiota Stathopoulou[1,4*], and George Tsiamis[1,4*]

[1]. Laboratory of Systems Microbiology and Applied Genomics, Department of Sustainable Agriculture, School of Agricultural Sciences, University of Patras, 2 George Seferi St., 30131, Agrinio, Greece
[2]. Laboratory of Applied Genetics and Fish Breeding, Department of Biology, School of Natural Sciences, University of Patras, 26504, Rio, Greece
[3]. Department of Research and Development, Avramar Aquaculture SA, 19.3 km Markopoulou-Paiania Avenue, 19002 Paiania, Greece
[4] BioDetect P.C., Stadiou St., Platani, 26504, Rio, Greece

# Both authors contributed equally to this work.

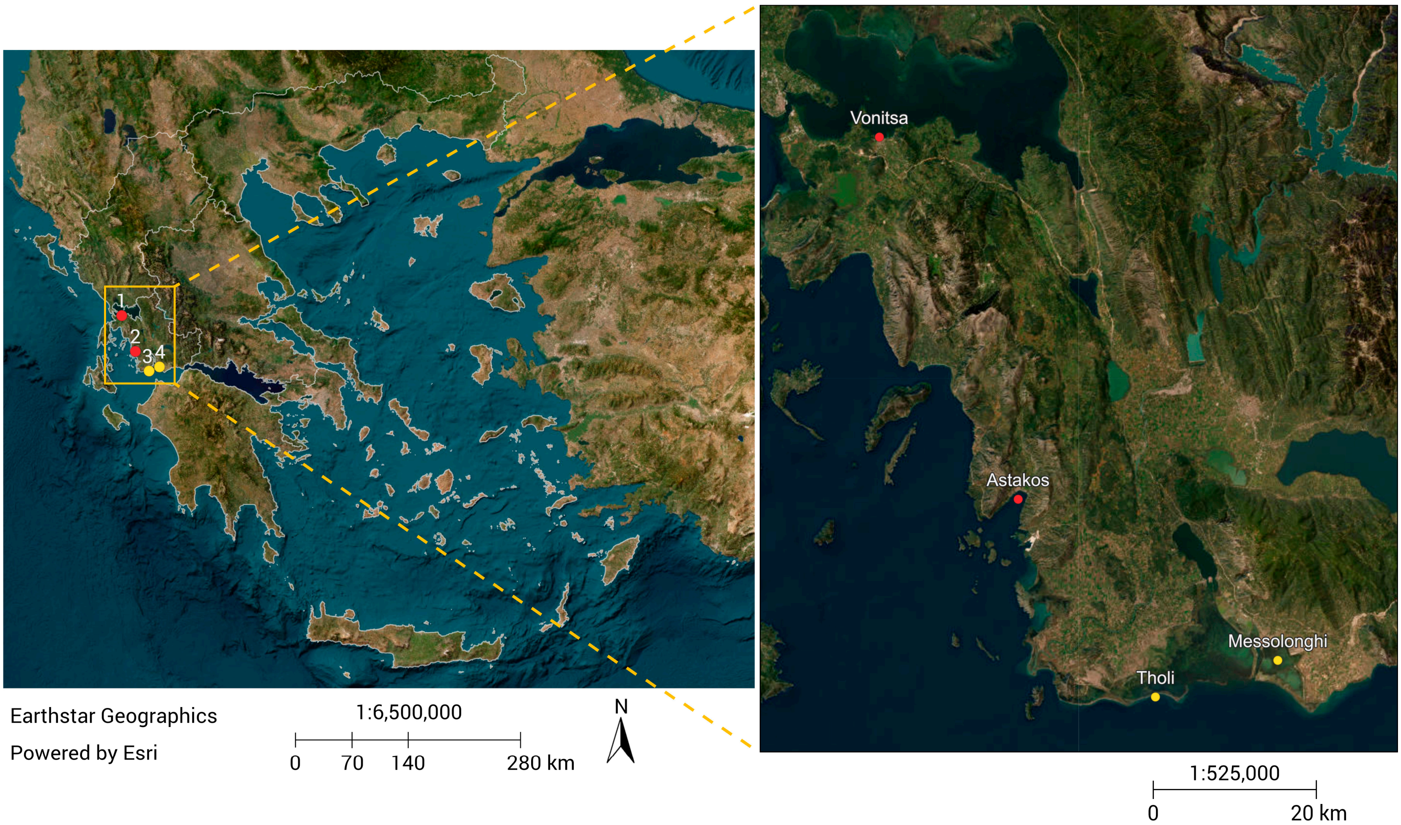

Supplementary Figure S1: Geographic distribution of wild and aquaculture *Sparus aurata* sampling sites across western Greece. Aquaculture samples are shown on the map as red dots and wild samples as yellow dots. Sites include Vonitsa (1), Astakos (2), Tholi (3), and Messolonghi (4). Map created with ArcGIS Online (https://www.arcgis.com).

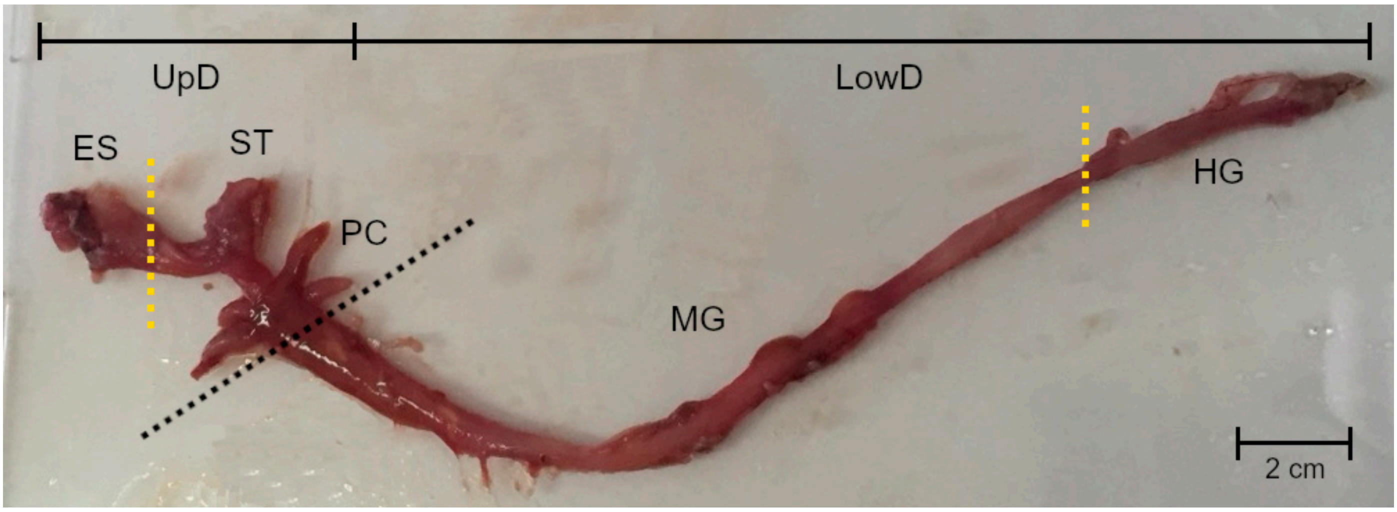

Supplementary Figure S2: Dissected *Sparus aurata* gut showing two defined sections used for microbial analysis: the upper part of the digestive tract (UpD) [esophagus (ES), stomach (ST), and pyloric caeca (PC)] and the lower part of the digestive tract (LowD) [midgut (MG), and hindgut (HG)]. Each region was sampled separately to evaluate spatial variation in gut microbiota composition.

Supplementary Table S1. Samples used in the next generation sequencing and culture dependent analysis. The digestive tract of individual fish was separated into two parts: the upper digestive (UpD) and the lower digestive tract (LowD).

		**No. of fish and tissues used for the NGS analysis**						**Fish and tissues used for cultures**		
		**150 ± 5 g**			**300 ± 5 g**			**150 ± 5 g**		
**Area**	**Type of fishery**	**Fish**	**UpD**	**LowD**	**Fish**	**UpD**	**LowD**	**Fish**	**UpD**	**LowD**
Vonitsa	Commercial (Aquaculture)	10	9	10	9	9	9	3	3	3
Astakos	Commercial (Aquaculture)	9	9	9	10	10	9	3	3	3
Messolonghi	Wild	9	9	6	9	9	9	3	3	3
Tholi	Wild	9	9	7	9	9	9	3	3	3
**Total**		**37**	**36**	**32**	**37**	**37**	**36**	**12**	**12**	**12**
**Samples used for NGS**			**68**			**73**				
**Samples used for cultures**									**24**	

Supplementary Table S2. The detailed list of 117 OTUs that were identified with more than 0.1% relative abundance across samples in the amplicon sequencing analysis, including their taxonomy and relative abundance (RA%) in the dataset. The twenty OTUs with the highest relative abundances are highlighted in grey. The standard error (SE) is also included in the last column.

OTU	Phylum	Class	Order	Family	Genus	Total RA%	SE (±)
**Otu42**	Actinomycetota	Actinobacteria	Micrococcales	Micrococcaceae	Glutamicibacter	0.64	0.12
**Otu25**	Actinomycetota	Actinobacteria	Micrococcales	Micrococcaceae	Kocuria	0.28	0.15
**Otu6**	Actinomycetota	Actinobacteria	Micrococcales	Micrococcaceae	Micrococcus	4.52	0.81
**Otu97**	Actinomycetota	Actinobacteria	Micrococcales	Micrococcaceae	Rothia	0.28	0.11
**Otu38**	Actinomycetota	Actinobacteria	Mycobacteriales	Dietziaceae	Dietzia	0.50	0.21
**Otu29**	Actinomycetota	Actinobacteria	Mycobacteriales	Nocardiaceae	Rhodococcus	0.54	0.25
**Otu55**	Actinomycetota	Actinobacteria	Propionibacteriales	Propionibacteriaceae	Cutibacterium	0.24	0.04
**Otu1120**	Bacillota	Bacilli	Bacillales	Bacillaceae	Alkalihalophilus	0.93	0.16
**Otu16**	Bacillota	Bacilli	Bacillales	Bacillaceae	Bacillus	1.67	0.31
**Otu34**	Bacillota	Bacilli	Bacillales	Bacillaceae	Bacillus	0.55	0.07
**Otu15**	Bacillota	Bacilli	Bacillales	Bacillaceae	Geobacillus	4.64	0.81
**Otu7**	Bacillota	Bacilli	Bacillales	Bacillaceae	Halalkalibacter	2.55	0.47
**Otu52**	Bacillota	Bacilli	Bacillales	Bacillaceae	Niallia	0.42	0.06
**Otu50**	Bacillota	Bacilli	Bacillales	Planococcaceae	Planococcus	0.29	0.09
**Otu60**	Bacillota	Bacilli	Brevibacillales	Brevibacillaceae	Brevibacillus	0.39	0.07
**Otu107**	Bacillota	Bacilli	Lactobacillales	Aerococcaceae	Aerococcus	0.13	0.04
**Otu89**	Bacillota	Bacilli	Lactobacillales	Enterococcaceae	Enterococcus	0.45	0.16
**Otu78**	Bacillota	Bacilli	Lactobacillales	Enterococcaceae	Enterococcus	0.18	0.11
**Otu230**	Bacillota	Bacilli	Lactobacillales	Lactobacillaceae	Ligilactobacillus	0.21	0.13
**Otu40**	Bacillota	Bacilli	Lactobacillales	Streptococcaceae	Streptococcus	0.65	0.43
**Otu212**	Bacillota	Bacilli	Lactobacillales	Streptococcaceae	Streptococcus	0.20	0.08
**Otu5**	Bacillota	Bacilli	Paenibacillales	Paenibacillaceae	Paenibacillus	5.70	0.99
**Otu20**	Bacillota	Bacilli	Paenibacillales	Paenibacillaceae	Paenibacillus	1.10	0.17
**Otu36**	Bacillota	Bacilli	Paenibacillales	Paenibacillaceae	Paenibacillus	0.71	0.14
**Otu8**	Bacillota	Bacilli	Staphylococcales	Staphylococcaceae	Staphylococcus	6.91	0.95
**Otu907**	Bacillota	Bacilli	Staphylococcales	Staphylococcaceae	Staphylococcus	1.28	0.16
**Otu646**	Bacillota	Bacilli	Staphylococcales	Staphylococcaceae	Staphylococcus	0.42	0.14
**Otu13**	Bacillota	Clostridia	Clostridiales	Clostridiaceae	Clostridium	0.79	0.40

**Otu65**	Bacteroidota	Bacteroidia	Bacteroidales	Bacteroidaceae	Bacteroides	0.14	0.09
**Otu73**	Bacteroidota	Bacteroidia	Bacteroidales	Porphyromonadaceae	Falsiporphyromonas	0.18	0.13
**Otu83**	Bacteroidota	Bacteroidia	Flavobacteriales	Flavobacteriaceae	Psychroflexus	0.19	0.15
**Otu72**	Bacteroidota	Bacteroidia	Flavobacteriales	Weeksellaceae	Chryseobacterium	0.23	0.05
**Otu43**	Bacteroidota	Bacteroidia	Flavobacteriales	Weeksellaceae	Cloacibacterium	0.42	0.14
**Otu33**	Bacteroidota	Bacteroidia	Flavobacteriales	Weeksellaceae	Wautersiella	0.89	0.55
**Otu53**	Campylobacterota	Campylobacteria	Campylobacterales	Arcobacteraceae	Arcobacter	0.24	0.19
**Otu321**	Campylobacterota	Campylobacteria	Campylobacterales	Arcobacteraceae	Arcobacter	0.17	0.16
**Otu183**	Campylobacterota	Campylobacteria	Campylobacterales	Sulfurospirillaceae	Sulfurospirillum	0.33	0.20
**Otu2**	Deinococcota	Deinococci	Thermales	Thermaceae	Allomeiothermus	5.74	0.75
**Otu69**	Fusobacteriota	Fusobacteriia	Fusobacteriales	Fusobacteriaceae	Fusobacterium	0.14	0.10
**Otu100**	Pseudomonadota	Alphaproteobacteria	Acetobacterales	Acetobacteraceae	Acetobacter	0.17	0.06
**Otu80**	Pseudomonadota	Alphaproteobacteria	Acetobacterales	Acetobacteraceae	Commensalibacter	0.25	0.12
**Otu64**	Pseudomonadota	Alphaproteobacteria	Caulobacterales	Caulobacteraceae	Brevundimonas	0.21	0.10
**Otu2823**	Pseudomonadota	Alphaproteobacteria	Caulobacterales	Caulobacteraceae	Brevundimonas	0.14	0.05
**Otu56**	Pseudomonadota	Alphaproteobacteria	Hyphomicrobiales	Beijerinckiaceae	Methylobacterium	0.20	0.03
**Otu45**	Pseudomonadota	Alphaproteobacteria	Hyphomicrobiales	Beijerinckiaceae	Methylorubrum	0.46	0.12
**Otu54**	Pseudomonadota	Alphaproteobacteria	Hyphomicrobiales	Rhizobiaceae	Brucella	0.18	0.04
**Otu88**	Pseudomonadota	Alphaproteobacteria	Hyphomicrobiales	Rhizobiaceae	Rhizobium	0.16	0.04
**Otu99**	Pseudomonadota	Alphaproteobacteria	Rhodobacterales	Paracoccaceae	Paracoccus	0.27	0.05
**Otu98**	Pseudomonadota	Alphaproteobacteria	Sphingomonadales	Sphingomonadaceae	Sphingomonas	0.19	0.03
**Otu62**	Pseudomonadota	Gammaproteobacteria	Burkholderiales	Burkholderiaceae	Ralstonia	0.18	0.05
**Otu74**	Pseudomonadota	Gammaproteobacteria	Burkholderiales	Burkholderiales_Incertae_Sedis	2013Ark19i	0.17	0.11
**Otu35**	Pseudomonadota	Gammaproteobacteria	Burkholderiales	Comamonadaceae	Acidovorax	0.37	0.16
**Otu9**	Pseudomonadota	Gammaproteobacteria	Burkholderiales	Comamonadaceae	Caldimonas	2.80	0.44
**Otu138**	Pseudomonadota	Gammaproteobacteria	Burkholderiales	Comamonadaceae	Comamonas	0.29	0.08
**Otu26**	Pseudomonadota	Gammaproteobacteria	Burkholderiales	Comamonadaceae	Delftia	0.62	0.17
**Otu49**	Pseudomonadota	Gammaproteobacteria	Burkholderiales	Comamonadaceae	Diaphorobacter	0.35	0.07
**Otu70**	Pseudomonadota	Gammaproteobacteria	Burkholderiales	Comamonadaceae	Methylibium	0.19	0.03
**Otu201**	Pseudomonadota	Gammaproteobacteria	Burkholderiales	Comamonadaceae	Simplicispira	0.18	0.09
**Otu51**	Pseudomonadota	Gammaproteobacteria	Burkholderiales	Neisseriaceae	Incertae_Sedis	0.31	0.08
**Otu123**	Pseudomonadota	Gammaproteobacteria	Burkholderiales	Oxalobacteraceae	Massilia	0.18	0.09
**Otu66**	Pseudomonadota	Gammaproteobacteria	Burkholderiales	Oxalobacteraceae	Oxalobacteraceae_bacterium	0.14	0.07

**Otu17**	Pseudomonadota	Gammaproteobacteria	Burkholderiales	Oxalobacteraceae	Undibacterium	1.09	0.43
**Otu11**	Pseudomonadota	Gammaproteobacteria	Enterobacterales	Aeromonadaceae	Aeromonas	3.59	0.56
**Otu2003**	Pseudomonadota	Gammaproteobacteria	Enterobacterales	Aeromonadaceae	Aeromonas	0.91	0.12
**Otu1773**	Pseudomonadota	Gammaproteobacteria	Enterobacterales	Aeromonadaceae	Aeromonas	0.28	0.04
**Otu1527**	Pseudomonadota	Gammaproteobacteria	Enterobacterales	Aeromonadaceae	Aeromonas	0.25	0.03
**Otu39**	Pseudomonadota	Gammaproteobacteria	Enterobacterales	Alteromonadaceae	Rheinheimera	0.36	0.32
**Otu10**	Pseudomonadota	Gammaproteobacteria	Enterobacterales	Enterobacteriaceae	Enterobacter	2.83	0.37
**Otu871**	Pseudomonadota	Gammaproteobacteria	Enterobacterales	Enterobacteriaceae	Enterobacter	1.01	0.13
**Otu44**	Pseudomonadota	Gammaproteobacteria	Enterobacterales	Enterobacteriaceae	Escherichia-Shigella	0.53	0.08
**Otu1967**	Pseudomonadota	Gammaproteobacteria	Enterobacterales	Enterobacteriaceae	Escherichia-Shigella	0.18	0.04
**Otu2784**	Pseudomonadota	Gammaproteobacteria	Enterobacterales	Enterobacteriaceae	Klebsiella	0.16	0.04
**Otu387**	Pseudomonadota	Gammaproteobacteria	Enterobacterales	Enterobacteriaceae	Pluralibacter	0.30	0.08
**Otu31**	Pseudomonadota	Gammaproteobacteria	Enterobacterales	Erwiniaceae	Pantoea	2.00	0.27
**Otu113**	Pseudomonadota	Gammaproteobacteria	Enterobacterales	Erwiniaceae	Pantoea	0.41	0.08
**Otu46**	Pseudomonadota	Gammaproteobacteria	Enterobacterales	Morganellaceae	Buchnera	0.43	0.08
**Otu195**	Pseudomonadota	Gammaproteobacteria	Enterobacterales	Morganellaceae	Candidatus_Regiella	0.15	0.05
**Otu77**	Pseudomonadota	Gammaproteobacteria	Enterobacterales	Morganellaceae	Providencia	0.49	0.14
**Otu59**	Pseudomonadota	Gammaproteobacteria	Enterobacterales	Pasteurellaceae	Haemophilus	0.38	0.28
**Otu116**	Pseudomonadota	Gammaproteobacteria	Enterobacterales	Pseudoalteromonadaceae	Pseudoalteromonas	0.17	0.11
**Otu27**	Pseudomonadota	Gammaproteobacteria	Enterobacterales	Shewanellaceae	Shewanella	0.49	0.18
**Otu47**	Pseudomonadota	Gammaproteobacteria	Enterobacterales	Vibrionaceae	Photobacterium	1.79	0.56
**Otu32**	Pseudomonadota	Gammaproteobacteria	Enterobacterales	Vibrionaceae	Photobacterium	0.68	0.16
**Otu1198**	Pseudomonadota	Gammaproteobacteria	Enterobacterales	Vibrionaceae	Photobacterium	0.16	0.10
**Otu3**	Pseudomonadota	Gammaproteobacteria	Enterobacterales	Vibrionaceae	Vibrio	6.01	1.33
**Otu21**	Pseudomonadota	Gammaproteobacteria	Enterobacterales	Vibrionaceae	Vibrio	1.18	0.28
**Otu28**	Pseudomonadota	Gammaproteobacteria	Enterobacterales	Vibrionaceae	Vibrio	0.49	0.08
**Otu87**	Pseudomonadota	Gammaproteobacteria	Enterobacterales	Vibrionaceae	Vibrio	0.39	0.06
**Otu118**	Pseudomonadota	Gammaproteobacteria	Enterobacterales	Vibrionaceae	Vibrio	0.30	0.07
**Otu79**	Pseudomonadota	Gammaproteobacteria	Enterobacterales	Yersiniaceae	Serratia	0.34	0.09
**Otu86**	Pseudomonadota	Gammaproteobacteria	Enterobacterales	Yersiniaceae	Serratia	0.16	0.04
**Otu117**	Pseudomonadota	Gammaproteobacteria	Lysobacterales	Lysobacteraceae	Stenotrophomonas	0.22	0.08
**Otu75**	Pseudomonadota	Gammaproteobacteria	Lysobacterales	Lysobacteraceae	Stenotrophomonas	0.16	0.02
**Otu19**	Pseudomonadota	Gammaproteobacteria	Pseudomonadales	Moraxellaceae	Acinetobacter	0.91	0.12

**Otu37**	Pseudomonadota	Gammaproteobacteria	Pseudomonadales	Moraxellaceae	Acinetobacter	0.91	0.15
**Otu178**	Pseudomonadota	Gammaproteobacteria	Pseudomonadales	Moraxellaceae	Acinetobacter	0.80	0.18
**Otu68**	Pseudomonadota	Gammaproteobacteria	Pseudomonadales	Moraxellaceae	Acinetobacter	0.24	0.03
**Otu84**	Pseudomonadota	Gammaproteobacteria	Pseudomonadales	Moraxellaceae	Acinetobacter	0.18	0.05
**Otu111**	Pseudomonadota	Gammaproteobacteria	Pseudomonadales	Moraxellaceae	Acinetobacter	0.13	0.06
**Otu57**	Pseudomonadota	Gammaproteobacteria	Pseudomonadales	Moraxellaceae	Cavicella	0.15	0.14
**Otu48**	Pseudomonadota	Gammaproteobacteria	Pseudomonadales	Moraxellaceae	Enhydrobacter	0.24	0.06
**Otu4**	Pseudomonadota	Gammaproteobacteria	Pseudomonadales	Moraxellaceae	Psychrobacter	8.08	1.24
**Otu1573**	Pseudomonadota	Gammaproteobacteria	Pseudomonadales	Moraxellaceae	Psychrobacter	0.70	0.09
**Otu108**	Pseudomonadota	Gammaproteobacteria	Pseudomonadales	Moraxellaceae	Psychrobacter	0.51	0.27
**Otu61**	Pseudomonadota	Gammaproteobacteria	Pseudomonadales	Moraxellaceae	Psychrobacter	0.42	0.10
**Otu225**	Pseudomonadota	Gammaproteobacteria	Pseudomonadales	Moraxellaceae	Psychrobacter	0.30	0.21
**Otu1445**	Pseudomonadota	Gammaproteobacteria	Pseudomonadales	Moraxellaceae	Psychrobacter	0.29	0.07
**Otu94**	Pseudomonadota	Gammaproteobacteria	Pseudomonadales	Moraxellaceae	Psychrobacter	0.25	0.06
**Otu24**	Pseudomonadota	Gammaproteobacteria	Pseudomonadales	Pseudomonadaceae	Pseudomonas	0.94	0.09
**Otu187**	Pseudomonadota	Gammaproteobacteria	Pseudomonadales	Pseudomonadaceae	Pseudomonas	0.71	0.18
**Otu30**	Pseudomonadota	Gammaproteobacteria	Pseudomonadales	Pseudomonadaceae	Pseudomonas	0.55	0.04
**Otu76**	Pseudomonadota	Gammaproteobacteria	Pseudomonadales	Pseudomonadaceae	Pseudomonas	0.49	0.08
**Otu1720**	Pseudomonadota	Gammaproteobacteria	Pseudomonadales	Pseudomonadaceae	Pseudomonas	0.23	0.06
**Otu2349**	Pseudomonadota	Gammaproteobacteria	Pseudomonadales	Pseudomonadaceae	Pseudomonas	0.19	0.03
**Otu1490**	Pseudomonadota	Gammaproteobacteria	Pseudomonadales	Pseudomonadaceae	Pseudomonas	0.18	0.03
**Otu18**	Spirochaetota	Brevinematia	Brevinematales	Brevinemataceae	Brevinema	0.55	0.21
**Otu41**	Verrucomicrobiota	Verrucomicrobiia	Verrucomicrobiales	Rubritaleaceae	Luteolibacter	0.34	0.10
**117**	**9**	**11**	**25**	**48**	**77**	**100**	**Total**

Supplementary Table S3. The presence of bacterial phyla and classes in the studied gut samples. RA%: Percentage relative abundance; SE: Standard error.

**Phylum**	**Class**	**RA%**	**SE (±)**
Actinomycetota	Actinobacteria	6.8	1.6
Bacillota	Bacilli	28.5	3.6
Bacillota	Clostridia	0.5	0.4
Bacteroidota	Bacteroidia	3.8	1.1
Campylobacterota	Campylobacteria	1.0	0.5
Deinococcota	Deinococci	5.3	1.7
Fusobacteriota	Fusobacteriia	0.3	0.1
Pseudomonadota	Alphaproteobacteria	2.7	0.5
Pseudomonadota	Gammaproteobacteria	50.4	3.8
Spirochaetota	Brevinematia	0.4	0.2
Verrucomicrobiota	Verrucomicrobiia	0.3	0.1

Supplementary Table S4. PERMANOVA *p*-values for different sample groups. Upper and lower digestive tissues exhibiting *p*-values > 0.05 were merged as they contained highly similar communities. Merged groups are highlighted in orange.

**Studied parameter**	**Group**	**Sample group 1**	**Sample group 2**	***p*-value**	**Status**	**New Group**
Origin	Overall	Wild	Domesticated	0.001	-	-
Population	Domesticated	AST	VNT	0.001	-	-
	Wild	MES	THL	0.001	-	-
Population and body weight	Astakos	AST_150	AST_300	0.001	-	-
	Vonitsa	VNT_150	VNT_300	0.001	-	-
	Messolonghi	MES_150	MES_300	0.001	-	-
	Tholi	THL_150	THL_300	0.001	-	-
Population, body weight, and tissue part	Astakos	AST_150_LowD	AST_150_UpD	0.441	Merged	AST_150
		AST_300_LowD	AST_300_UpD	0.03	-	-
	Vonitsa	VNT_150_LowD	VNT_150_UpD	0.339	Merged	VNT_150
		VNT_300_LowD	VNT_300_UpD	0.661	Merged	VNT_300
	Messolonghi	MES_150_LowD	MES_150_UpD	0.090	Merged	MES_150
		MES_300_LowD	MES_300_UpD	0.148	Merged	MES_300
	Tholi	THL_150_LowD	THL_150_UpD	0.014		
		THL_300_LowD	THL_300_UpD	0.222	Merged	THL_300

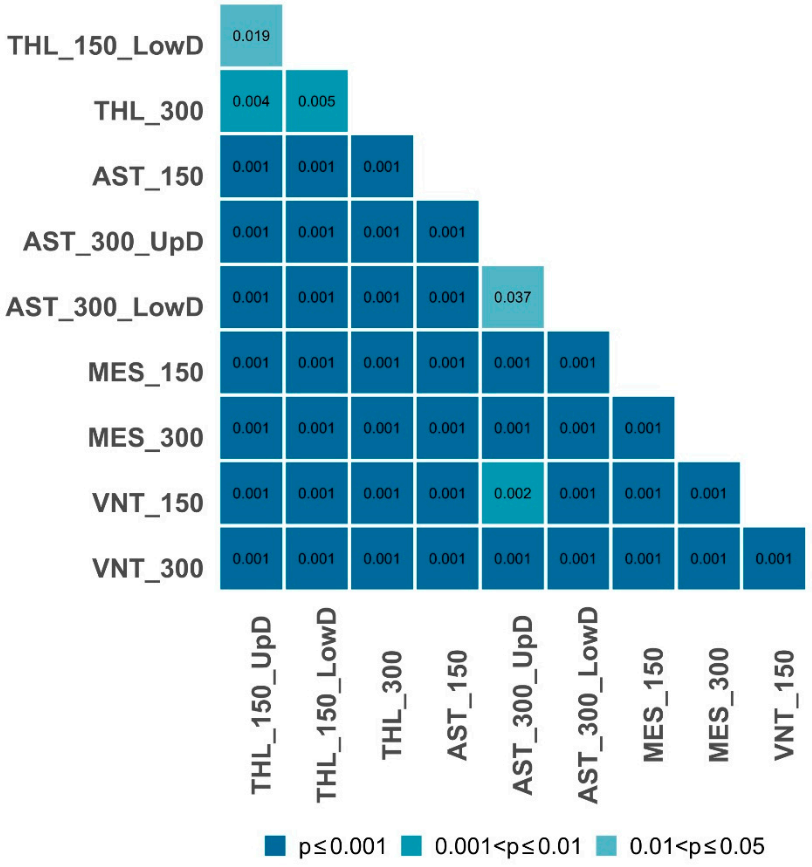

Supplementary Figure S3: Pairwise comparison of PERMANOVA *p*-values between sample groups after merging.

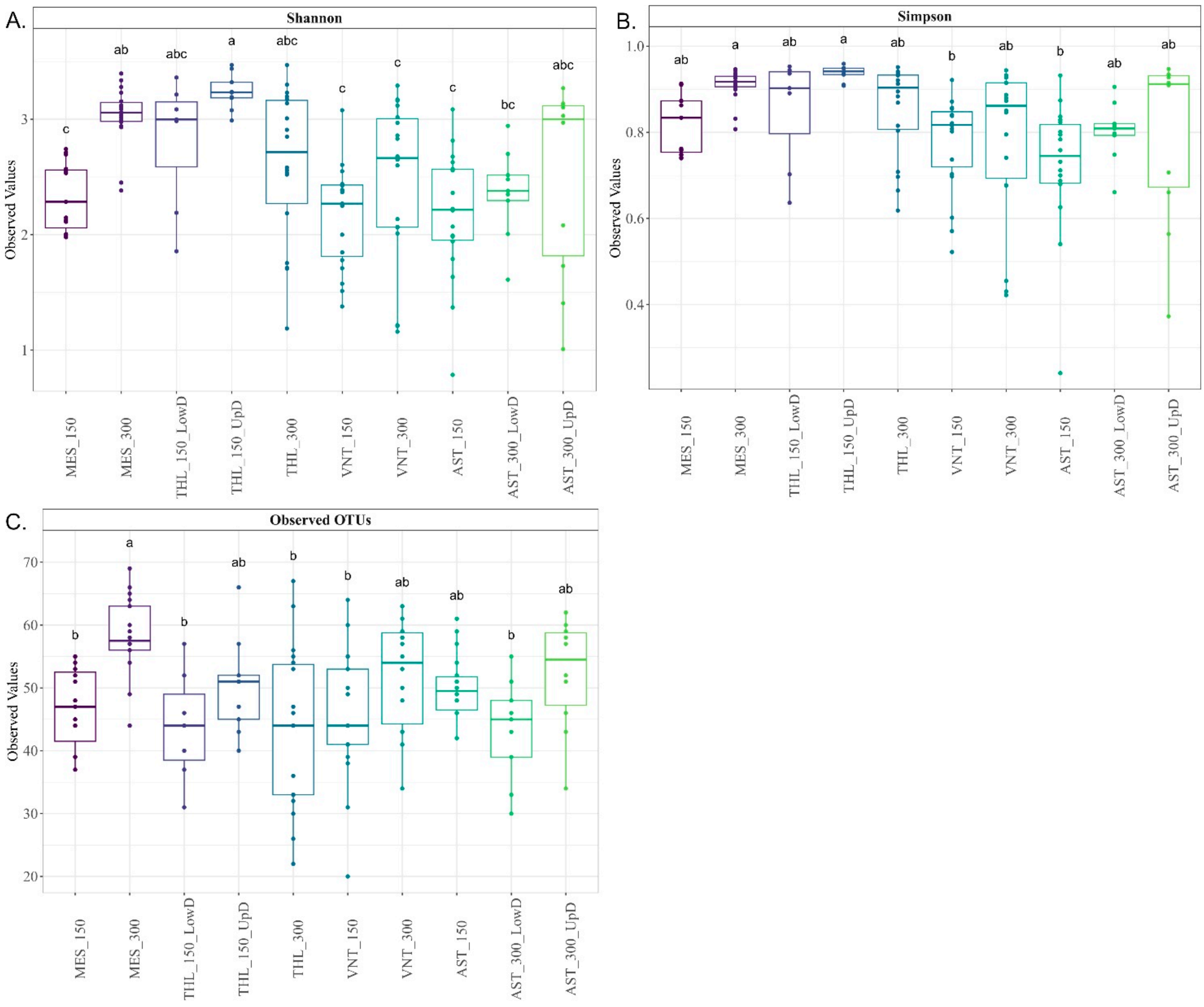

Supplementary Figure S4. Alpha diversity of bacterial communities associated with the gastrointestinal tract of wild and farmed fish. Different letters denote statistically significant differences between groups.

Supplementary Table S5. Alpha diversity of bacterial communities associated with the gastrointestinal tract of wild and farmed fish.

**Overall comparison**	**Shannon**	**Simpson**	**Observed OTUs**
Wild	2.77±0.06 [a]	0.87±0.01 [a]	49.12±1.22 [a]
Domesticated	2.3±0.07 [b]	0.77±0.02 [b]	48.92±1.03 [a]
**Sample Groups**	**Shannon**	**Simpson**	**Observed OTUs**
MES_150	2.33±0.07 [c]	0.82±0.02[ab]	46.8±1.46 [b]
MES_300	3.03±0.06 [ab]	0.91±0.01[a]	58.06±1.49 [a]
THL_150_LowD	2.81±0.13 [abc]	0.85±0.03 [ab]	43.86±2.03 [b]
THL_150_UpD	3.24±0.04 [a]	0.94±0 [a]	50.22±1.8 [ab]
THL_300	2.61±0.15 [abc]	0.85±0.02 [ab]	43.61±2.94 [b]
VNT_150	2.17±0.1 [c]	0.78±0.03 [b]	45.68±2.38 [b]
VNT_300	2.46±0.16 [c]	0.78±0.04 [ab]	52.17±1.99 [ab]

AST_150	2.16±0.13 [c]	0.72±0.04 [b]	50.06±1.19 [ab]
AST_300_LowD	2.36±0.09 [bc]	0.8±0.02 [ab]	43.33±1.87 [b]
AST_300_UpD	2.49±0.19 [abc]	0.79±0.05 [ab]	52.2±2.05 [ab]

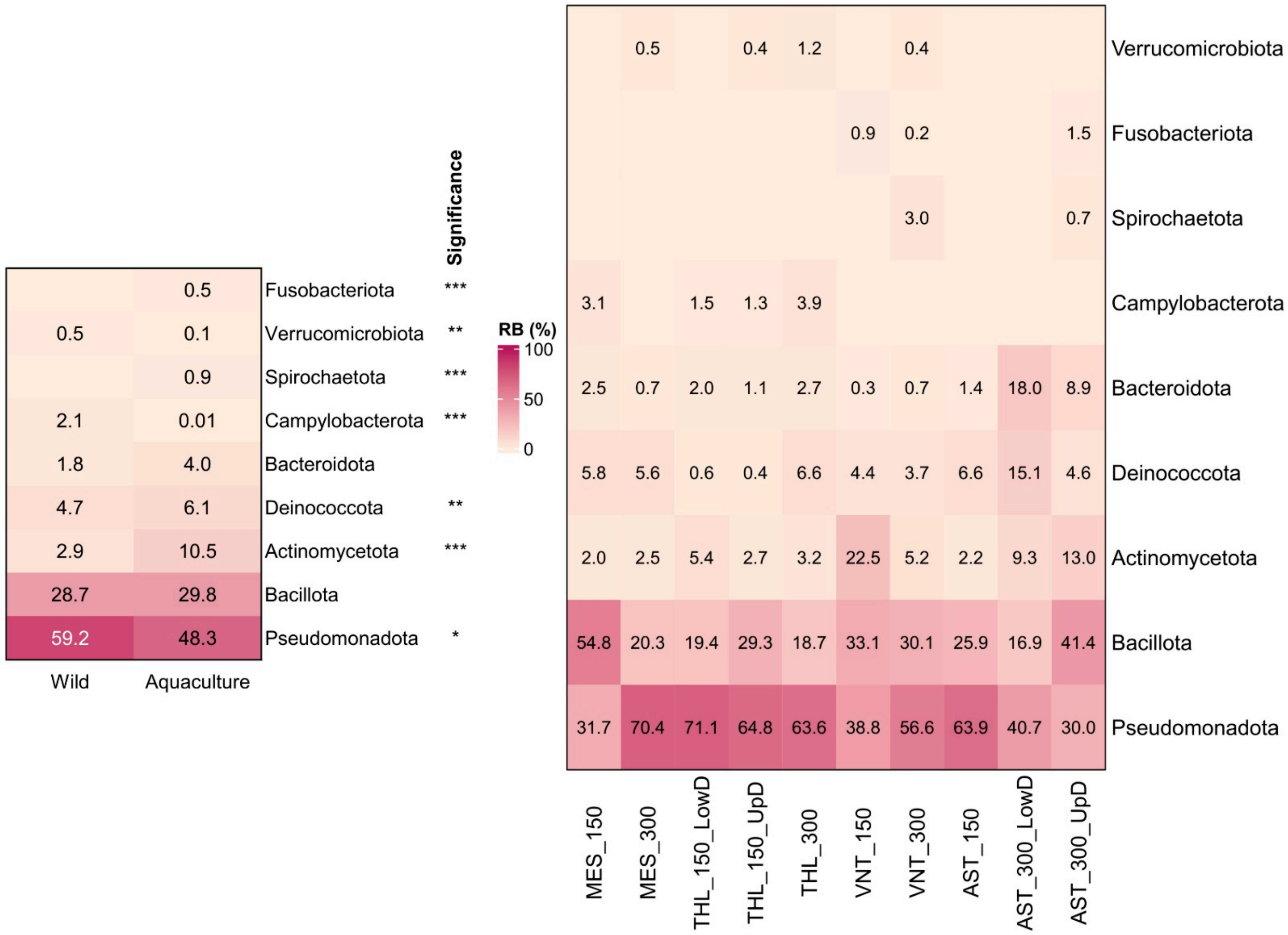

Supplementary Figure S5. Relative abundance of bacterial phyla identified in the gut samples of *S. aurata*. Statistical significance of observed differences in relative abundance was calculated using pairwise Wilcoxon rank-sum tests. Stars denote different p-value ranges. ***: p≤0.001, **: 0.001<p≤0.01, *: 0.01<p≤0.05.

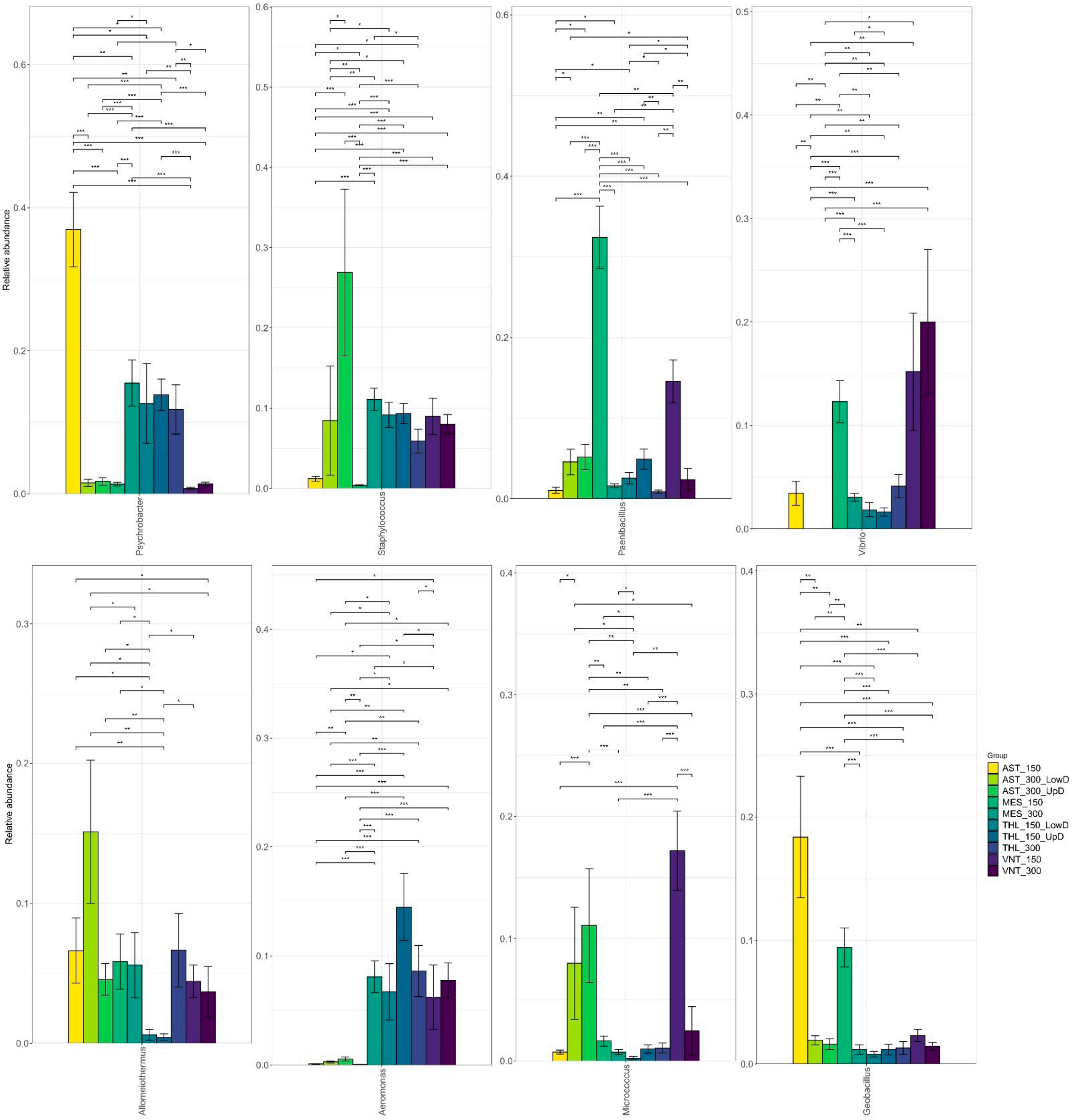

Supplementary Figure S6. Differentially abundant genera in the studied samples. To reduce complexity, the eight most abundant genera, involving 165 pairwise comparisons, are shown in the bar plots. Statistical significance of observed differences in relative abundance was calculated using pairwise Wilcoxon rank-sum tests. Stars denote different p-value ranges. ***: p≤0.001, **: 0.001<p≤0.01, *: 0.01<p≤0.05.

	Origin			Wild			Aquaculture			MES			THL			VNT			AST		
Genus	Wild	AqC	(S)	MES	THL	(S)	VNT	AST	(S)	150 g	300 g	(S)	150 g	300 g	(S)	150 g	300 g	(S)	150 g	300 g	(S)
Psychrobacter	10.8	9.9	**	9.1	12.5		1.0	18.8	***	1.3	15.5	***	13.3	11.8		0.7	1.4		37.0	1.6	***
Staphylococcus	6.9	9.2		6.2	7.5		8.5	9.9	***	0.4	11.1	***	9.3	5.9		9.0	8.0		1.2	18.2	**
Paenibacillus	8.9	5.8		15.6	2.3	**	8.6	3.0		32.4	1.6	***	3.9	0.9	**	14.5	2.4	**	1.0	4.9	**
Vibrio	5.1	9.6	**	7.3	3.0	**	17.5	1.7	***	12.3	3.1	***	1.7	4.1		15.2	20.0		3.5		***
Allomeiothermus	4.7	6.1	**	5.7	3.8		4.1	8.1	*	5.8	5.6		0.5	6.6	*	4.4	3.7		6.6	9.6	
Aeromonas	7.1	3.6	***	4.4	9.8	*	7.0	0.3	**	0.1	8.1	***	11.1	8.6		6.2	7.8		0.1	0.4	**
Geobacillus	3.0	5.9	*	4.9	1.2	*	1.9	9.8	**	9.4	1.2	***	1.0	1.3		2.3	1.4		18.4	1.8	***
Micrococcus	1.0	7.7	***	1.1	0.9		10.0	5.3		1.6	0.7		0.6	1.1		17.2	2.5	***	0.7	9.6	***
Enterobacter	4.6	3.1		6.5	2.8		4.4	1.8		0.1	11.8	***	2.2	3.4		0.9	8.0	***	0.6	2.9	*
Acinetobacter	3.9	2.8	*	4.5	3.3		1.9	3.6	**	4.0	4.9		5.3	1.6	**	2.8	0.9	*	3.6	3.7	
Pseudomonas	3.8	2.8	***	3.8	3.8		3.2	2.4		3.8	3.8		4.6	3.0		2.0	4.5		2.7	2.1	
Caldimonas	2.8	2.6		2.3	3.4		1.5	3.7		1.2	3.1		0.5	5.9		0.9	2.1		1.2	6.1	
Halalkalibacter	1.7	3.6	***	3.5		***	6.3	0.9	***	7.6		***				3.6	9.2		0.1	1.7	
Photobacterium	3.9	1.3	***	1.3	6.4	**	2.5		***	0.7	1.8	**	11.1	2.3		2.1	2.8			0.1	
Pantoea	2.7	2.3		3.7	1.7		2.0	2.5		0.1	6.7	***	2.0	1.4		0.8	3.2	*	2.5	2.6	
Bacillus	3.2	1.4	**	1.9	4.4		1.8	0.9	*	0.8	2.7	***	4.0	4.8		1.3	2.3	**	1.4	0.5	*
Wautersiella	0.1	2.9	***		0.1		0.1	5.7	***					0.2		0.2			1.2	9.9	
Undibacterium	1.8	0.4		0.4	3.2		0.8		**		0.7	**		6.1		0.4	1.3				
Alkalihalophilus	0.7	1.2	***	1.5		***	2.0	0.4	***	3.3		***				1.2	2.7			0.7	
Streptococcus	1.8	0.1	***	0.1	3.5	**	0.1				0.2	*	1.9	4.9		0.1	0.1		0.1		
Escherichia-Shigella	0.6	0.9		0.8	0.3	**	1.1	0.8		1.5	0.3	*	0.2	0.3		1.7	0.4	**	0.5	1.0	
Glutamicibacter	1.1	0.3	**	0.7	1.5		0.5	0.1			1.3	***	2.5	0.5		0.1	0.9	***	0.1		
Delftia	0.2	1.1	***	0.1	0.3		0.1	2.2	***	0.1			0.5	0.1		0.1	0.1		2.6	1.8	
Clostridium		1.2	**				1.6	0.9	*				0.1				3.2		1.8	0.1	**
Shewanella	0.5	0.7	***	0.4	0.6		1.5		***	0.1	0.6	**	0.8	0.5		2.3	0.6				
Arcobacter	1.2		**	1.4	1.0					3.1			0.1	1.9							
Enterococcus	0.8	0.4	***	0.9	0.7		0.1	0.6	***		1.6		1.5			0.2		**	0.8	0.5	
Rheinheimera	1.1			1.9	0.3						3.4			0.6							
Providencia	0.2	0.9	***	0.1	0.2		0.2	1.6	**	0.2	0.1	*	0.4	0.1	**	0.2	0.2		0.4	2.7	*
Serratia	0.9	0.2		0.5	1.2		0.2	0.3			1.0	***	2.3	0.1	**	0.1	0.2		0.3	0.3	
Brevundimonas	0.4	0.6		0.2	0.5			1.2	***	0.1	0.3		0.2	0.8					0.7	1.6	
Methylorubrum	0.9	0.1	***	0.2	1.6	**	0.1			0.1	0.3	*	2.4	0.9		0.1	0.2				
Stenotrophomonas	0.5	0.4		0.3	0.7		0.3	0.6		0.3	0.3		0.8	0.7		0.2	0.4		0.4	0.9	
Buchnera	0.8	0.2	***	0.2	1.3	***	0.3		**		0.4	***	1.7	1.0		0.1	0.6	***		0.1	

RB (%)
40
20
0

Supplementary Figure S7. Differences in abundance of bacterial genera based on the origin, populations and growth phase of collected samples. Statistical significance (S) was calculated using pairwise Wilcoxon rank-sum tests. Stars denote different p-value ranges. ***: p≤0.001, **: 0.001<p≤0.01, *: 0.01<p≤0.05. AqC: Aquaculture; MES: Messolonghi; THL: Tholi; VNT: Vonitsa; AST: Astakos.

Supplementary Table S6. Core and unique bacteria in gut samples of *S. aurata* based on the collection area. MES: Messolonghi; THL; Tholi; VNT: Vonitsa; AST: Astakos.

Taxa	Phylum	Genus	Messolonghi	Tholi	Vonitsa	Astakos	Type
Otu10	Pseudomonadota	Enterobacter	1	1	1	1	Core
Otu11	Pseudomonadota	Aeromonas	1	1	1	1	Core
Otu15	Bacillota	Geobacillus	1	1	1	1	Core
Otu16	Bacillota	Bacillus	1	1	1	1	Core
Otu31	Pseudomonadota	Pantoea	1	1	1	1	Core
Otu37	Pseudomonadota	Acinetobacter	1	1	1	1	Core
Otu4	Pseudomonadota	Psychrobacter	1	1	1	1	Core
Otu8	Bacillota	Staphylococcus	1	1	1	1	Core
Otu178	Pseudomonadota	Acinetobacter	1	1	0	1	MES-THL-AST
Otu117	Pseudomonadota	Stenotrophomonas	1	1	0	0	Wild
Otu1573	Pseudomonadota	Psychrobacter	1	1	0	0	Wild
Otu34	Bacillota	Bacillus	1	1	0	0	Wild
Otu76	Pseudomonadota	Pseudomonas	1	1	0	0	Wild
Otu2	Deinococcota	Allomeiothermus	1	0	1	1	MES-VNT-AST
Otu24	Pseudomonadota	Pseudomonas	1	0	1	1	MES-VNT-AST
Otu30	Pseudomonadota	Pseudomonas	1	0	1	1	MES-VNT-AST
Otu6	Actinomycetota	Micrococcus	1	0	1	1	MES-VNT-AST
Otu9	Pseudomonadota	Caldimonas	1	0	1	1	MES-VNT-AST
Otu187	Pseudomonadota	Pseudomonas	1	0	1	0	MES-VNT
Otu87	Pseudomonadota	Vibrio	1	0	1	0	MES-VNT
Otu113	Pseudomonadota	Pantoea	1	0	0	1	MES-AST
Otu38	Actinomycetota	Dietzia	1	0	0	1	MES-AST
Otu60	Bacillota	Brevibacillus	1	0	0	1	MES-AST
Otu2349	Pseudomonadota	Pseudomonas	1	0	0	0	MES
Otu27	Pseudomonadota	Shewanella	1	0	0	0	MES
Otu42	Actinomycetota	Glutamicibacter	1	0	0	0	MES
Otu75	Pseudomonadota	Stenotrophomonas	1	0	0	0	MES
Otu88	Pseudomonadota	Rhizobium	1	0	0	0	MES
Otu99	Pseudomonadota	Paracoccus	1	0	0	0	MES
Otu907	Bacillota	Staphylococcus	0	1	1	0	THL-VNT
Otu1527	Pseudomonadota	Aeromonas	0	1	0	0	THL
Otu1773	Pseudomonadota	Aeromonas	0	1	0	0	THL
Otu2003	Pseudomonadota	Aeromonas	0	1	0	0	THL
Otu36	Bacillota	Paenibacillus	0	1	0	0	THL
Otu45	Pseudomonadota	Methylorubrum	0	1	0	0	THL
Otu46	Pseudomonadota	Buchnera	0	1	0	0	THL
Otu47	Pseudomonadota	Photobacterium	0	1	0	0	THL
Otu49	Pseudomonadota	Diaphorobacter	0	1	0	0	THL
Otu871	Pseudomonadota	Enterobacter	0	1	0	0	THL
Otu77	Pseudomonadota	Providencia	0	0	1	1	Aquaculture
Otu1120	Bacillota	Alkalihalophilus	0	0	1	0	VNT
Otu1967	Pseudomonadota	Escherichia-Shigella	0	0	1	0	VNT
Otu7	Bacillota	Halalkalibacter	0	0	1	0	VNT
Otu19	Pseudomonadota	Acinetobacter	0	0	0	1	AST

Otu20	Bacillota	Paenibacillus	0	0	0	1	AST
Otu26	Pseudomonadota	Delftia	0	0	0	1	AST
Otu54	Pseudomonadota	Brucella	0	0	0	1	AST
Otu55	Actinomycetota	Cutibacterium	0	0	0	1	AST
Otu56	Pseudomonadota	Methylobacterium	0	0	0	1	AST
Otu62	Pseudomonadota	Ralstonia	0	0	0	1	AST
Otu70	Pseudomonadota	Methylibium	0	0	0	1	AST
Otu89	Bacillota	Enterococcus	0	0	0	1	AST
Otu98	Pseudomonadota	Sphingomonas	0	0	0	1	AST

Supplementary Table S7. Core and unique bacteria in the gastrointestinal tract of *S. aurata* according to the body weight. Wild_150: 150-gram wild fish; Wild_300: 300-gram wild fish; AqC_150: 150-gram aquaculture fish; AqC_300: 300-gram aquaculture fish.

**Taxa**	**Phylum**	**Genus**	**Wild_150**	**Wild_300**	**AqC_150**	**AqC_300**	**Type**
Otu15	Bacillota	Geobacillus	1	1	1	1	Core
Otu16	Bacillota	Bacillus	1	1	1	1	Core
Otu24	Pseudomonadota	Pseudomonas	1	1	1	1	Core
Otu30	Pseudomonadota	Pseudomonas	1	1	1	1	Core
Otu31	Pseudomonadota	Pantoea	1	1	1	1	Core
Otu37	Pseudomonadota	Acinetobacter	1	1	1	1	Core
Otu4	Pseudomonadota	Psychrobacter	1	1	1	1	Core
Otu6	Actinomycetota	Micrococcus	1	1	1	1	Core
Otu8	Bacillota	Staphylococcus	1	1	1	1	Core
Otu178	Pseudomonadota	Acinetobacter	1	1	1	0	Wild-AqC_150
Otu76	Pseudomonadota	Pseudomonas	1	1	0	1	Wild-AqC_300
Otu1573	Pseudomonadota	Psychrobacter	1	1	0	0	Wild
Otu77	Pseudomonadota	Providencia	1	0	1	1	Wild_150-AqC
Otu117	Pseudomonadota	Stenotrophomonas	1	0	0	1	Wild_150-AqC_300
Otu10	Pseudomonadota	Enterobacter	0	1	1	1	Wild_300-AqC
Otu11	Pseudomonadota	Aeromonas	0	1	1	1	Wild_300-AqC
Otu113	Pseudomonadota	Pantoea	0	1	1	1	Wild_300-AqC
Otu2	Deinococcota	Allomeiothermus	0	1	1	1	Wild_300-AqC
Otu9	Pseudomonadota	Caldimonas	0	1	1	1	Wild_300-AqC
Otu187	Pseudomonadota	Pseudomonas	0	1	1	0	Wild_300-AqC_150
Otu871	Pseudomonadota	Enterobacter	0	1	0	1	Wild_300-AqC_300
Otu907	Bacillota	Staphylococcus	0	1	0	1	Wild_300-AqC_300
Otu118	Pseudomonadota	Vibrio	0	1	0	0	Wild_300
Otu1527	Pseudomonadota	Aeromonas	0	1	0	0	Wild_300
Otu1773	Pseudomonadota	Aeromonas	0	1	0	0	Wild_300
Otu2003	Pseudomonadota	Aeromonas	0	1	0	0	Wild_300
Otu2349	Pseudomonadota	Pseudomonas	0	1	0	0	Wild_300
Otu27	Pseudomonadota	Shewanella	0	1	0	0	Wild_300
Otu34	Bacillota	Bacillus	0	1	0	0	Wild_300
Otu36	Bacillota	Paenibacillus	0	1	0	0	Wild_300
Otu42	Actinomycetota	Glutamicibacter	0	1	0	0	Wild_300
Otu45	Pseudomonadota	Methylorubrum	0	1	0	0	Wild_300
Otu46	Pseudomonadota	Buchnera	0	1	0	0	Wild_300
Otu47	Pseudomonadota	Photobacterium	0	1	0	0	Wild_300

Otu49	Pseudomonadota	Diaphorobacter	0	1	0	0	Wild_300
Otu87	Pseudomonadota	Vibrio	0	1	0	0	Wild_300
Otu99	Pseudomonadota	Paracoccus	0	1	0	0	Wild_300
Otu20	Bacillota	Paenibacillus	0	0	1	1	Aquaculture
Otu26	Pseudomonadota	Delftia	0	0	1	1	Aquaculture
Otu19	Pseudomonadota	Acinetobacter	0	0	1	0	AqC_150
Otu38	Actinomycetota	Dietzia	0	0	1	0	AqC_150
Otu44	Pseudomonadota	Escherichia-Shigella	0	0	1	0	AqC_150
Otu55	Actinomycetota	Cutibacterium	0	0	1	0	AqC_150
Otu56	Pseudomonadota	Methylobacterium	0	0	1	0	AqC_150
Otu60	Bacillota	Brevibacillus	0	0	1	0	AqC_150
Otu7	Bacillota	Halalkalibacter	0	0	1	0	AqC_150
Otu88	Pseudomonadota	Rhizobium	0	0	1	0	AqC_150
Otu89	Bacillota	Enterococcus	0	0	1	0	AqC_150
Otu98	Pseudomonadota	Sphingomonas	0	0	1	0	AqC_150
Otu62	Pseudomonadota	Ralstonia	0	0	0	1	AqC_300

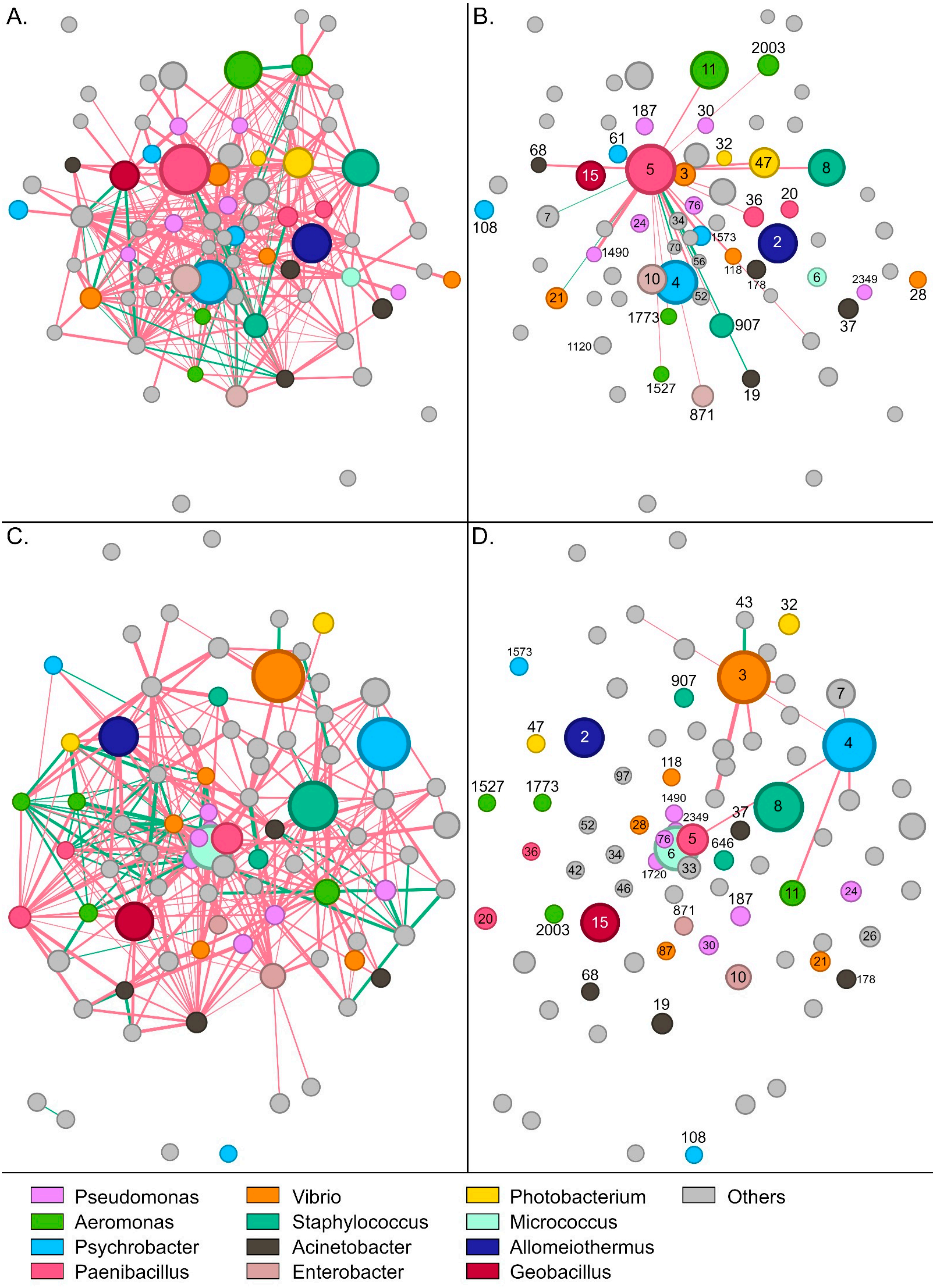
A.
B.
C.
D.
Pseudomonas
Aeromonas
Psychrobacter
Paenibacillus
Vibrio
Staphylococcus
Acinetobacter
Enterobacter
Photobacterium
Micrococcus
Allomeiothermus
Geobacillus
Others

Supplementary Figure S8. Significant interactions between bacterial OTUs. Nodes (circles) represent bacterial OTUs and edges (lines) interactions. Red edges correspond to mutual exclusions and green edges copresence. (A) The complete network of interactions within the microbiota of wild samples. (B) Interactions formed by *Paenibacillus*-5, the prevalent bacterium in wild samples. (C) The complete network of interactions between community members in aquaculture samples. (D) The interactions of the two most abundant bacteria in aquaculture samples, *Psychrobacter*-4 and *Vibrio*-3.

Wild samples				Aquaculture samples					
Messolonghi (1)		Tholi (2)		Vonitsa (3)		Astakos (4)			
UpD	LowD	UpD	LowD	UpD	LowD	UpD	LowD	Species	Phylum
1.8								Arthrobacter glacialis	Actinomycetota
	2.2							Corynebacterium mucifaciens	
3.6								Glutamicibacter bergerei	
	2.2			7.4	5.3			Micrococcus aloeverae	
		4.8						Rothia mucilaginosa	
		4.8						Bacillus infantis	Bacillota
	4.3			18.5	21.1	45.8	69.2	Bacillus subtilis	
1.8	2.2							Enterococcus faecalis	
1.8								Enterococcus faecium	
1.8								Exiguobacterium artemiae	
1.8	4.3	9.5						Niallia alba	
		4.8						Paenibacillus etheri	
1.8				3.7				Staphylococcus aureus	
1.8								Staphylococcus borealis	
	2.2	4.8		18.5	10.5	4.2	7.7	Staphylococcus epidermidis	
1.8						16.7	7.7	Staphylococcus hominis	
1.8		4.8						Staphylococcus saprophyticus	
1.8		4.8		7.4				Staphylococcus taiwanensis	
		4.8		33.3				Staphylococcus ureilyticus	
1.8	4.3	4.8		3.7	15.8		7.7	Staphylococcus warneri	
1.8	4.3	4.8	20.0					Staphylococcus xylosus	
	2.2							Streptococcus cristatus	
	2.2							Streptococcus downii	
		4.8						Terribacillus saccharophilus	
1.8								Chryseobacterium indoltheticum	Bacteroidota
1.8								Chryseobacterium salivictor	
	2.2							Kaistella antarctica	
	2.2							Sphingobacterium faecium	
3.6								Acinetobacter johnsonii	Pseudomonadota
			6.7					Aeromonas molluscorum	
	4.3							Aeromonas rivipollensis	
	4.3		6.7					Aeromonas salmonicida	
			6.7					Buttiauxella massiliensis	
3.6								Erwinia persicina	
3.6								Erwinia rhapontici	
	2.2		6.7					Kluyvera cryocrescens	
1.8								Pantoea agglomerans	
	2.2							Pantoea ananatis	
	2.2							Pantoea coffeiphila	
	2.2							Pantoea endophytica	
	2.2	4.8	6.7			4.2	7.7	Photobacterium carnosum	
	2.2							Photobacterium damselae	
	4.3	4.8	6.7					Photobacterium piscicola	
1.8								Pseudomonas alvandae	
		4.8						Pseudomonas brenneri	
9.1			6.7					Pseudomonas crudilactis	
1.8								Pseudomonas japonica	
3.6								Pseudomonas kielensis	
1.8								Pseudomonas luteola	
1.8								Pseudomonas migulae	
1.8	2.2							Pseudomonas poae	
3.6	6.5							Pseudomonas promysalinigenes	
1.8	2.2	4.8						Pseudomonas psychrophila	
1.8								Pseudomonas rhodesiae	
7.3								Pseudomonas seleniipraecipitans	
1.8	2.2							Psychrobacter cryohalolentis	
1.8	4.3		6.7	7.4	47.4	29.2		Psychrobacter pulmonis	
7.3	6.5							Shewanella vaxholmensis	
1.8								Stenotrophomonas chelatiphaga	
3.6	2.2							Stenotrophomonas maltophilia	
		4.8	20.0					Vibrio alfacsensis	
		4.8						Vibrio anguillarum	
	2.2	4.8	6.7					Vibrio gigantis	
5.5		4.8						Vibrio harveyi	
	2.2							Vibrio ichthyoenteri	
	4.3							Vibrio owensii	
		4.8						Vibrio rotiferianus	
	2.2							Vibrio rumoiensis	
	2.2							Vibrio scophthalmi	

RA (%)
100
50
0

Supplementary Figure S9. The relative abundance of bacterial strains isolated from the gut tissue of *S. aurata* samples. UpD: Upper digestive tract; LowD: Lower digestive tract.

Supplementary Table S8. The number and the relative abundance of isolated bacteria from wild and aquaculture samples based on their taxonomic assignment at the genus level.

Genus	No. of strains	No. of strains in wild	Wild (%)	No. of strains in aquaculture	Aquaculture %
Acinetobacter	2	2	1.5	0	0.0
Aeromonas	6	6	4.4	0	0.0
Arthrobacter	1	1	0.7	0	0.0
Glutamicibacter	2	2	1.5	0	0.0
Bacillus	32	3	2.2	29	34.9
Niallia	5	5	3.6	0	0.0
Buttiauxella	1	1	0.7	0	0.0
Chryseobacterium	2	2	1.5	0	0.0
Kaistella	1	1	0.7	0	0.0
Corynebacterium	1	1	0.7	0	0.0
Pantoea	4	4	2.9	0	0.0
Enterococcus	3	3	2.2	0	0.0
Erwinia	4	4	2.9	0	0.0
Exiguobacterium	1	1	0.7	0	0.0
Micrococcus	4	1	0.7	3	3.6
Paenibacillus	1	1	0.7	0	0.0
Photobacterium	10	8	5.8	2	2.4
Kluyvera	2	2	1.5	0	0.0
Pseudomonas	28	28	20.4	0	0.0
Psychrobacter	24	6	4.4	18	21.7
Rothia	1	1	0.7	0	0.0
Shewanella	7	7	5.1	0	0.0
Sphingobacterium	1	1	0.7	0	0.0
Staphylococcus	52	21	15.3	31	37.3
Stenotrophomonas	4	4	2.9	0	0.0
Streptococcus	2	2	1.5	0	0.0
Terribacillus	1	1	0.7	0	0.0
Vibrio	18	18	13.1	0	0.0
**Total**	**220**	**137**	**100**	**83**	**100**
